# Supplementary material for: Prospective surveillance study to detect antimalarial drug resistance, gene deletions of diagnostic relevance and genetic diversity of Plasmodium falciparum in Mozambique: protocol
Source: BMJ Open. 2022 Jul 11;12(7):e063456. doi: 10.1136/bmjopen-2022-063456 (PMC9274532; doi:10.1136/bmjopen-2022-063456)
Supplement: Supplementary data [file bmjopen-2022-063456supp001.pdf]

Appendixes

Appendix 1. Information sheet and informed consent for participants over 18 years of age

Appendix 2. Information sheet and informed assent for minors aged between 12 and 17 years old

Appendix 3. Information sheet and informed consent for adult pregnant women

Appendix 4. Information sheet and informed assent for pregnant women between 12 and 18 years of age

Appendix 5. Questionnaire for Medium-high transmission area, children under 2-10 years old

Appendix 6. Questionnaire for Low transmission area, all ages

Appendix 7. Questionnaire for Pregnant women attending ANC clinic in medium-high transmission area

Appendix 8. Questionnaire for Pregnant women attending ANC clinic in low transmission area

Appendix 9. Procedures for the collection, handling and storage of dried blood samples on filter paper and rapid diagnostic tests (RDT).

Appendix 10. COVID-19 safety and research considerations

Appendix 11. Worksheet for Monitoring and Evaluation of Field Activities

Appendix 1. Information sheet and informed consent for participants over 18 years of age

PART I

INFORMATION SHEET AND INFORMED CONSENT FOR PARTICIPANTS OVER 18 YEARS OF AGE

Name of Affiliated Institutions

1. Manhiça Health Research Center (CISM), Manhiça, Mozambique
2. Malaria Consortium, Maputo, Mozambique
3. Barcelona Global Health Institute (ISGlobal), Barcelona, Spain
4. National Malaria Control Programme, Ministry of Health, Maputo, Mozambique
5. University of California, San Francisco, USA
6. Clinton Health Access Initiative, Boston, USA
7. Institute of Disease Modeling, Bill and Melinda Gates Foundation, Seattle, USA
8. Bill and Melinda Gates Foundation, Seattle, USA

Protocol title and version: "A prospective surveillance study to detect antimalarial drug resistance, gene deletions of diagnostic relevance and genetic diversity of *Plasmodium falciparum* in Mozambique," version number 7, 25 August 2021.

Name and affiliation of Principal Investigator(s): Baltazar Candrinho, National Malaria Control Programme, Ministry of Health, Maputo, Mozambique and Alfredo Mayor, Manhiça Health Research Center, Manhiça, Mozambique.

Study funder: Bill and Melinda Gates Foundation, USA

Introduction: The National Malaria Control Programme in partnership with Malaria Consortium and the Manhiça Health Research Center are conducting a study to analyse the genetics of malaria parasites to identify the best ways to control and/or eliminate this disease from the country.

Please read this form with care. This form provides important information about participating in this study. All the information which follows, discussed below, is to allow you to understand what the study involves and the steps that would need your collaboration, so that before becoming involved in the study, you can decide freely if you wish to participate.

You can take the time that you feel necessary to decide about your participation in this study. If you have questions about the study, or any part of this form, please ask us. If you decide to participate in this research, you will be asked to sign this form. One copy of the signed form will be provided to you for your records. If at any time you feel that you do not understand the information that is being provided, please do not hesitate to interrupt so that we can explain and clarify everything again.

After receiving your consent to participate, we will ask you some personal questions about your age, date of birth, recent illnesses, including history of fever, occupation, travel history, residence, use of insecticide treated mosquito nets or taking of antimalarial medications in the last month and then we will take a few drops of blood from your finger.

Rationale: Mozambique constitutes a main goal for the World Health Organization and partnership initiative, namely, Roll Back Malaria, to end malaria in the world. In this context, through involvement in regional malaria elimination initiatives, the use of molecular malaria surveillance data, as a complement to traditional surveillance information, can contribute to the elimination of malaria in Southern Mozambique and a reduction of the burden in the north of the country. However, there is a lack of malaria diagnostic and drug resistance data and other measures of the genetic diversity of the parasite that causes malaria in different transmission settings. Therefore, more evidence is needed to demonstrate the feasibility of using genetic data as a driver of the intensity of transmission in high transmission areas. Additionally, understanding the prevalence of diagnostic and drug resistance and genetic diversity will inform more appropriate and impactful interventions to reduce malaria morbidity and mortality in Mozambique. The integration of genetic data into routine

surveillance activities has the potential to increase knowledge for programmatic decision-making on the optimal combination of control and elimination measures in Mozambique.

Research objectives: Your participation in this study will help us to identify the prevalence of molecular markers of antimalarial resistance along with other genetic markers, which will inform the National Malaria Control Programme to support decision-making on the use of antimalarials and best strategies to control and eliminate malaria in the country.

Type of research/Intervention: This is prospective, operational surveillance research.

Selection of participants: You were invited to participate in this research because you are part of a group that is the focus of this study: **adults over 18 years of age** with malaria, confirmed by a rapid diagnostic test, living in this region.

Voluntary participation: It's your choice if you want to participate in this study or not. Refusing to participate or withdrawing your participation will not result in any penalty or loss of health benefits or services. You will continue to receive medical care if you choose not to participate in this study. Your decision will not change the care that you receive now or in the future. Participating in this study is your choice. If you decide to participate in this study, you can leave at any time without consequences. If you want to stop participating in the study, just let the research team know.

Procedures: We will take a few drops of blood from your finger and four drops will be placed on two small pieces of paper (filter paper), two drops on each paper. The filter papers containing four drops of blood each will be kept in the Health Unit and sent to Manhiça Health Research Center where the analysis will be done. If necessary, the filter papers may be sent to a laboratory located outside of Mozambique (specifically, the ISGlobal laboratory in Spain or the University of California, San Francisco laboratory, in the United States) for additional analysis and molecular characterisation of the malaria parasites (alleles related to antimalarial resistance as well as genetic composition and other molecular markers of relevance to malaria surveillance, both in the parasite and human host). The filter papers will be stored by the Manhiça Health Research Center for future human and parasite malaria molecular studies for a period of up to 10 years. In addition to drops of blood, all participants will also be asked about their age, date of birth, recent illnesses, including history of fever, occupation, travel history, residence, use of insecticide treated mosquito nets or antimalaria medication taking in the past 24/48 hours.

Risks, Discomfort and Inconvenience: You may feel a little pain or fear when your finger is pricked. The pain will dissipate within a few hours.

Benefits: There are no direct benefits for you to participate in this study. However, the findings generated from the study will inform the National Malaria Control Programme in decision-making about the use of antimalarials and the best strategies to control and eliminate malaria from the country.

Costs of Participation/Compensation: You will not receive any money or compensation to take part in this study.

Privacy: The data collected will be anonymous, however the data obtained in this study may be shared with collaborating partners: The National Malaria Control Programme, Malaria Consortium Mozambique, Manhiça Health Research Center, ISGlobal, Institute of Disease Modeling and the University of California, San Francisco, USA. In relation to the DNA sequences of the malaria parasite, or your personal data, these will be archived in an online database that can be shared with other scientists and researchers when the data are sent to scientific publications to report the results of this study.

Confidentiality: The information collected will be kept confidential and only the study team will have access to individuals' information. The results of the study will be published and made available so that other interested people can learn from our study, but confidential information will not be shared in any circumstance. Your data will be completely anonymised.

Sharing of results: Results from this research will be shared on open access platforms online, in public data repositories or directly in scientific publications, in order to facilitate further collaboration, enhance trust in the findings and goodwill among researchers. We will specifically focus on data sharing among other African countries in the region which are engaging in similar approaches to the molecular surveillance of malaria.

Whom to Contact (Investigators and Ethics Committee): in case of any of these situations:

- If your questions, concerns or complaints are not being addressed by the research team;
- If you are unable to contact the research team;
- If you would like to speak with someone who is not part of the research team;
- If you have questions about your rights as a research participant;
- If you wish to obtain information or provide information about this research; or
- If you think that the study has caused harm.

Please return to the Health Unit and speak with the workers involved in the study or contact the study focal person, assigned by Malaria Consortium Mozambique, Neide Canana on telephone number: 860450563, or you can contact her at: Malaria Consortium Mozambique, Sita Av. Lucas Elias Kumato nr. 118, Bairro da Sommerschield – Maputo City, Mozambique, or you can also contact Manhiça Health Research Center, located at: Street 12, Bairro Cambeve in Município da Manhiça Maputo Province, Mozambique, or by telephone: 21810002. In case you are not satisfied with the responses provided, you may also contact the National Bioethics for Health Committee, Ministry of Health, Mozambique on the numbers: 824066350/844693186.

Ethics Committee approval of this study: This study was approved by the Manhiça Health Research Center Institutional Bioethics Health Committee and the National Bioethics for Health Committee.

PART II
DECLARATION OF INFORMED CONSENT

Study Title: “A prospective surveillance study to detect antimalarial drug resistance, gene deletions of diagnostic relevance and genetic diversity of *Plasmodium falciparum* in Mozambique.”

Declaration: I have read the information provided in this consent form, including the risks and possible benefits. All my questions about the research have been answered satisfactorily. I understand that I am free to withdraw from the study at any time without repercussions or loss of benefits to which I am entitled.

I give my consent to participate in this study.

INFORMED CONSENT
If there is any part of this consent form that you do not understand, ask the investigator before you sign.

I, _____________________ (Name of participant) give my voluntary consent to participate in the study: “A prospective surveillance study to detect antimalarial drug resistance, gene deletions of diagnostic relevance and genetic diversity of *Plasmodium falciparum* in Mozambique.”

My questions have all been answered by _________________ (Name of researcher) in my own language. In case I have any other questions, I know that I can contact the study focal person assigned to Malaria Consortium and the National Bioethics for Health Committee through the contacts provided. I understand that I may withdraw my participation from the study, at any time for any reason, without any repercussions.

Do you allow your samples to be stored and used in future research? □ **Yes** □ **No**

I agree to take part in this study.

Signatures

Participant's fingerprint if they cannot sign

______________________________ _____________

Signature of participant Date and time

____

Name of participant (in capital letters)

____

Signature of the person who explained consent

___________________________________ _______________

Name of the person who explained consent (in capital letters) Date and time

If the participant does not know how to read, an impartial witness must also sign this form:

____ ____________

Signature of the impartial witness Date and time

____

Name of the impartial witness (in capital letters)

Appendix 2. Information sheet and informed assent for minors aged between 12 and 17 years old.

PART I

INFORMATION SHEET AND INFORMED ASSENT FOR MINORS AGED BETWEEN 12 AND 17 YEARS OLD

Name of Affiliated Institutions

9. Manhiça Health Research Center (CISM), Manhiça, Mozambique
10. Malaria Consortium, Maputo, Mozambique
11. Barcelona Global Health Institute (ISGlobal), Barcelona, Spain
12. National Malaria Control Programme, Ministry of Health, Maputo, Mozambique
13. University of California, San Francisco, USA
14. Clinton Health Access Initiative, Boston, USA
15. Institute of Disease Modeling, Bill and Melinda Gates Foundation, Seattle, USA
16. Bill and Melinda Gates Foundation, Seattle, USA

Protocol title and version: "A prospective surveillance study to detect antimalarial drug resistance, gene deletions of diagnostic relevance and genetic diversity of *Plasmodium falciparum* in Mozambique," version number 7, 25 August 2021.

Name and affiliation of Principal Investigator(s): Baltazar Candrinho, National Malaria Control Programme, Ministry of Health, Maputo, Mozambique and Alfredo Mayor, Manhiça Health Research Center, Manhiça, Mozambique.

Study funder: Bill and Melinda Gates Foundation, USA

Introduction: The National Malaria Control Programme in partnership with Malaria Consortium and the Manhiça Health Research Center are conducting a study to analyse the genetics of malaria parasites to identify the best ways to control and/or eliminate this disease from the country.

Please read this form with care. This form provides important information about participating in this study. All the information which follows, discussed below, is to allow you to understand what the study involves and the steps that would need your collaboration, so that before becoming involved in the study, you can decide freely if you wish to participate.

You can take the time that you feel necessary to decide about your participation in this study. If you have questions about the study, or any part of this form, please ask us. If you decide to participate in this research, you will be asked to sign this form. One copy of the signed form will be provided to you for your records. If at any time you feel that you do not understand the information that is being provided, please do not hesitate to interrupt so that we can explain and clarify everything again.

After receiving your consent to participate, we will ask you some personal questions about your age, date of birth, recent illnesses, including history of fever, occupation, travel history, residence, use of insecticide treated mosquito nets or taking of antimalarial medications in the last month and then we will take a few drops of blood from your finger.

Rationale: Mozambique constitutes a main goal for the World Health Organization and partnership initiative, namely, Roll Back Malaria, to end malaria in the world. In this context, through involvement in regional malaria elimination initiatives, the use of molecular malaria surveillance data, as a complement to traditional surveillance information, can contribute to the elimination of malaria in Southern Mozambique and a reduction of the burden in the north of the country. However, there is a lack of malaria diagnostic and drug resistance data and other measures of the genetic diversity of the parasite that causes malaria in different transmission settings. Therefore, more evidence is needed to demonstrate the feasibility of using genetic data as a driver of the intensity of transmission in high transmission areas. Additionally, understanding the prevalence of diagnostic and drug resistance and genetic diversity will inform more appropriate and impactful interventions

to reduce malaria morbidity and mortality in Mozambique. The integration of genetic data into routine surveillance activities has the potential to increase knowledge for programmatic decision-making on the optimal combination of control and elimination measures in Mozambique.

Research objectives: Your participation in this study will help us to identify the prevalence of molecular markers of antimalarial resistance along with other genetic markers, which will inform the National Malaria Control Programme to support decision-making on the use of antimalarials and best strategies to control and eliminate malaria in the country.

Type of research/Intervention: This is prospective, operational surveillance research.

Selection of participants: You were invited to participate in this research because you are part of a group that is the focus of this study: **minors aged between 0 and 18 years of age** with malaria, confirmed by a rapid diagnostic test, living in this region.

Voluntary participation: It's your choice if you want to participate in this study or not. Refusing to participate or withdrawing your participation will not result in any penalty or loss of health benefits or services. You will continue to receive medical care if you choose not to participate in this study. Your decision will not change the care that you receive now or in the future. Participating in this study is your choice. If you decide to participate in this study, you can leave at any time without consequences. If you want to stop participating in the study, just let the research team know.

Procedures: We will take a few drops of blood from your finger and four drops will be placed on two small pieces of paper (filter paper), two drops on each paper. The filter papers containing four drops of blood each will be kept in the Health Unit and sent to Manhiça Health Research Center where the analysis will be done. If necessary, the filter papers may be sent to a laboratory located outside of Mozambique (specifically, the ISGlobal laboratory in Spain or the University of California, San Francisco laboratory, in the United States) for additional analysis and molecular characterisation of the malaria parasites (alleles related to antimalarial resistance as well as genetic composition and other molecular markers of relevance to malaria surveillance, both in the parasite and human host). The filter papers will be stored by the Manhiça Health Research Center for future human and parasite malaria molecular studies for a period of up to 10 years. In addition to drops of blood, all participants will also be asked about their age, date of birth, recent illnesses, including history of fever, occupation, travel history, residence, use of insecticide treated mosquito nets or antimalaria medication taking in the past 24/48 hours.

Risks, Discomfort and Inconvenience: You may feel a little pain or fear when your finger is pricked. The pain will dissipate within a few hours.

Benefits: There are no direct benefits for you to participate in this study. However, the findings generated from the study will inform the National Malaria Control Programme in decision-making about the use of antimalarials and the best strategies to control and eliminate malaria from the country.

Costs of Participation/Compensation: You will not receive any money or compensation to take part in this study.

Privacy: The data collected will be anonymous, however the data obtained in this study may be shared with collaborating partners: the National Malaria Control Programme, Malaria Consortium Mozambique, Manhiça Health Research Center, ISGlobal, Institute of Disease Modeling and the University of California, San Francisco, USA. In relation to the DNA sequences of the malaria parasite, or your personal data, these will be archived in an online database that can be shared with other scientists and researchers when the data are sent to scientific publications to report the results of this study.

Confidentiality: The information collected will be kept confidential and only the study team will have access to individuals' information. The results of the study will be published and made available so that other interested

people can learn from our study, but confidential information will not be shared in any circumstance. Your data will be completely anonymised.

Sharing of results: Results from this research will be shared on open access platforms online, in public data repositories or directly in scientific publications, in order to facilitate further collaboration, enhance trust in the findings and goodwill among researchers. We will specifically focus on data sharing among other African countries in the region which are engaging in similar approaches to the molecular surveillance of malaria.

Whom to Contact (Investigators and Ethics Committee): in case of any of these situations:

- If your questions, concerns or complaints are not being addressed by the research team;
- If you are unable to contact the research team;
- If you would like to speak with someone who is not part of the research team;
- If you have questions about your rights as a research participant;
- If you wish to obtain information or provide information about this research; or
- If you think that the study has caused harm.

Please return to the Health Unit and speak with the workers involved in the study or contact the study focal person, assigned by Malaria Consortium Mozambique, Neide Canana on telephone number: 860450563, or you can contact her at: Malaria Consortium Mozambique, Sita Av. Lucas Elias Kumato nr. 118, Bairro da Sommerschield – Maputo City, Mozambique, or you can also contact Manhiça Health Research Center, located at: Street 12, Bairro Cambeve in Município da Manhiça Maputo Province, Mozambique, or by telephone: 21810002. In case you are not satisfied with the responses provided, you may also contact the National Bioethics for Health Committee, Ministry of Health, Mozambique on the numbers: 824066350/844693186.

Ethics Committee approval of this study: This study was approved by the Manhiça Health Research Center Institutional Bioethics Health Committee and the National Bioethics for Health Committee.

PART II

DECLARATION OF ASSENT

Study Title: "A prospective surveillance study to detect antimalarial drug resistance, gene deletions of diagnostic relevance and genetic diversity of *Plasmodium falciparum* in Mozambique."

Declaration: I have read the information provided in this assent form, including the risks and possible benefits. All of my questions about the research have been answered satisfactorily. I understand that I am free to withdraw from the study at any time without repercussions or loss of benefits to which I am entitled.

I give my assent to participate in this study.

INFORMED ASSENT

If there is any part of this assent form that you do not understand, ask the investigator before you sign.

I, _____________________ (Name of participant) give my voluntary assent to participate in the study: "A prospective surveillance study to detect antimalarial drug resistance, gene deletions of diagnostic relevance and genetic diversity of *Plasmodium falciparum* in Mozambique."

My questions have all been answered by _________________ (Name of researcher) in my own language. In case I have any other questions, I know that I can contact the study focal person assigned to Malaria Consortium and the National Bioethics for Health Committee through the contacts provided. I understand that I may withdraw my participation from the study, at any time for any reason, without any repercussions.

Mayor A, *et al. BMJ Open* 2022; 12:e063456. doi: 10.1136/bmjopen-2022-063456

Do you allow your samples to be stored and used in future research? □ **Yes** □ **No**

I agree to take part in this study.

Signatures

Minor's fingerprint if they cannot sign

______________________________ ____________
Signature of the minor Date and time

____
Minor's name (in capital letters)

____
Signature of the person who explained assent

_______________________________________ _______________
Name of the person who explained assent (in capital letters) Date and time

If the minor does not know how to read, an impartial witness must also sign this form:

____ ____________
Signature of the impartial witness Date and time

____
Name of the impartial witness (in capital letters)

PART III

INFORMATION SHEET AND INFORMED CONSENT FOR PARENTS/GUARDIANS OF MINOR PARTICIPANTS LESS THAN 18 YEARS OF AGE

Name of Affiliated Institutions

17. Manhiça Health Research Center (CISM), Manhiça, Mozambique
18. Malaria Consortium, Maputo, Mozambique
19. Barcelona Global Health Institute (ISGlobal), Barcelona, Spain
20. National Malaria Control Programme, Ministry of Health, Maputo, Mozambique
21. University of California, San Francisco, USA
22. Clinton Health Access Initiative, Boston, USA
23. Institute of Disease Modeling, Bill and Melinda Gates Foundation, Seattle, USA
24. Bill and Melinda Gates Foundation, Seattle, USA

Protocol title and version: "A prospective surveillance study to detect antimalarial drug resistance, gene deletions of diagnostic relevance and genetic diversity of *Plasmodium falciparum* in Mozambique," version number 7, 25 August 2021.

Name and affiliation of Principal Investigator(s): Baltazar Candrinho, National Malaria Control Programme, Ministry of Health, Maputo, Mozambique and Alfredo Mayor, Manhiça Health Research Center, Manhiça, Mozambique.

Study funder: Bill and Melinda Gates Foundation, USA

Introduction: The National Malaria Control Programme in partnership with Malaria Consortium and the Manhiça Health Research Center are conducting a study to analyse the genetics of malaria parasites to identify the best ways to control and/or eliminate this disease from the country.

Please read this form with care. This form provides important information about participating in this study. All the information which follows, discussed below, is to allow you to understand what the study involves and the steps that would need your collaboration, so that before becoming involved in the study, you can decide freely if you wish for your child to participate.

You can take the time that you feel necessary to decide about your child's participation in this study. If you have questions about the study, or any part of this form, please ask us. If you decide for your child to participate in this research, you will be asked to sign this form. One copy of the signed form will be provided to you for your records. If at any time you feel that you do not understand the information that is being provided, please do not hesitate to interrupt so that we can explain and clarify everything again.

After receiving your consent for your child to participate, we will ask them some personal questions about their age, date of birth, recent illnesses, including history of fever, occupation, travel history, residence, use of insecticide treated mosquito nets or taking of antimalarial medications in the last month and then we will take a few drops of blood from their finger.

Rationale: Mozambique constitutes a main goal for the World Health Organization and partnership initiative, namely, Roll Back Malaria, to end malaria in the world. In this context, through involvement in regional malaria elimination initiatives, the use of molecular malaria surveillance data, as a complement to traditional surveillance information, can contribute to the elimination of malaria in Southern Mozambique and a reduction of the burden in the north of the country. However, there is a lack of malaria diagnostic and drug resistance data and other measures of the genetic diversity of the parasite that causes malaria in different transmission settings. Therefore, more evidence is needed to demonstrate the feasibility of using genetic data as a driver of the intensity of transmission in high transmission areas. Additionally, understanding the prevalence of diagnostic and drug resistance and genetic diversity will inform more appropriate and impactful interventions to reduce malaria morbidity and mortality in Mozambique. The integration of genetic data into routine surveillance activities has the potential to increase knowledge for programmatic decision-making on the optimal combination of control and elimination measures in Mozambique.

Research objectives: Your child's participation in this study will help us to identify the prevalence of molecular markers of antimalarial resistance along with other genetic markers, which will inform the National Malaria Control Programme to support decision-making on the use of antimalarials and best strategies to control and eliminate malaria in the country.

Type of research/Intervention: This is prospective, operational surveillance research.

Selection of participants: Your child was invited to participate in this research because they are part of a group that is the focus of this study: **minors aged between 0 and 18 years of age** with malaria, confirmed by a rapid diagnostic test, living in this region.

Voluntary participation: It's your and your child's choice if you want them to participate in this study or not. Refusing to participate or withdrawing their participation will not result in any penalty or loss of health benefits or services. Your child will continue to receive medical care if you choose for them not to participate in this study. Your/their decision will not change the care that they receive now or in the future. Participating in this study is your/their choice. If you decide for your child to participate in this study, they can leave at any time without consequences. If they want to stop participating in the study, just let the research team know.

Procedures: We will take a few drops of blood from your child's finger and four drops will be placed on two small pieces of paper (filter paper), two drops on each paper. The filter papers containing four drops of blood each will be kept in the Health Unit and sent to Manhiça Health Research Center where the analysis will be done. If necessary, the filter papers may be sent to a laboratory located outside of Mozambique (specifically, the ISGlobal laboratory in Spain or the University of California, San Francisco laboratory, in the United States) for additional analysis and molecular characterisation of the malaria parasites (alleles related to antimalarial resistance as well as genetic composition and other molecular markers of relevance to malaria surveillance, both in the parasite and human host). The filter papers will be stored by the Manhiça Health Research Center

for future human and parasite malaria molecular studies for a period of up to 10 years. In addition to drops of blood, all participants will also be asked about their age, date of birth, recent illnesses, including history of fever, occupation, travel history, residence, use of insecticide treated mosquito nets or antimalaria medication taking in the past 24/48 hours.

Risks, Discomfort and Inconvenience: Your child may feel a little pain or fear when their finger is pricked. The pain will dissipate within a few hours.

Benefits: There are no direct benefits for your child to participate in this study. However, the findings generated from the study will inform the National Malaria Control Programme in decision-making about the use of antimalarials and the best strategies to control and eliminate malaria from the country.

Costs of Participation/Compensation: You will not receive any money or compensation for your child to take part in this study.

Privacy: The data collected will be anonymous, however the data obtained in this study may be shared with collaborating partners: the National Malaria Control Programme, Malaria Consortium Mozambique, Manhiça Health Research Center, ISGlobal, Institute of Disease Modeling and the University of California, San Francisco, USA. In relation to the DNA sequences of the malaria parasite, or your child’s personal data, these will be archived in an online database that can be shared with other scientists and researchers when the data are sent to scientific publications to report the results of this study.

Confidentiality: The information collected will be kept confidential and only the study team will have access to individuals’ information. The results of the study will be published and made available so that other interested people can learn from our study, but confidential information will not be shared in any circumstance. Your child’s data will be completely anonymised.

Sharing of results: Results from this research will be shared on open access platforms online, in public data repositories or directly in scientific publications, in order to facilitate further collaboration, enhance trust in the findings and goodwill among researchers. We will specifically focus on data sharing among other African countries in the region which are engaging in similar approaches to the molecular surveillance of malaria.

Whom to Contact (Investigators and Ethics Committee): in case of any of these situations:

- If your questions, concerns or complaints are not being addressed by the research team;
- If you are unable to contact the research team;
- If you would like to speak with someone who is not part of the research team;
- If you have questions about your rights as a research participant;
- If you wish to obtain information or provide information about this research; or
- If you think that the study has caused harm.

Please return to the Health Unit and speak with the workers involved in the study or contact the study focal person, assigned by Malaria Consortium Mozambique, Neide Canana on telephone number: 860450563, or you can contact her at: Malaria Consortium Mozambique, Sita Av. Lucas Elias Kumato nr. 118, Bairro da Sommerschield – Maputo City, Mozambique, or you can also contact Manhiça Health Research Center, located at: Street 12, Bairro Cambeve in Município da Manhiça Maputo Province, Mozambique, or by telephone: 21810002. In case you are not satisfied with the responses provided, you may also contact the National Bioethics for Health Committee, Ministry of Health, Mozambique on the numbers: 824066350/844693186.

Ethics Committee approval of this study: This study was approved by the Manhiça Health Research Center Institutional Bioethics Health Committee and the National Bioethics for Health Committee.

PART IV

DECLARATION OF INFORMED CONSENT

Study Title: "A prospective surveillance study to detect antimalarial drug resistance, gene deletions of diagnostic relevance and genetic diversity of *Plasmodium falciparum* in Mozambique."

Declaration: I have read the information provided in this consent form, including the risks and possible benefits. All my questions about the research have been answered satisfactorily. I understand that my child is free to withdraw from the study at any time without repercussions or loss of benefits to which they are entitled.

I give my consent for my child/ward to participate in this study.

INFORMED CONSENT

If there is any part of this consent form that you do not understand, ask the investigator before you sign.

I, _____________________ (Name of father/mother/guardian) give my voluntary consent for my child or ward to participate in the study: "A prospective surveillance study to detect antimalarial drug resistance, gene deletions of diagnostic relevance and genetic diversity of *Plasmodium falciparum* in Mozambique."

My questions have all been answered by _________________ (Name of researcher) in my own language. In case I have any other questions, I know that I can contact the study focal person assigned to Malaria Consortium and the National Bioethics for Health Committee through the contacts provided. I understand that I may withdraw my child's participation from the study, at any time for any reason, without any repercussions.

Do you allow your child/ward's samples to be stored and used in future research? □ **Yes** □ **No**

I agree for my child/ward to take part in this study.

Signatures

______________________________ ______________
Signature of father/mother/guardian Date and time

Father/Mother/guardian fingerprint if they cannot sign

____
Name of father/mother/guardian (in capital letters)

____
Signature of the person who explained consent

_______________________________________ _______________
Name of the person who explained consent (in capital letters) Date and time

If the father/mother/guardian does not know how to read, an impartial witness must also sign this form:

____ ____________
Signature of the impartial witness Date and time

____
Name of the impartial witness (in capital letters)

Appendix 3. Information sheet and informed consent for adult pregnant women.

PART I

INFORMATION SHEET AND INFORMED CONSENT FOR ADULT PREGNANT WOMEN

Name of Affiliated Institutions

25. Manhiça Health Research Center (CISM), Manhiça, Mozambique
26. Malaria Consortium, Maputo, Mozambique
27. Barcelona Global Health Institute (ISGlobal), Barcelona, Spain
28. National Malaria Control Programme, Ministry of Health, Maputo, Mozambique
29. University of California, San Francisco, USA
30. Clinton Health Access Initiative, Boston, USA
31. Institute of Disease Modeling, Bill and Melinda Gates Foundation, Seattle, USA
32. Bill and Melinda Gates Foundation, Seattle, USA

Protocol title and version: "A prospective surveillance study to detect antimalarial drug resistance, gene deletions of diagnostic relevance and genetic diversity of *Plasmodium falciparum* in Mozambique," version number 7, 25 August 2021.

Name and affiliation of Principal Investigator(s): Baltazar Candrinho, National Malaria Control Programme, Ministry of Health, Maputo, Mozambique and Alfredo Mayor, Manhiça Health Research Center, Manhiça, Mozambique.

Study funder: Bill and Melinda Gates Foundation, USA

Introduction: The National Malaria Control Programme in partnership with Malaria Consortium and the Manhiça Health Research Center are conducting a study to analyse the genetics of malaria parasites to identify the best ways to control and/or eliminate this disease from the country.

Please read this form with care. This form provides important information about participating in this study. All the information which follows, discussed below, is to allow you to understand what the study involves and the steps that would need your collaboration, so that before becoming involved in the study, you can decide freely if you wish to participate.

You can take the time that you feel necessary to decide about your participation in this study. If you have questions about the study, or any part of this form, please ask us. If you decide to participate in this research, you will be asked to sign this form. One copy of the signed form will be provided to you for your records. If at any time you feel that you do not understand the information that is being provided, please do not hesitate to interrupt so that we can explain and clarify everything again.

After receiving your consent to participate, we will ask you some personal questions about your age, date of birth, recent illnesses, including history of fever, occupation, travel history, residence, use of insecticide treated mosquito nets or taking of antimalarial medications in the last month and then we will take a few drops of blood from your finger.

Rationale: Mozambique constitutes a main goal for the World Health Organization and partnership initiative, namely, Roll Back Malaria, to end malaria in the world. In this context, through involvement in regional malaria elimination initiatives, the use of molecular malaria surveillance data, as a complement to traditional surveillance information, can contribute to the elimination of malaria in Southern Mozambique and a reduction of the burden in the north of the country. However, there is a lack of malaria diagnostic and drug resistance data and other measures of the genetic diversity of the parasite that causes malaria in different transmission settings. Therefore, more evidence is needed to demonstrate the feasibility of using genetic data as a driver of the intensity of transmission in high transmission areas. Additionally, understanding the prevalence of

diagnostic and drug resistance and genetic diversity will inform more appropriate and impactful interventions to reduce malaria morbidity and mortality in Mozambique. The integration of genetic data into routine surveillance activities has the potential to increase knowledge for programmatic decision-making on the optimal combination of control and elimination measures in Mozambique.

Research objectives: Your participation in this study will help us to identify the prevalence of molecular markers of antimalarial resistance along with other genetic markers, which will inform the National Malaria Control Programme to support decision-making on the use of antimalarials and best strategies to control and eliminate malaria in the country.

Type of research/Intervention: This is prospective, operational surveillance research.

Selection of participants: You were invited to participate in this research because you are part of a group that is the focus of this study: **adult pregnant women** with malaria, confirmed by a rapid diagnostic test, living in this region.

Voluntary participation: It's your choice if you want to participate in this study or not. Refusing to participate or withdrawing your participation will not result in any penalty or loss of health benefits or services. You will continue to receive medical care if you choose not to participate in this study. Your decision will not change the care that you receive now or in the future. Participating in this study is your choice. If you decide to participate in this study, you can leave at any time without consequences. If you want to stop participating in the study, just let the research team know.

Procedures: We will take a few drops of blood from your finger and four drops will be placed on two small pieces of paper (filter paper), two drops on each paper. The filter papers containing four drops of blood each will be kept in the Health Unit and sent to Manhiça Health Research Center where the analysis will be done. If necessary, the filter papers may be sent to a laboratory located outside of Mozambique (specifically, the ISGlobal laboratory in Spain or the University of California, San Francisco laboratory, in the United States) for additional analysis and molecular characterisation of the malaria parasites (alleles related to antimalarial resistance as well as genetic composition and other molecular markers of relevance to malaria surveillance, both in the parasite and human host). The filter papers will be stored by the Manhiça Health Research Center for future human and parasite malaria molecular studies for a period of up to 10 years. In addition to drops of blood, all participants will also be asked about their age, date of birth, recent illnesses, including history of fever, occupation, travel history, residence, use of insecticide treated mosquito nets or antimalaria medication taking in the past 24/48 hours.

Risks, Discomfort and Inconvenience: You may feel a little pain or fear when your finger is pricked. The pain will dissipate within a few hours.

Benefits: There are no direct benefits for you to participate in this study. However, the findings generated from the study will inform the National Malaria Control Programme in decision-making about the use of antimalarials and the best strategies to control and eliminate malaria from the country.

Costs of Participation/Compensation: You will not receive any money or compensation to take part in this study.

Privacy: The data collected will be anonymous, however the data obtained in this study may be shared with collaborating partners: the National Malaria Control Programme, Malaria Consortium Mozambique, Manhiça Health Research Center, ISGlobal, Institute of Disease Modeling and the University of California, San Francisco, USA. In relation to the DNA sequences of the malaria parasite, or your personal data, these will be archived in an online database that can be shared with other scientists and researchers when the data are sent to scientific publications to report the results of this study.

Confidentiality: The information collected will be kept confidential and only the study team will have access to individuals' information. The results of the study will be published and made available so that other interested

people can learn from our study, but confidential information will not be shared in any circumstance. Your data will be completely anonymised.

Sharing of results: Results from this research will be shared on open access platforms online, in public data repositories or directly in scientific publications, in order to facilitate further collaboration, enhance trust in the findings and goodwill among researchers. We will specifically focus on data sharing among other African countries in the region which are engaging in similar approaches to the molecular surveillance of malaria.

Whom to Contact (Investigators and Ethics Committee): in case of any of these situations:

- If your questions, concerns or complaints are not being addressed by the research team;
- If you are unable to contact the research team;
- If you would like to speak with someone who is not part of the research team;
- If you have questions about your rights as a research participant;
- If you wish to obtain information or provide information about this research; or
- If you think that the study has caused harm.

Please return to the Health Unit and speak with the workers involved in the study or contact the study focal person, assigned by Malaria Consortium Mozambique, Neide Canana on telephone number: 860450563, or you can contact her at: Malaria Consortium Mozambique, Sita Av. Lucas Elias Kumato nr. 118, Bairro da Sommerschield – Maputo City, Mozambique, or you can also contact Manhiça Health Research Center, located at: Street 12, Bairro Cambeve in Município da Manhiça Maputo Province, Mozambique, or by telephone: 21810002. In case you are not satisfied with the responses provided, you may also contact the National Bioethics for Health Committee, Ministry of Health, Mozambique on the numbers: 824066350/844693186.

Ethics Committee approval of this study: This study was approved by the Manhiça Health Research Center Institutional Bioethics Health Committee and the National Bioethics for Health Committee.

PART II

DECLARATION OF INFORMED CONSENT

Study Title: “A prospective surveillance study to detect antimalarial drug resistance, gene deletions of diagnostic relevance and genetic diversity of *Plasmodium falciparum* in Mozambique.”

Declaration: I have read the information provided in this consent form, including the risks and possible benefits. All my questions about the research have been answered satisfactorily. I understand that I am free to withdraw from the study at any time without repercussions or loss of benefits to which I am entitled.

I give my consent to participate in this study.

INFORMED CONSENT

If there is any part of this consent form that you do not understand, ask the investigator before you sign.

I, _____________________ (Name of participant) give my voluntary consent to participate in the study: “A prospective surveillance study to detect antimalarial drug resistance, gene deletions of diagnostic relevance and genetic diversity of *Plasmodium falciparum* in Mozambique.”

My questions have all been answered by _________________ (Name of researcher) in my own language. In case I have any other questions, I know that I can contact the study focal person assigned to Malaria Consortium and the National Bioethics for Health Committee through the contacts provided. I understand that I may withdraw my participation from the study, at any time for any reason, without any repercussions.

Do you allow your samples to be stored and used in future research? □ **Yes** □ **No**

I agree to take part in this study.

Signatures

Participant's fingerprint if they cannot sign

______________________________ _____________
Signature of participant Date and time

____
Name of participant (in capital letters)

____
Signature of the person who explained consent

___________________________________ _______________
Name of the person who explained consent (in capital letters) Date and time

If the participant does not know how to read, an impartial witness must also sign this form:

____ ____________
Signature of the impartial witness Date and time

____
Name of the impartial witness (in capital letters)

Mayor A, *et al. BMJ Open* 2022; 12:e063456. doi: 10.1136/bmjopen-2022-063456

Appendix 4. Information sheet and informed assent for pregnant women between 12 and 18 years of age.

PART I

INFORMATION SHEET AND INFORMED ASSENT FOR PREGNANT WOMEN BETWEEN 12 AND 18 YEARS OF AGE

Name of Affiliated Institutions

33. Manhiça Health Research Center (CISM), Manhiça, Mozambique
34. Malaria Consortium, Maputo, Mozambique
35. Barcelona Global Health Institute (ISGlobal), Barcelona, Spain
36. National Malaria Control Programme, Ministry of Health, Maputo, Mozambique
37. University of California, San Francisco, USA
38. Clinton Health Access Initiative, Boston, USA
39. Institute of Disease Modeling, Bill and Melinda Gates Foundation, Seattle, USA
40. Bill and Melinda Gates Foundation, Seattle, USA

Protocol title and version: "A prospective surveillance study to detect antimalarial drug resistance, gene deletions of diagnostic relevance and genetic diversity of *Plasmodium falciparum* in Mozambique," version number 7, 25 August 2021.

Name and affiliation of Principal Investigator(s): Baltazar Candrinho, National Malaria Control Programme, Ministry of Health, Maputo, Mozambique and Alfredo Mayor, Manhiça Health Research Center, Manhiça, Mozambique.

Study funder: Bill and Melinda Gates Foundation, USA

Introduction: The National Malaria Control Programme in partnership with Malaria Consortium and the Manhiça Health Research Center are conducting a study to analyse the genetics of malaria parasites to identify the best ways to control and/or eliminate this disease from the country.

Please read this form with care. This form provides important information about participating in this study. All the information which follows, discussed below, is to allow you to understand what the study involves and the steps that would need your collaboration, so that before becoming involved in the study, you can decide freely if you wish to participate.

You can take the time that you feel necessary to decide about your participation in this study. If you have questions about the study, or any part of this form, please ask us. If you decide to participate in this research, you will be asked to sign this form. One copy of the signed form will be provided to you for your records. If at any time you feel that you do not understand the information that is being provided, please do not hesitate to interrupt so that we can explain and clarify everything again.

After receiving your consent to participate, we will ask you some personal questions about your age, date of birth, recent illnesses, including history of fever, occupation, travel history, residence, use of insecticide treated mosquito nets or taking of antimalarial medications in the last month and then we will take a few drops of blood from your finger.

Rationale: Mozambique constitutes a main goal for the World Health Organization and partnership initiative, namely, Roll Back Malaria, to end malaria in the world. In this context, through involvement in regional malaria elimination initiatives, the use of molecular malaria surveillance data, as a complement to traditional surveillance information, can contribute to the elimination of malaria in Southern Mozambique and a reduction of the burden in the north of the country. However, there is a lack of malaria diagnostic and drug resistance data and other measures of the genetic diversity of the parasite that causes malaria in different transmission settings. Therefore, more evidence is needed to demonstrate the feasibility of using genetic data as a driver of the intensity of transmission in high transmission areas. Additionally, understanding the prevalence of

diagnostic and drug resistance and genetic diversity will inform more appropriate and impactful interventions to reduce malaria morbidity and mortality in Mozambique. The integration of genetic data into routine surveillance activities has the potential to increase knowledge for programmatic decision-making on the optimal combination of control and elimination measures in Mozambique.

Research objectives: Your participation in this study will help us to identify the prevalence of molecular markers of antimalarial resistance along with other genetic markers, which will inform the National Malaria Control Programme to support decision-making on the use of antimalarials and best strategies to control and eliminate malaria in the country.

Type of research/Intervention: This is prospective, operational surveillance research.

Selection of participants: You were invited to participate in this research because you are part of a group that is the focus of this study: **pregnant women between 12 to 18 years of age** with malaria, confirmed by a rapid diagnostic test, living in this region.

Voluntary participation: It's your choice if you want to participate in this study or not. Refusing to participate or withdrawing your participation will not result in any penalty or loss of health benefits or services. You will continue to receive medical care if you choose not to participate in this study. Your decision will not change the care that you receive now or in the future. Participating in this study is your choice. If you decide to participate in this study, you can leave at any time without consequences. If you want to stop participating in the study, just let the research team know.

Procedures: We will take a few drops of blood from your finger and four drops will be placed on two small pieces of paper (filter paper), two drops on each paper. The filter papers containing four drops of blood each will be kept in the Health Unit and sent to Manhiça Health Research Center where the analysis will be done. If necessary, the filter papers may be sent to a laboratory located outside of Mozambique (specifically, the ISGlobal laboratory in Spain or the University of California, San Francisco laboratory, in the United States) for additional analysis and molecular characterisation of the malaria parasites (alleles related to antimalarial resistance as well as genetic composition and other molecular markers of relevance to malaria surveillance, both in the parasite and human host). The filter papers will be stored by the Manhiça Health Research Center for future human and parasite malaria molecular studies for a period of up to 10 years. In addition to drops of blood, all participants will also be asked about their age, date of birth, recent illnesses, including history of fever, occupation, travel history, residence, use of insecticide treated mosquito nets or antimalaria medication taking in the past 24/48 hours.

Risks, Discomfort and Inconvenience: You may feel a little pain or fear when your finger is pricked. The pain will dissipate within a few hours.

Benefits: There are no direct benefits for you to participate in this study. However, the findings generated from the study will inform the National Malaria Control Programme in decision-making about the use of antimalarials and the best strategies to control and eliminate malaria from the country.

Costs of Participation/Compensation: You will not receive any money or compensation to take part in this study.

Privacy: The data collected will be anonymous, however the data obtained in this study may be shared with collaborating partners: the National Malaria Control Programme, Malaria Consortium Mozambique, Manhiça Health Research Center, ISGlobal, Institute of Disease Modeling and the University of California, San Francisco, USA. In relation to the DNA sequences of the malaria parasite, or your personal data, these will be archived in an online database that can be shared with other scientists and researchers when the data are sent to scientific publications to report the results of this study.

Confidentiality: The information collected will be kept confidential and only the study team will have access to individuals' information. The results of the study will be published and made available so that other interested

people can learn from our study, but confidential information will not be shared in any circumstance. Your data will be completely anonymised.

Sharing of results: Results from this research will be shared on open access platforms online, in public data repositories or directly in scientific publications, in order to facilitate further collaboration, enhance trust in the findings and goodwill among researchers. We will specifically focus on data sharing among other African countries in the region which are engaging in similar approaches to the molecular surveillance of malaria.

Whom to Contact (Investigators and Ethics Committee): in case of any of these situations:

- If your questions, concerns or complaints are not being addressed by the research team;
- If you are unable to contact the research team;
- If you would like to speak with someone who is not part of the research team;
- If you have questions about your rights as a research participant;
- If you wish to obtain information or provide information about this research; or
- If you think that the study has caused harm.

Please return to the Health Unit and speak with the workers involved in the study or contact the study focal person, assigned by Malaria Consortium Mozambique, Neide Canana on telephone number: 860450563, or you can contact her at: Malaria Consortium Mozambique, Sita Av. Lucas Elias Kumato nr. 118, Bairro da Sommerschield – Maputo City, Mozambique, or you can also contact Manhiça Health Research Center, located at: Street 12, Bairro Cambeve in Município da Manhiça Maputo Province, Mozambique, or by telephone: 21810002. In case you are not satisfied with the responses provided, you may also contact the National Bioethics for Health Committee, Ministry of Health, Mozambique on the numbers: 824066350/844693186.

Ethics Committee approval of this study: This study was approved by the Manhiça Health Research Center Institutional Bioethics Health Committee and the National Bioethics for Health Committee.

PART II

DECLARATION OF ASSENT

Study Title: “A prospective surveillance study to detect antimalarial drug resistance, gene deletions of diagnostic relevance and genetic diversity of *Plasmodium falciparum* in Mozambique.”

Declaration: I have read the information provided in this assent form, including the risks and possible benefits. All my questions about the research have been answered satisfactorily. I understand that I am free to withdraw from the study at any time without repercussions or loss of benefits to which I am entitled.

I give my assent to participate in this study.

INFORMED ASSENT

If there is any part of this assent form that you do not understand, ask the investigator before you sign.

I, _____________________ (Name of participant) give my voluntary assent to participate in the study: “A prospective surveillance study to detect antimalarial drug resistance, gene deletions of diagnostic relevance and genetic diversity of *Plasmodium falciparum* in Mozambique.”

My questions have all been answered by _________________ (Name of researcher) in my own language. In case I have any other questions, I know that I can contact the study focal person assigned to Malaria Consortium and the National Bioethics for Health Committee through the contacts provided. I understand that I may withdraw my participation from the study, at any time for any reason, without any repercussions.

Do you allow your samples to be stored and used in future research? □ **Yes** □ **No**

I agree to take part in this study.

Signatures

Minor's fingerprint if they cannot sign

______________________________ _____________
Signature of the minor Date and time

____
Minor's name (in capital letters)

____
Signature of the person who explained assent

_______________________________________ _______________
Name of the person who explained assent (in capital letters) Date and time

If the minor does not know how to read, an impartial witness must also sign this form:

____ ____________
Signature of the impartial witness Date and time

____
Name of the impartial witness (in capital letters)

PART III

INFORMATION SHEET AND INFORMED CONSENT FOR PARENTS/GUARDIANS OF PREGNANT WOMEN LESS THAN 18 YEARS OF AGE

Name of Affiliated Institutions

41. Manhiça Health Research Center (CISM), Manhiça, Mozambique
42. Malaria Consortium, Maputo, Mozambique
43. Barcelona Global Health Institute (ISGlobal), Barcelona, Spain
44. National Malaria Control Programme, Ministry of Health, Maputo, Mozambique
45. University of California, San Francisco, USA
46. Clinton Health Access Initiative, Boston, USA
47. Institute of Disease Modeling, Bill and Melinda Gates Foundation, Seattle, USA
48. Bill and Melinda Gates Foundation, Seattle, USA

Protocol title and version: "A prospective surveillance study to detect antimalarial drug resistance, gene deletions of diagnostic relevance and genetic diversity of *Plasmodium falciparum* in Mozambique," version number 7, 25 August 2021.

Name and affiliation of Principal Investigator(s): Baltazar Candrinho, National Malaria Control Programme, Ministry of Health, Maputo, Mozambique and Alfredo Mayor, Manhiça Health Research Center, Manhiça, Mozambique.

Study funder: Bill and Melinda Gates Foundation, USA

Introduction: The National Malaria Control Programme in partnership with Malaria Consortium and the Manhiça Health Research Center are conducting a study to analyse the genetics of malaria parasites to identify the best ways to control and/or eliminate this disease from the country.

Please read this form with care. This form provides important information about participating in this study. All the information which follows, discussed below, is to allow you to understand what the study involves and the steps that would need your collaboration, so that before becoming involved in the study, you can decide freely if you wish to participate.

You can take the time that you feel necessary to decide about your participation in this study. If you have questions about the study, or any part of this form, please ask us. If you decide for your child/ward to participate in this research, you will be asked to sign this form. One copy of the signed form will be provided to you for your records. If at any time you feel that you do not understand the information that is being provided, please do not hesitate to interrupt so that we can explain and clarify everything again.

After receiving your consent for your child/ward to participate, we will ask them some personal questions about their age, date of birth, recent illnesses, including history of fever, occupation, travel history, residence, use of insecticide treated mosquito nets or taking of antimalarial medications in the last month and then we will take a few drops of blood from their finger.

Rationale: Mozambique constitutes a main goal for the World Health Organization and partnership initiative, namely, Roll Back Malaria, to end malaria in the world. In this context, through involvement in regional malaria elimination initiatives, the use of molecular malaria surveillance data, as a complement to traditional surveillance information, can contribute to the elimination of malaria in Southern Mozambique and a reduction of the burden in the north of the country. However, there is a lack of malaria diagnostic and drug resistance data and other measures of the genetic diversity of the parasite that causes malaria in different transmission settings. Therefore, more evidence is needed to demonstrate the feasibility of using genetic data as a driver of the intensity of transmission in high transmission areas. Additionally, understanding the prevalence of diagnostic and drug resistance and genetic diversity will inform more appropriate and impactful interventions to reduce malaria morbidity and mortality in Mozambique. The integration of genetic data into routine surveillance activities has the potential to increase knowledge for programmatic decision-making on the optimal combination of control and elimination measures in Mozambique.

Research objectives: Your child/ward's participation in this study will help us to identify the prevalence of molecular markers of antimalarial resistance along with other genetic markers, which will inform the National Malaria Control Programme to support decision-making on the use of antimalarials and best strategies to control and eliminate malaria in the country.

Type of research/Intervention: This is prospective, operational surveillance research.

Selection of participants: Your child/ward was invited to participate in this research because they are part of a group that is the focus of this study: **pregnant women between 12 to 18 years of age** with malaria, confirmed by a rapid diagnostic test, living in this region.

Voluntary participation: It's your choice if you want your child/ward to participate in this study or not. Refusing to participate or withdrawing their participation will not result in any penalty or loss of health benefits or services. Your child/ward will continue to receive medical care if you/they choose not to participate in this study. Your decision will not change the care that they receive now or in the future. Participating in this study is your/their choice. If you decide for them to participate in this study, they can leave at any time without consequences. If they want to stop participating in the study, just let the research team know.

Procedures: We will take a few drops of blood from your child/ward's finger and four drops will be placed on two small pieces of paper (filter paper), two drops on each paper. The filter papers containing four drops of blood each will be kept in the Health Unit and sent to Manhiça Health Research Center where the analysis will be done. If necessary, the filter papers may be sent to a laboratory located outside of Mozambique (specifically, the ISGlobal laboratory in Spain or the University of California, San Francisco laboratory, in the United States) for additional analysis and molecular characterisation of the malaria parasites (alleles related to antimalarial resistance as well as genetic composition and other molecular markers of relevance to malaria surveillance,

both in the parasite and human host). The filter papers will be stored by the Manhiça Health Research Center for future human and parasite malaria molecular studies for a period of up to 10 years. In addition to drops of blood, all participants will also be asked about their age, date of birth, recent illnesses, including history of fever, occupation, travel history, residence, use of insecticide treated mosquito nets or antimalaria medication taking in the past 24/48 hours.

Risks, Discomfort and Inconvenience: Your child/ward may feel a little pain or fear when their finger is pricked. The pain will dissipate within a few hours.

Benefits: There are no direct benefits for you to participate in this study. However, the findings generated from the study will inform the National Malaria Control Programme in decision-making about the use of antimalarials and the best strategies to control and eliminate malaria from the country.

Costs of Participation/Compensation: You will not receive any money or compensation for your child/ward to take part in this study.

Privacy: The data collected will be anonymous, however the data obtained in this study may be shared with collaborating partners: the National Malaria Control Programme, Malaria Consortium Mozambique, Manhiça Health Research Center, ISGlobal, Institute of Disease Modeling and the University of California, San Francisco, USA. In relation to the DNA sequences of the malaria parasite, or your child/ward's personal data, these will be archived in an online database that can be shared with other scientists and researchers when the data are sent to scientific publications to report the results of this study.

Confidentiality: The information collected will be kept confidential and only the study team will have access to individuals' information. The results of the study will be published and made available so that other interested people can learn from our study, but confidential information will not be shared in any circumstance. Your child/ward's data will be completely anonymised.

Sharing of results: Results from this research will be shared on open access platforms online, in public data repositories or directly in scientific publications, in order to facilitate further collaboration, enhance trust in the findings and goodwill among researchers. We will specifically focus on data sharing among other African countries in the region which are engaging in similar approaches to the molecular surveillance of malaria.

Whom to Contact (Investigators and Ethics Committee): in case of any of these situations:

- If your questions, concerns or complaints are not being addressed by the research team;
- If you are unable to contact the research team;
- If you would like to speak with someone who is not part of the research team;
- If you have questions about your rights as a research participant;
- If you wish to obtain information or provide information about this research; or
- If you think that the study has caused harm.

Please return to the Health Unit and speak with the workers involved in the study or contact the study focal person, assigned by Malaria Consortium Mozambique, Neide Canana on telephone number: 860450563, or you can contact her at: Malaria Consortium Mozambique, Sita Av. Lucas Elias Kumato nr. 118, Bairro da Sommerschield – Maputo City, Mozambique, or you can also contact Manhiça Health Research Center, located at: Street 12, Bairro Cambeve in Município da Manhiça Maputo Province, Mozambique, or by telephone: 21810002. In case you are not satisfied with the responses provided, you may also contact the National Bioethics for Health Committee, Ministry of Health, Mozambique on the numbers: 824066350/844693186.

Ethics Committee approval of this study: This study was approved by the Manhiça Health Research Center Institutional Bioethics Health Committee and the National Bioethics for Health Committee.

PART IV

DECLARATION OF INFORMED CONSENT

Study Title: "A prospective surveillance study to detect antimalarial drug resistance, gene deletions of diagnostic relevance and genetic diversity of *Plasmodium falciparum* in Mozambique."

Declaration: I have read the information provided in this consent form, including the risks and possible benefits. All my questions about the research have been answered satisfactorily. I understand that my child/ward is free to withdraw from the study at any time without repercussions or loss of benefits to which I am entitled.

I give my consent for my child/ward to participate in this study.

INFORMED CONSENT

If there is any part of this consent form that you do not understand, ask the investigator before you sign.

I, ______________________ (Name of father/mother/guardian) give my voluntary consent for my child or ward to participate in the study: "A prospective surveillance study to detect antimalarial drug resistance, gene deletions of diagnostic relevance and genetic diversity of *Plasmodium falciparum* in Mozambique."

My questions have all been answered by __________________ (Name of researcher) in my own language. In case I have any other questions, I know that I can contact the study focal person assigned to Malaria Consortium and the National Bioethics for Health Committee through the contacts provided. I understand that my child/ward may withdraw their participation from the study, at any time for any reason, without any repercussions.

Do you allow your child/ward's samples to be stored and used in future research? □ **Yes** □ **No**

I agree for my child/ward to take part in this study.

Signatures

________________________________ ______________
Signature of father/mother/guardian Date and time

Father/mother/guardian fingerprint if they cannot sign

____
Name of father/mother/guardian (in capital letters)

____
Signature of the person who explained consent

____ ________________
Name of the person who explained consent (in capital letters) Date and time

If the participant/legal representative does not know how to read, an impartial witness must also sign this form:

____ _____________
Signature of the impartial witness Date and time

____
Name of the impartial witness (in capital letters)

Appendix 5. Questionnaire for medium-high transmission area, children under 2-10 years old.

	**Study site information**	
1.	Date of sample collection (dd/mm/yy)	|__|__||__|__||__|__|
2.	Province of residence	[ ]
3.	District of residence	[ ]
4.	Administrative post	[ ]
5.	Place of residence	[ ]
6.	Health Unit (name or code)	[ ]
7.	Referred by APE in the community	Yes ☐ No ☐
	**Inclusion criteria**	
8.	Was the informed consent form signed?<br>***If no, end the survey.***	Yes ☐ No ☐
9.	History of fever/hot body in the last 24 hours?	Yes ☐ No ☐
10.	Axillary temperature at the time of the survey<br>***If temperature is <37.5ºC, end the survey.***	|__|__|.|__| ºC
11.	Date of birth (dd/mm/yyyy)<br><br>11.1. Age (years)	|__|__|/|__|__|/|__|__|__|__| <br>|__|__|
12.	Does the participant have severe malaria?<br>**If yes, end the survey.**	Yes ☐ No ☐
13.	Does the participant reside in the study area (district)?<br>***If no, end the survey.***	Yes ☐ No ☐
14.	Has the child taken antimalarials in the past 14 days? (check yellow health card)<br>***If yes, end the survey.***	Yes ☐ No ☐
15.	Was a routine RDT performed?<br>***If no, end the survey.***<br>15.1 *If yes, the result was:*<br><br>***If negative, end the survey.***	Yes ☐ No ☐<br><br>Positive ☐ Negative ☐ Inconclusive ☐
16.	Was an additional RDT performed?	Yes ☐ No ☐ Not applicable ☐<br>**If 'not applicable', skip to question 17.**
	16.1. Result of line T1 (HRP2)	Positive ☐ Negative ☐ Inconclusive ☐
	16.2. Result of line T2 (LDH)	Positive ☐ Negative ☐ Inconclusive ☐
	**Participant information**	
17.	Sex	Male ☐ Female ☐
18.	Study ID number<br><br>*US|__|__|-|__|__|-|__|__|__|*	19. Sample ID number — *Insert bar code*<br><br>**Now put the sample ID number on the informed consent form.**
	**Travel information**	
20.	Have you travelled in the past 28 days?	Yes ☐ No ☐

If not, go to question 21.	
20.1 When did you start your trip? (date: dd/mm)	|__|__||__|__|
20.2 If yes, for how many nights?	
20.3 Where did you travel?:	|__|__|
Country	[__________________]
Province	[__________________]
District	[__________________]
20.4 During the trip, did you sleep under a mosquito net?	Yes ☐ No ☐
**Information related to malaria**	
21. How many times has the child had episodes of fever in the past month?	|__|__|
22. Did the child sleep under a mosquito net last night??	Yes ☐ No ☐
22.1 *If yes, was it an insecticide treated net?*	Yes ☐ No ☐
23. Has there been indoor residual spraying in the past 6 months?	Yes ☐ No ☐
24. Has the child taken antimalarial medications in the past month?	Yes ☐ No ☐
25. Is the child taking cotrimoxazole?	Yes ☐ No ☐ Don`t know ☐
Now label the two filter papers (sample ID number)	
26. Was a blood sample collected on the filter paper?	Yes ☐ No ☐
27. If yes, state the number of papers	2 ☐ Other |__|

**Interviewer information**	
28. Interviewer number	|__|__|__|__|
29. Interviewer initials	|__|__|__|__|
30. Date of interview	(dd/mm/yyyy) |__|__|/|__|__|/|__|__|__|__|

Appendix 6. Questionnaire for low transmission area, all ages.

	**Study site information**	
1.	Date of sample collection (dd/mm/yy)	|__|__||__|__||__|__|
2.	Province of residence	[______________]
3.	District of residence	[______________]
4.	Administrative post	[______________]
5.	Place of residence	[______________]
6.	Health Unit (name or code)	[______________]
7.	Referred by APE in the community	Yes ☐ No ☐
	**Inclusion criteria**	
8.	Was the informed consent form signed?<br>***If no, end the survey.***	Yes ☐ No ☐
9.	History of fever/hot body in the last 24 hours?	Yes ☐ No ☐
10.	Axillary temperature at the time of the survey<br>***If temperature is <37.5ºC, end the survey.***	|__|__|.|__| ºC
11.	Date of birth (dd/mm/yyyy)<br><br>11.1. Age (years)	|__|__|/|__|__|/|__|__|__|__| <br>|__|__|
12.	Does the participant have severe malaria?<br>***If yes, end the survey.***	Yes ☐ No ☐
13.	Does the participant reside in the study area (district)?<br>***If no, end the survey.***	Yes ☐ No ☐
14.	Has the child/adult taken antimalarials in the past 14 days? (check yellow health card)<br>***If yes, end the survey.***	Yes ☐ No ☐
15.	Was a routine RDT performed?<br>***If no, end the survey.***<br>15.1 *If yes, the result was:*<br><br>***If negative, end the survey.***	Yes ☐ No ☐<br><br>Positive ☐ Negative ☐ Inconclusive ☐
16.	Was an additional RDT performed?	Yes ☐ No ☐ Not applicable ☐<br>**If 'not applicable', skip to question 17.**
	16.1. Result of line T1 (HRP2)	Positive ☐ Negative ☐ Inconclusive ☐
	16.2. Result of line T2 (LDH)	Positive ☐ Negative ☐ Inconclusive ☐
	**Participant information**	
17.	Sex	Male ☐ Female ☐
18.	Occupation	[______________]
19.	Study ID number<br><br>US|__|__|-|__|__|-|__|__|__|	**Sample ID number** *Insert bar code*<br><br>**Now put the sample ID number on the informed consent form.**

Mayor A, *et al. BMJ Open* 2022; 12:e063456. doi: 10.1136/bmjopen-2022-063456

**Travel information**	
20. Have you travelled in the past 28 days?	Yes ☐ No ☐
If not, go to question 21.	
20.1 When did you start your trip? (date: dd/mm)	|__|__||__|__|
20.2 If yes, for how many nights?	|__|__|
20.3 Where did you travel?:	
Country	[____________]
Province	[____________]
District	[____________]
20.4 During the trip, did you sleep under a mosquito net?	Yes ☐ No ☐
**Information related to malaria**	
21. How many times has the child/adult had episodes of fever in the past month?	|__|__|
22. Did the child/adult sleep under a mosquito net last night?	Yes ☐ No ☐
22.1 *If yes, was it an insecticide treated net?*	Yes ☐ No ☐
23. Has there been indoor residual spraying in the past 6 months?	Yes ☐ No ☐
24. Has the child/adult taken antimalarial medications in the past month?	Yes ☐ No ☐
25. Is the child/adult taking cotrimoxazole?	Yes ☐ No ☐ Don`t know ☐
Now label the two filter papers (sample ID number)	
26. Was a blood sample collected on the filter paper?	Yes ☐ No ☐
27. If yes, state the number of papers	2 ☐ Other |__|
**Interviewer information**	
28. Interviewer number	|__|__|__|__|
29. Interviewer initials	|__|__|__|__|
30. Date of interview	(dd/mm/yyyy) |__|__|/|__|__|/|__|__|__|__|

Appendix 7. Questionnaire for Pregnant women attending ANC clinic in medium-high transmission area.

	**Study site information**	
1.	Date of sample collection (dd/mm/yy)	|__|__||__|__||__|__|
2.	Province of residence	[ ]
3.	District of residence	[ ]
4.	Administrative post	[ ]
5.	Place of residence	[ ]
6.	Health Unit (name or code)	[ ]
7.	Referred by APE in the community	Yes ☐ No ☐
	**Inclusion criteria**	
8.	Was the informed consent form signed?<br>***If no, end the survey.***	Yes ☐ No ☐
9.	Is the participant pregnant?<br>***If no, end the survey.***	Yes ☐ No ☐
10.	Is this your first prenatal consult?<br>***If no, end the survey.***	Yes ☐ No ☐
11.	Date of birth (dd/mm/yyyy)	I__|__|/|__|__|/|__|__|__|__|
	11.1. Age (years)<br>***If aged <12 years, end the survey.***	|__|__|
12.	Does the participant reside in the study area (district)?<br>***If no, end the survey.***	Yes ☐ No ☐
13.	Does the participant have severe malaria?<br>***If yes, end the survey.***	Yes ☐ No ☐
	**Participant information**	
14.	Occupation	[ ]
15.	Study ID number<br>**PN**|__|__|-|__|__|-|__|__|__|	Sample ID number: *Insert bar code*

Now put the sample ID number on the informed consent form.

	**Participant characteristics**	
16.	History of fever/hot body in the last 24 hours?	Yes ☐ No ☐
17.	Axillary temperature at the time of the survey	|__|__|.|__| ºC
18.	Was an HIV test performed during this visit? (*check HIV card or proof of testing)*	Yes ☐ No ☐
	18.1. *If yes, HIV test result at this visit*	Positive ☐ Negative ☐ Inconclusive ☐
	18.2. *If no, state previous HIV test result (check HIV card or proof of testing)*	Negative ☐ Positive ☐
19.	Are you receiving ART? *(check in the woman's personal health record)*	Yes ☐ No ☐
20.	Are you taking cotrimoxazole? *(check in the woman's book)*	Yes ☐ No ☐
21.	Current haemoglobin result *(Hemocue test result from today)*	|__|__|, |__| g/dL
22.	Was a malaria RDT performed?	Yes ☐ No ☐

23.	*If yes, the result:*	Positive ☐ Negative ☐ Inconclusive ☐
24.	How many weeks pregnant are you currently?	|__|__|
25.	Method used to determine gestational age:<br>a) Last menstrual period ☐<br>b) Fundal height ☐<br>c) Other (specify) [____________]	
26.	How many previous pregnancies has the participant had before this one?	|__|__|
27.	Has the participant moved from the area during the last pregnancy?	Yes ☐ No ☐
	**Travel information**	
28.	Have you travelled during this pregnancy and spent the night away from home?	Yes ☐ No ☐
	28.1 When did you start your trip? (date: dd/mm)	|__|__||__|__|
	28.2 If yes, for how many nights?	|__|__|
	28.3 Where did you travel?:<br>Country<br>Province<br>District	[____________]<br>[____________]<br>[____________]
	28.4 During the trip, did you sleep under a mosquito net?	Yes ☐ No ☐
	**Information related to malaria**	
29.	How many times have you had episodes of fever in the past month?	|__|__|
30.	Did you sleep under a mosquito net last night?	Yes ☐ No ☐
30.1	*If yes, was it an insecticide treated net?*	Yes ☐ No ☐
31.	Has there been indoor residual spraying in the past 6 months?	Yes ☐ No ☐
32.	Has the participant received intermittent preventive treatment (IPT) before this visit for this pregnancy?	Yes ☐ No ☐
33.	Has the participant taken antimalarial medications in the past month?	Yes ☐ No ☐ Don`t know ☐
	**Now label the filter paper with the sample ID number**	
34.	Was a blood sample collected on the filter paper?	Yes ☐ No ☐
35.	If yes, state the number of papers	2 ☐ Other |__|

	**Interviewer information**	
36.	Interviewer number	|__|__|__|__|
37.	Interviewer initials	|__|__|__|__|
38.	Date of interview	(dd/mm/yyyy) |__|__|/|__|__|/|__|__|__|__|

Appendix 8. Questionnaire for Pregnant women attending ANC clinic in low transmission area.

	**Study site information**	
1	Date of sample collection (dd/mm/yy)	|__|__||__|__||__|__|
2.	Province of residence	[ ]
3.	District of residence	[ ]
4.	Administrative post	[ ]
5.	Place of residence	[ ]
6.	Neighbourhood of residence	[ ]
7.	Mobile phone number	[ ]
8.	Health Unit (name or code)	[ ]
9.	Referred by APE in the community	Yes ☐ No ☐
	**Inclusion criteria**	
10.	Was the informed consent form signed?<br>***If no, end the survey.***	Yes ☐ No ☐
11.	Is the participant pregnant?<br>***If no, end the survey.***	Yes ☐ No ☐
12.	Is this your first prenatal consult?<br>***If no, end the survey.***	Yes ☐ No ☐
13.	Date of birth (dd/mm/yyyy)<br>I__|__|/|__|__|/|__|__|__|__| <br>11.1. Age (years)<br>|__|__| ***If aged <12 years, end the survey.***	
14.	Does the participant reside in the study area (district)?<br>***If no, end the survey.***	Yes ☐ No ☐
15.	Does the participant have severe malaria?<br>***If yes, end the survey.***	Yes ☐ No ☐
	**Participant information**	
16.	Participant name	[ ]
17.	Occupation	[ ]
18.	Study number<br><br>**PN**|__|__|-|__|__|-|__|__|__|	Sample ID number: *Insert bar code*

Now put the sample ID number on the informed consent form.

	**Participant characteristics**	
19.	History of fever/hot body in the last 24 hours?	Yes ☐ No ☐
20.	Axillary temperature at the time of the survey	|__|__|.|__| ºC
21.	Was an HIV test performed during this visit? (*check HIV card or proof of testing)*	Yes ☐ No ☐
	18.1. *If yes, HIV test result at this visit*	Positive ☐ Negative ☐ Inconclusive ☐
	18.2. *If no, state previous HIV test result (check HIV card or proof of testing)*	Negative ☐ Positive ☐

22.	Are you receiving ART? *(check in the woman's personal health record)*	Yes ☐ No ☐
23.	Are you taking cotrimoxazole? *(check in the woman's book)*	Yes ☐ No ☐
24.	Current haemoglobin result *(Hemocue test result from today)*	|__|__|, |__| g/dL
25.	Was a malaria RDT performed?	Yes ☐ No ☐
26.	*If yes, the result:*	Positive ☐ Negative ☐ Inconclusive ☐
27.	How many weeks pregnant are you currently?	|__|__|
28.	Method used to determine gestational age:<br><br>a) Last menstrual period ☐<br>b) Fundal height ☐<br>c) Other (specify) [____________________]	
29.	How many previous pregnancies has the participant had before this one?	|__|__|
30.	Has the participant moved from the area during the last pregnancy?	Yes ☐ No ☐
	**Travel information**	
31.	Have you travelled during this pregnancy and spent the night away from home?	Yes ☐ No ☐
	31.1 When did you start your trip? (date: dd/mm)	|__|__||__|__|
	31.2 If yes, for how many nights?	
	31.3 Where did you travel?:	|__|__|
	Country	[____________________]
	Province	[____________________]
	District	[____________________]
	28.4 During the trip, did you sleep under a mosquito net?	Yes ☐ No ☐
	**Information related to malaria**	
32.	How many times have you had episodes of fever in the past month?	|__|__|
33.	Did you sleep under a mosquito net last night?	Yes ☐ No ☐
33.1	*If yes, was it an insecticide treated net?*	Yes ☐ No ☐
34.	Has there been indoor residual spraying in the past 6 months?	Yes ☐ No ☐
35.	Has the participant received intermittent preventive treatment (IPT) before this visit for this pregnancy?	Yes ☐ No ☐
36.	Has the participant taken antimalarial medications in the past month?	Yes ☐ No ☐ Don`t know ☐
	**Now label the filter paper with the sample ID number**	
37.	Was a blood sample collected on the filter paper?	Yes ☐ No ☐
38.	If yes, state the number of papers	2 ☐ Other |__|
	**Interviewer information**	
39.	Interviewer number	|__|__|__|__|
40.	Interviewer initials	|__|__|__|__|
41.	Date of interview	(dd/mm/yyyy) |__|__|/|__|__|/|__|__|__|__|

Appendix 9. Procedures for the collection, handling and storage of dried blood samples on filter paper and rapid diagnostic tests.

1 OBJECTIVES

To describe the correct collection, handling and storage procedures for dried blood samples on filter paper and rapid diagnostic tests (RDTs).

2 DEFINITIONS

- Filter paper: semipermeable paper used as a laboratory tool to collect and store blood samples for further molecular analysis. The filter paper code that will be used is Whatman Grade CF12 cut to 76x30mm (equal to the size of a microscope slide).
- **Rapid diagnostic test (RDT):** Lateral flow immunochromatographic tests. The RDTs for human malaria detect parasite specific antigens which are present in the blood of infected people. The most commonly used antigens are *Plasmodium falciparum Histidine-rich Protein 2* (PfHRP-2) and *Plasmodium Lactate Dehydrogenase* (pLDH).

3 APPLICABLE FOR

- All personnel responsible for the collection, handling and storage of dried blood samples on filter paper rand RDTs within the scope of malaria studies at CISM.

4 RESPONSIBILITIES

- **Investigators:** to guarantee that the SOPs are up to date and that technical personnel are properly trained and strictly follow the procedures described therein.
- **All technical personnel:** whether researcher, laboratory technicians, phlebotomists, physicians, or others who are engaged in field, clinical or laboratory activities involving filter papers or RDTs; all must know and strictly follow the content of these SOPs.

5 RELATED SOPS

- **POP_LB_012_PT**: Procedures for performing the malaria rapid diagnostic test (RDT)

6 SUPPLIES AND EQUIPMENT

Table 1. **Requisite supplies**

Type	Item
**Documents**	• Laboratory requisition form<br>• Health Unit sample record form<br>• Laboratory RDT sample placement form<br>• Laboratory filter paper placement form<br>• (Electronic document) Sample control in the laboratory
**Items for collection, transport and storage**	• Whatman Grade CF12 (ref. WHA10538018, slides 580x580mm) cut to slides sized 76x30mm<br>• Ziplock bags for individual samples (minimum length 80mm to the zip)<br>• Large Ziplock bags<br>• Lancets<br>• Silica gel<br>• Cotton balls<br>• Band aids<br>• Incinerator box<br>• Disposable gowns<br>• Freezer<br>• Refrigerator

	• Gloves (HI-CARE 2023-05)<br>• 8 sample identification numbers<br>• Alcohol (70%)<br>• Masks<br>• Sample transport cases<br>• Box for ground transport of samples
**Office supplies**	• Staples and staplers<br>• Markers (sharpie)<br>• Pens / Pencils<br>• 1 Printer<br>• Computer<br>• Toner<br>• Notebook<br>• Clipboard

7 PROCEDURE

7.1 Sample collection

7.1.1 Collection of blood sample on filter paper

- For each participant, four (4) circular capillary blood spots of approximately 50µl (equivalent to a diameter of 1.5-2cm) will be put on two Whatman CF12 filter papers, with two spots on each paper (**Figure 1**) in accordance with the following steps:
  1) Prepare 2 papers (76x30mm), alcohol (70%), cotton balls, sterile lancet.
  2) Clean the finger with a cotton ball soaked in 70% alcohol and wait for it to dry; it is recommended to use the middle or ring finger.
  3) Remove the protector part to release the sterile lancet.
  4) Firmly prick the side of the fingertip.
  5) Carefully squeeze and wipe the first drop of blood with a dry cotton ball.
  6) Let one or two drops of blood drip onto the filter paper for each blood spot in a diameter of approximately 1.5-2cm (**Figure 1**). It is important not to let the finger touch the filter paper, to avoid contamination; only a drop of blood may touch the paper.
  7) Wipe the fingertip with another cotton ball soaked in alcohol.

> NOTE: In case you collect a sample from a baby, and it is not possible to obtain two drops of blood from one of their fingers, alternatively, you can prick their heel. In this case, only one drop of blood on the filter paper is needed.

Figure 1. Placement of blood spots on the filter paper.

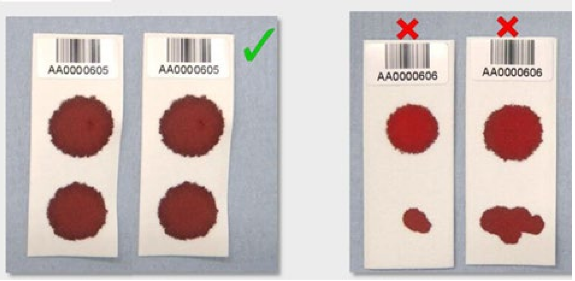

- Identify each sample with a sample identification number (the same sample identification number for each of the two filter papers) and include the collection date of the sample and the study acronym, using a pen.

33

- Put the same sample identification number on the **Laboratory analysis requisition form** (current version of POP_MAL_001_A01_PT) and fill out the form with the patient's details.
- Keep the remaining sample identification numbers stapled to each sample order for use at CISM.
- Record the sample collection data in the **Health Unit sample record form** (current version of POP_MAL_001_A02_PT).
- After blood collection, the filter paper must be dried at room temperature for 24 hours, in a safe, dry, cool and ventilated place (air conditioning can be used or the windows of the room can be opened, depending on the conditions of the site).
  - The drying surface, which can be a bench, cabinet or shelf, must be easy to clean and disinfect;
  - Avoid direct exposure to the sun or heat;
  - Do not allow samples from different patients to overlap, to avoid contamination;
  - When the process of drying is complete, the dried blood spots will be darker than the fresh blood spots.
- Once drying is complete, place the two filter papers from the same patient in a small Ziplock bag.
- Samples will be placed in a large Ziplock bag containing silica gel and stored in a refrigerator with a temperature between 2 to 8°C until the date of shipment to CISM, Manhiça district.
- Record the date that the samples are stored in the Health Unit refrigerator in the **Health Unit sample record form** (current version of POP_MAL_001_A02_PT).
- The respective requisitions must be kept in plastic files to be sent simultaneously with the samples.

7.1.2. RDT blood sample collection

- Blood collection for the RDT will be carried out following the **Procedures for performing the malaria rapid diagnostic test** (current version of POP_LB_PT_012_PT), also considering the manufacturer's specific instructions; do not discard the silica gel bag in the RDT package.
- Stick the sample identification number on the RDT and the same sample identification number on the **Laboratory analysis requisition form** (current version of POP_MAL_001_A01_PT). Write the collection date and study acronym on the RDT using a pen.
- Keep the RDT in an individual Ziplock and add the silica gel bag.
- The RDTs will be placed in a large Ziplock back and stored in a refrigerator between 2 to 8°C until the shipment date to CISM, Manhiça district.
- Record the storage of the samples on the **Health Unit sample record form** (current version of POP_MAL_001_A02_PT).
- Keep the remaining sample identification numbers stapled to each sample requisition for use at CISM.

7.2 Transport of filter papers to the CISM Laboratory

- Study personnel will contact the CISM study leader to prepare the shipment.
- Shipment logistics will be organised as follows:
  <u>For land transport:</u>
  - ambient temperature
  - the samples and documents will be placed in cases that must be exclusively used for this purpose (**Figure 2**)

  <u>For air transport:</u>
  - preferably using Portador Diário (https://www.portadordiario.co.mz/).
  - ambient temperature
  - the person responsible for the study will record the shipping code that will be assigned to the samples for later use at the time of collection at the final destination, as well as to monitor the location of the samples along the way
- At least one day before transport, verify the agreement between the actual number of samples and the records in the **Health Unit sample record form** (current version of POP_MAL_001_A02_PT).
- Whenever possible, the plastic boxes for transporting samples should be sanitised before and after use with soap and water, then disinfected with 70% alcohol.
- On the arranged day of transport, place samples (filter papers or RDTs), the requisition forms, the control forms and other study-specific documents in the shipping boxes.

Figure 2. Case for transporting samples (filter papers and RDTs) by land

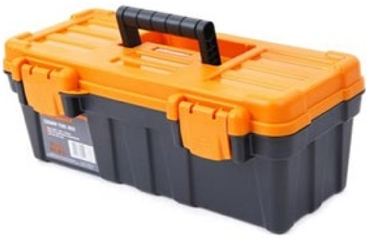

7.3 Receipt of samples at CISM

- Dried blood samples on filter paper for RDTs will be received at the CISM Laboratory, along with a laboratory requisition form.
- The Laboratory reception will verify the agreement between the sample identification numbers of the samples and the respective laboratory requisitions, and whether the number of samples received corresponds to the number of requisition forms.
- After verifying that everything is in order, the request will be entered into the SERVOLAB system, if not, the coordinator responsible must be informed so that they may follow up until the situation is resolved.

> NOTE: Samples without a laboratory requisition from partners will go through the laboratory reception for verification, however, these will not be entered into SERVOLAB due to insufficient data. The verification of these samples will be carried out together with the person responsible for the study, who must fill out the Excel Database **Control of samples in the laboratory** (current version of POP_MAL_001_A05_PT). This Excel document will be archived in electronic format and shared with the study team.

7.4 Storage of samples in the molecular biology laboratory

7.4.1. Storage of filter papers

- For storage, the two filter papers in each bag will be wrapped with aluminium foil and will be properly identified with the study name, bag number and group (A or B) using a permanent marker; the sample identification number will be stuck onto the aluminium foil;
- Place samples A in a large Ziplock bag (up to 100 filter papers) and samples B in a B Bag (up to 100 filter papers); then add 100g of silica gel to each bag (**Figure 3**).
- During the wrapping process, the **Filter paper placement form** (current version of POP_MAL_001_A03_PT) will be filled out simultaneously, which will then be verified by the technician responsible.
- The bags will be identified externally with the study name, bag group number (A or B) using a permanent marker and a paper containing the same information will be placed inside the bag.

Figure 3. Identification and storage of filter papers in the laboratory

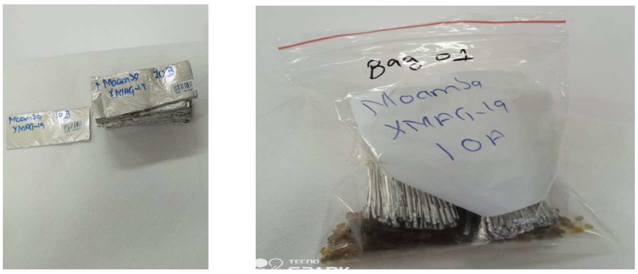

- Store A and B bags in a -20 degree freezer.

- The placement of the filter papers must be indicated in SERVOLAB (Seroteca Servolab>Type of Box 10x10->Box Name->filter paper bag X->placement).
- Lastly, fill out the Excel Database **Control of samples in the laboratory** (current version of POP_MAL_001_A05_PT).

7.4.2. Storage of RDTs

- For storage, the RDTs will be wrapped with aluminium foil and a sample identification number will be stuck onto the aluminium foil.
- During the wrapping process, the **RDT placement form** (current version of POP_MAL_001_A04_PT) will simultaneously be filled out, and then checked by the technician responsible.
- Place the samples from the same placement sheet in a bag (20 RDTs), then add 20g of silica gel.
- The bag will be identified externally with the study name, bag group number using a permanent marker and a paper containing the same information will be placed inside the bag.
- Place the bag of samples in a -20 degree freezer.
- The placement of the RDTs must be indicated on SERVOLAB (Seroteca Servolab>Type of Box 10x10->Box Name->RDT bag X->placement).
- Lastly, fill out the Excel Database **Control of samples in the laboratory** (current version of POP_MAL_001_A05_PT).

Mayor A, *et al. BMJ Open* 2022; 12:e063456. doi: 10.1136/bmjopen-2022-063456

Appendix 10. COVID-19 safety and research considerations.

1. COVID19-related biosafety capacities: This project will not involve the use of SARS-CoV-2 for any purpose, as we will focus on the detection of malaria molecular markers for surveillance and research purposes. The only samples that will be collected and managed in this project will be dried blood spots, obtained from individuals in the community and pregnant women at antenatal clinics, which will minimize the risk of COVID-19 infection among health workers and laboratory staff during sample collection and processing, respectively. All personnel involved in the study will be trained in the most up to date Malaria Consortium procedures for infection prevention and control. A daily monitoring of the health personnel involved will be conducted through the measurement of axillary temperature and identification of respiratory symptoms. In case of clinical signs, domiciliary isolation and COVID-19 testing will be recommended. CISM has developed a biosafety plan considering the following considerations:

Before starting any project-related activity, a new risk assessment will be completed using the template provided by WHO at their last version of the “Laboratory biosafety guidance related to coronavirus disease (COVID-19): interim guidance” (https://apps.who.int/iris/handle/10665/331500).

2. Collection of specimens: Finger or heel prick bloods will be collected from pregnant women at antenatal clinics and individuals in the community by community health workers. No nasopharyngeal nor oropharyngeal swabs will be collected. Samples will be collected following biosafety WHO guidelines (use of personal protective equipment [PPE]: N95 or KF94 mask, disposable gloves, protective clothing, eye protection and frequent hand washing) as described in WHO guidance on specimen collection, processing and laboratory testing: https://www.who.int/publicationsdetail/laboratory-testing-for-2019-novel-coronavirus-in-suspected-human-cases-20200117, and biosafety procedures https://apps.who.int/iris/bitstream/handle/10665/331138/WHO-WPE-GIH-2020.1-eng.pdf.

3. Laboratory biosafety: All biological samples for molecular assays will be managed at CISM. Given the nature of the samples (dried blood spot), there is a minimal risk of producing aerosols. In general, de-capping is considered a low-risk procedure. However, it depends on the design of the lid and container. Whether to proceed with the testing will be determined following a risk assessment, which considers the need for centrifugation, mixing, and aliquoting. In addition, the use of a BSC will be considered at any time when there is a high risk. All risky procedures will be carried out in a validated class II Biosafety cabinet.

4. Emergency/incident response plan: Contingency plans will be developed to reduce the likelihood of exposure to/release of a biological agent, or to reduce the consequences of such incidents by providing specific standard operating procedures (SOPs) to be followed in possible emergency scenarios that apply to the work and local environment. Personnel will be trained on these procedures and have periodic refresher training to maintain competency. First-aid kits, including medical supplies such as bottled eye washes and bandages, will be available and easily accessible to personnel. All incidents will be reported to the appropriate personnel in a timely manner. A written record of accidents and incidents will be maintained. Any incident will be reported and investigated in a timely manner and used for updating laboratory procedures and emergency response plans. Spill kits, including disinfectant, will be easily accessible to personnel. Written procedures for cleaning and decontaminating spills will be developed for the laboratory and followed by suitably trained personnel.

5. COVID-19 prevention: To avoid contamination and or and the spread of the infection, all field personnel will be provided with personal protective material for COVID 19, including face masks and/or visors and alcohol gel. Soap will also be distributed to the health facilities for use by patients.

Appendix 11. Worksheet for monitoring and evaluation of field activities.

**Date**
**Site**
Field
Health Facility
**Samples**
Filter paper
Rapid diagnostic test
**Quality**
Quality of the filter paper
Witness the collection process if applicable
Quantity of blood collected
Quality of the blood collected
Identification of the samples
**Cross-check sample data vs questionnaire and control sheet**
Compatible identification
**Information in source document**
Legibility of Information
Data filled in the right place
Confirm data with original document if applicable
**Total number of documents**
Questionnaire
Informed consent
Requisition form
**Total number of samples**
Filter paper
RDT
Number of discrepant RDT results
**Number of non-compliant documents**
Questionnaire
Informed consent
Requisition form
**Quantity of samples with non-conformity**
Filter paper
RDT
**Evaluated by**
Name
Date
**Revised by**
Name
Date of the next monitoring

Mayor A, *et al. BMJ Open* 2022; 12:e063456. doi: 10.1136/bmjopen-2022-063456
