# Supplementary material for: Prospective surveillance study to detect antimalarial drug resistance, gene deletions of diagnostic relevance and genetic diversity of Plasmodium falciparum in Mozambique: protocol
Source: BMJ Open. 2022 Jul 11;12(7):e063456. doi: 10.1136/bmjopen-2022-063456 (PMC9274532; doi:10.1136/bmjopen-2022-063456)
Supplement: Supplementary data [file bmjopen-2022-063456supp002.pdf]

**Supplementary Table 1.** Control measures for bool collection through fingerprick.

| Potential Hazards | Likelihood | Consequences | Control Measures |
|---|---|---|---|
| Lancet stick injury | Rare | Possibility of infection (hep B or HIV) | Wear PPE and work slowly and carefully. The lancets for finger pricks are designed in such a way that they can only be used once, thereby minimising the possibility of cross contamination. |
| Incorrect blood collection procedure | Moderate | No blood drawn | Follow detailed SOPs for blood collection procedures for finger picks; dispose of all contaminated waste in the biohazard bags. |
| Haematoma | Rare | Bruising and painful lancet entry site | Follow correct collection procedures (SOPs), if unable to draw blood, withdraw the lancet and apply light pressure to the site. Do not attempt to withdraw blood at the same site again. |
| Fainting | Moderate | Subject may feel faint at the sight of blood | Lie the patient down and stay with them until they have recovered. Little sips of water and a wet towel applied to the forehead. Verbal communication throughout the procedure will reassure the subject. |

**Supplementary Table 2.** Roles of each partner organization.

| Organisation | Role |
|---|---|
| **National Malaria Control Programme** | Technical oversight of all research |
| **Malaria Consortium** | Development of the sampling protocol and ethical clearance<br>Training of data collectors<br>Field collection of blood samples and participant data<br>Transfer of samples and data to CISM<br>Creation of a surveillance dashboard<br>Transference of activities to NMCP |
| **Centro de Investigação em Saúde de Manhiça (CISM)** | Financial, organizational and overall coordination<br>Genetic analysis<br>Long-term storage of study samples<br>Analysis of data<br>Scientific/programmatic dissemination of results |
| **National Institute of Health (INS)** | Malaria Indicator and ANC Surveys in Inhambane, Gaza, Maputo and Maputo City<br>Sample analysis |
| **ISGlobal** | Supervision of epidemiological aspects<br>Technical support for sampling strategy, data collection and epidemiological analysis<br>Development of sequencing pipeline |
| **Institute for Disease Modelling** | Development and calibration of an epidemiological genomic model for Mozambique regions |
| **University of California San Francisco** | Development of sequencing and analytical pipelines<br>Development of transmission network model<br>Training and supervision of genetic and bioinformatics activities |



Mayor A, *et al. BMJ Open* 2022; 12:e063456. doi: 10.1136/bmjopen-2022-063456
