# Supplementary material for: Prospective surveillance study to detect antimalarial drug resistance, gene deletions of diagnostic relevance and genetic diversity of Plasmodium falciparum in Mozambique: protocol
Source: BMJ Open. 2022 Jul 11;12(7):e063456. doi: 10.1136/bmjopen-2022-063456 (PMC9274532; doi:10.1136/bmjopen-2022-063456)
Supplement: Author's manuscript [file bmjopen-2022-063456.draft_revisions.pdf]

BMJ Open

BMJ Open is committed to open peer review. As part of this commitment we make the peer review history of every article we publish publicly available.

When an article is published we post the peer reviewers' comments and the authors' responses online. We also post the versions of the paper that were used during peer review. These are the versions that the peer review comments apply to.

The versions of the paper that follow are the versions that were submitted during the peer review process. They are not the versions of record or the final published versions. They should not be cited or distributed as the published version of this manuscript.

BMJ Open is an open access journal and the full, final, typeset and author-corrected version of record of the manuscript is available on our site with no access controls, subscription charges or pay-per-view fees (http://bmjopen.bmj.com).

If you have any questions on BMJ Open's open peer review process please email info.bmjopen@bmj.com

BMJ Open

A prospective surveillance study to detect antimalarial drug resistance, gene deletions of diagnostic relevance and genetic diversity of Plasmodium falciparum in Mozambique

Journal:	*BMJ Open*
Manuscript ID	bmjopen-2022-063456
Article Type:	Protocol
Date Submitted by the Author:	04-Apr-2022
Complete List of Authors:	Mayor, Alfredo; Centro de Investigação em Saúde de Manhiça; ISGlobal<br>da Silva, Clemente; Centro de Investigação em Saúde de Manhiça<br>Rovira-Vallbona, Eduard; ISGlobal<br>Roca-Feltrer, Arantxa ; Malaria Consortium<br>Bonnington, Craig; Malaria Consortium<br>Wharton-Smith, Alexandra; Malaria Consortium<br>Greenhouse, Bryan; University of California San Francisco, Medicine<br>Bever, Caitlin; Bill & Melinda Gates Foundation<br>Chidimatembue, Arlindo; Centro de Investigação em Saúde de Manhiça<br>Guinovart, Caterina; Barcelona Institute for Global Health<br>Proctor, Josh; Bill & Melinda Gates Foundation<br>Rodrigues, Maria; Malaria Consortium<br>Canana, Neide; Malaria Consortium<br>Arnaldo, Paulo; Instituto Nacional de Saúde<br>Boene, Simone; Centro de Investigação em Saúde de Manhiça<br>Aide, Pedro; Centro de Investigação em Saúde de Manhiça<br>Enosse, Sonia; Malaria Consortium<br>Saute, Francisco; Centro de Investigação em Saúde de Manhiça<br>Candrinho, Baltazar; Ministry of Health
Keywords:	Epidemiology < INFECTIOUS DISEASES, Molecular diagnostics < INFECTIOUS DISEASES, Public health < INFECTIOUS DISEASES, MOLECULAR BIOLOGY, PARASITOLOGY, Genetics < TROPICAL MEDICINE

SCHOLARONE™
Manuscripts

A prospective surveillance study to detect antimalarial drug resistance, gene deletions of diagnostic relevance and genetic diversity of *Plasmodium falciparum* in Mozambique

Alfredo Mayor[1,2,3,4], Clemente da Silva[1], Eduard Rovira-Vallbona[2], Arantxa Roca-Feltrer[5], Craig Bonnington[5], Alexandra Wharton-Smith[5], Bryan Greenhouse[6], Caitlin Bever[7], Arlindo Chidimatembue[1], Caterina Guinovart[2], Josh Proctor, Maria Rodrigues[5], Neide Canana[5], Paulo Arnaldo[8], Simone Boene[1], Pedro Aide[1], Sonia Enosse[5], Francisco Saúte[1], Baltazar Candrinho[9]

1. Centro de Investigação em Saúde de Manhiça (CISM), Manhiça, Mozambique
2. ISGlobal, Hospital Clínic - Universitat de Barcelona, Barcelona, Spain
3. Department of Physiologic Sciences, Faculty of Medicine, Universidade Eduardo Mondlane, Maputo, Mozambique
4. Spanish Consortium for Research in Epidemiology and Public Health (CIBERESP), Madrid, Spain
5. Malaria Consortium, Maputo, Mozambique
6. University of California San Francisco, USA
7. Institute for Disease Modeling, Bill and Melinda Gates Foundation, USA
8. Instituto Nacional de Saúde, Maputo, Mozambique
9. National Malaria Control Program, Ministry of Health, Mozambique

Corresponding autor: Alfredo Mayor, Centro de Investigação em Saúde de Manhiça (CISM), Maputo, Moçambique. Telephone (+ 258) 21 810 002; e-mail: alfredo.mayor@isglobal.org

Key words: Malaria, genomics, pregnancy, next generation sequencing, surveillance

Abtract: 303
Text: 4286

For peer review only

Abstract

Introduction

Genomic data constitutes a valuable adjunct to routine surveillance that can guide programmatic decisions to reduce the burden of infectious diseases. However, genomic capacities remain low in Africa. This study aims to operationalize a functional malaria molecular surveillance system in Mozambique for guiding malaria control and elimination.

Methods and analyses

This prospective surveillance study seeks to generate *P. falciparum* genetic data to 1) monitor molecular markers of drug resistance and deletions in rapid diagnostic test targets; 2) characterize transmission sources in low transmission settings; and 3) quantify transmission levels and the effectiveness of antimalarial interventions. The study will take place across nineteen districts in nine provinces (Maputo city, Maputo, Gaza, Inhambane, Niassa, Manica, Nampula, Zambézia and Sofala) which span a range of transmission strata, geographies and malaria intervention types. Dried blood spot samples and rapid diagnostic tests will be collected across the study districts in 2022 and 2023 through a combination of dense (all malaria clinical cases) and targeted (a selection of malaria clinical cases) sampling. Pregnant women attending their first antenatal care visit will be also included to assess their value for molecular surveillance. We will use a multiplex amplicon-based next generation sequencing approach targeting informative single nucleotide polymorphisms, gene deletions and microhaplotypes. Genetic data will be incorporated into epidemiological and transmission models to identify the most informative relationship between genetic features, sources of malaria transmission and programmatic effectiveness of new malaria interventions. Strategic genomic information will be ultimately interagted into the national malaria informationand surveillance system to improve the use of the genetic information for programmatic decision-making.

Ethics and dissemination

The protocol was reviewed and approved by the institutional (CISM) and national ethics committees of Mozambique and the Hospital Clinic of Barcelona. Project results will be presented to all stakeholders and published in open-access journals.

Study registration number: ClinicalTrials.gov NCT05306067

For peer review only - http://bmjopen.bmj.com/site/about/guidelines.xhtml

Strengths and limitations of this study

- Next generation sequencing will be performed in country through the establishment of technical and computational infrastructure as well as analytical tools.
- The project builds from recent elimination experiences in southern Mozambique and uses a biorepository of already collected *P. falciparum* samples to select multi-allelic short-range haplotypes (microhaplotypes) that increase the power of biallelic loci for phase inference in polygenomic infections.
- A joint epidemiological-genetic analysis will enable better predictions of the operational efficacy of new interventions.
- We will assess the value of a new surveillance systems at antenatal visits to improve the programmatic performance of malaria control and elimination activities.
- More evidence on the association between malaria transmission intensity and genetic data is required for the use of malaria molecular surveillance data to assess the effectiveness of malaria interventions.

Introduction

Pathogen genomics has the potential to transform the surveillance, prevention and control landscape of infectious diseases. The rapid innovation in sequencing technologies has led to the development of robust next-generation sequencing (NGS) equipment with the ability for high pathogen resolution at increasingly affordable prices. This development has subsequently facilitated the incorporation of pathogen genomics in disease surveillance systems in high-income countries, allowing for targeted and effective control of disease threats through the timely and in-depth pathogen characterisation[1]. Genomics-based surveillance is therefore becoming an integral strategy towards control and elimination of diseases such as COVID19, tuberculosis, malaria, HIV and foodborne pathogens, among others[2].

The strategic use of genetic variation in *P. falciparum* can boost the capacity of malaria control and elimination programs to deploy the most efficient interventions[3]. Molecular tools and use cases for decision making are concurrently being considered by the World Health Organization (WHO) which, through a technical consultation on the role of parasite and anopheline genetics in malaria surveillance[4], identified different levels of action based on evidences available. Genetic data can flag the emergence of mutations conferring resistance to antimalarials (i.e., artemisinins)[5] or deletions that affect rapid diagnostic test (RDT) sensitivity (i.e., *histidine-rich protein 2* [*hrp2*])[6-8]. Genomic scans for selection[9] can identify other parasite adaptations mediated by single nucleotide polymorphisms (SNPs) and structural variations (gene copy number[10]) that may require a programmatic response. Parasite relatedness metrics such as identity by descent (IBD)[11] can be used to characterize the key drives of ongoing transmission, to identify foci[12 13] and to discriminate between indigenous and imported cases in areas approaching elimination[14-16]. Bottlenecks in parasite population driven by control and elimination efforts have been shown to reduce *P. falciparum* genetic diversity and increase similarity due to inbreeding and recent common ancestry[17]. These evidences provide the basis for modelling efforts to recapitulate features of malaria transmission from genetic data and inform about the effectiveness of antimalarial interventions[18-23]. However, further evidence is needed to demonstrate the feasibility and appropriateness of using genetic data as a proxy for transmission intensity and define the conditions under which that feasibility applies. Moreover, standardised approaches for detecting resistance through molecular markers are lacking, and variation in sample type, collection, storage, DNA extraction, marker detection and analysis of results can undermine the comparability of findings, as well as the sensitivity and specificity of methods used. Adequate genotyping methods, sampling frameworks, analytical pipelines and demonstration studies are still required across a range of malaria intensities, programmatic environments and use scenarios.

Strategic *P. falciparum* genetic information can be integrated into innovative cost-efficient surveillance approaches, such as those targeting pregnant women attending antenatal care (ANC) clinics[24]. Women at ANC are a generally healthy, easy-access population, contributing valuable data for infectious disease surveillance (ie, HIV[25] and syphilis[26]) and wider health metrics at the community level, including a proxy of the malaria burden in the community[27-32]. Moreover, ANC-level malaria surveillance can provide a routine measure of the malaria burden in pregnancy, which countries lack, whilst potentially improving pregnancy outcomes by treating infections at first trimester. Women attending ANC also provide an attractive sampling population for measures of exposure to malaria beyond simply presence or absence of parasite infection. In particular, in addition to measuring complexity of infection or parasite flow-rates between populations, molecular analysis

For peer review only - http://bmjopen.bmj.com/site/about/guidelines.xhtml

of *P. falciparum* isolates collected from pregnant women may provide a means for the identification of adaptations developed by the parasite to control strategies, such as antimalarial resistance and deletions of antigens targeted by rapid diagnostic test that can compromise diagnosis, treatment and prevention.

Despite the potential benefits and the greater need to control the high burden of infectious diseases, genomic surveillance capacity remains low for many public health programmes in Africa[2]. In order to reduce inequities in the access to sequencing technologies, this project aims to promote capacities in Mozambique for operationalizing a functional malaria molecular surveillance (MMS) system for decision making[4]. Mozambique is among the ten countries with the highest burden of malaria worldwide, with an estimated 10.8 million cases in 2020[33]. However, malaria transmission is very heterogeneous in the country, with a high burden in the north and very low transmission in the south. Therefore, the project aims to address National Malaria Control Program (NMCP) programmatic needs for elimination initiatives in southern Mozambique and burden reduction in the north (**Figure 1**).

Methods and analysis

Study design

This is a prospective genomic surveillance study of *P. falciparum* isolates to be collected between 2022 and 2023 from a variety of transmission intensities and geographies in Mozambique to inform three use cases: appropriate malaria diagnostics and treatment; characterizing transmission sources in low transmission settings; and identifying intervention mixes with optimal effectiveness to reduce burden in moderate-to-high transmission areas. To achieve this, three different sampling approaches will be performed. First, all malaria cases will be sampled throughout the year in two low transmission districts of southern Mozambique currently targeted by reactive malaria surveillance activities (*dense sampling).* Second, a targetted approach will aim to collect a predefined number of samples at selected health facilities in the country. In low transmission settings, sampling will be conducted throughout the year, while two surveys will be conducted in medium-to-high transmission settings: one during the rainy and a second one during the dry season (which extend from November to April and May to October, respectively). During the high transmission (rainy) season, an LDH-based RDT will be added to the standard routine HRP2-based diagnostics to identify potential false negative results due to *hrp2/3* deletions among clinical cases[34]. And third, *ANC sampling* of pregnant women at first attendance will be conducted throughout the year at selected health facilities in the country. The overarching sampling strategy for the study will however remain flexible and iterative, informed by sample analysis as the study progresses, and in view of future sampling and research activities being conducted by the Ministry of Health, National Institute of Health and other stakeholders in Mozambique, to avoid sampling overlap and ensure a diversity of sampled locations.

The project will also leverage from clinical trials and surveillance activities being conducted in Mozambique between 2021 and 2024, namely: the Malaria Indicator Survey (2022-2023) in southern Mozambique; the therapeutic efficacy survey (2022) in sentinel sites in the country (Montepuez in Cabo Delgado, Moatize in Tete, Dondo in Sofala, Mopeia in Zambézia and Massinga in Inhambane)[35 36]; reactive surveillance activities in Magude and Mautuine (Maputo Province); a Phase III cluster-randomized, open-label, clinical trial in 2022 to study the safety and efficacy of ivermectin mass drug administration to reduce malaria transmission in Mopeia District (Zambeia Province); a large-scale implementation development project aiming at maximising the delivery and uptake of intermittent preventive treatment in infancy (IPTi) in Massinga District (Inhambane; 2022-2024); a hybrid effectiveness-implementation study to evaluate the feasibility and effectiveness of seasonal malaria chemoprevention (SMC) with sulfadoxine-pyrimethamine and amodiaquine in Lalaua and Muecate districts (Nampula Province; 2022); and a programmatic delivery of a population-based mass drug administration with dihydroartemisinin-piperaquine in Manjacaze district (Gaza Province; 2022-2023).

Study settings and participants

Eight provinces were identified through consultation with the NMCP for inclusion in the study: Maputo, Gaza, Inhambane, Niassa, Manica, Nampula, Zambezia and Sofala. Selection of study sites will be stratified by transmission intensity into two major strata: A) low transmission (Maputo city and Maputo Province, where individual case notification is being implemented to reach interruption of transmission), and B) medium-to-high

For peer review only - http://bmjopen.bmj.com/site/about/guidelines.xhtml

transmission areas (Gaza, Inhambane, Niassa, Manica, Nampula, Zambezia and Sofala provinces, targeted by burden-reducing strategies). Overall, a total of 19 districts will be included, which provide a diverse range of epidemiological settings (see **Table 1** and **Figure 2**).

Table 1. Study provinces and districts targeted in the protocol.

Transmission	Region	Province	District	Sampling: Dense	Sampling: Targeted HFS	Sampling: Targeted ANC	Sampling: Other sources
Low	South	Maputo City	Kamavota		X[1]		
			KaMaxaqueni		X[1]		
			Nlhamankulu		X[1]		
		Maputo Province	Boane		X[1]		
			Manhiça		X[1]		
			Magude	X[1]		X[2]	React
			Matutuine	X[1]			React
Medium-to-high		Gaza	Manjacaze		X[3]	X[2]	MDA-DP
		Inhambane	Maxixe		X[3]	X[2]	
			Massinga				IPTi & TES
		Manica	Guro & Gondala		X[3]	X[2]	
	Central	Sofala	Chemba		X[3]	X[2]	
			Dondo				TES
		Tete	Moatize				TES
		Niassa	Cuamba		X[3]	X[2]	
	North	Nampula	Mecuburi & Malema		X[3]	X[2]	
			Lalaua & Muecate				SMC
		Zambezia	Mopeia		X[3]		MDA-IVM & TES
		Cabo Delgado	Montepuez				TES

ANC, Antental care clinics; HFS, health facility survey; IPTi, intermittent preventive treatment in infancy; MDA-DP, Mass drug administration with dihydroartemisinin-piperaquine; MDA-IVM, Mass drug administration with Ivermectin; REACT, Reactive surveillance; SMC, seasonal malaria chemoprevention; TES, Therapeutic efficacy study.

1, Year round, all ages
2, Year round, first ANC visit
3, Rainy & Dry season; 2-10 years of age

Dense sampling will be conducted in the low transmission districts of Magude and Matutuine, where all the individuals of any age (>6 months old) with clinical symptoms of malaria (defined as axillary temperature ≥37.5ºC or history of fever in the preceding 24 hours) and a parasitologically confirmed malaria diagnosis via RDT or microscopy (**Table 2**) will be invited to donate their RDT for molecular analysis (dense sampling).

Table 2. Study eligibility criteria

For peer review only - http://bmjopen.bmj.com/site/about/guidelines.xhtml

INCLUSION CRITERIA	EXCLUSION CRITERIA
**Low transmission**	
- Any age	- Any symptoms of severe malaria
- Fever (axillary temperature ≥37.5ºC) or history of fever in the preceding 24 hours	- Negative parasitological test for malaria via RDT or microscopy (except any women at their first ANC visit, who will be recruited before testing for malaria with an RDT)
- Positive parasitological test for malaria diagnosis via RDT or microscopy	- Unwilling to provide informed, written consent
- Household contact of someone with fever/history of fever and *P. falciparum* positive RDT	- History of antimalarial treatment in the last 14 days
OR	
- Pregnant women attending first antenatal care visit in Magude district	
AND	
- Informed, written consent to participate from participant and/or guardian	
**High transmission**	
- Children aged 2-10 years of age Fever (axillary temperature ≥37.5ºC) or history of fever in the preceding 24 hours	- Any symptoms of severe malaria
- Positive parasitological test for malaria diagnosis via RDT* or microscopy	- Negative parasitological test for malaria via RDT or microscopy (except any women at their first ANC visit, who will be recruited before testing for malaria with an RDT)
OR	
- Pregnant women attending first antenatal care visit	- Unwilling to provide informed, written consent
AND	- History of antimalarial treatment in the last 14 days
- Informed, written consent to participate from participant and/or guardian	

*a second RDT (HRP2-pLDH) will be provided in these locations to support detection of *P. falciparum hrp2* deletions.

Targetted sampling will be conducted at selected health facilities in the low transmission districts of Boane, Manhiça and Maputo City (KaMavota, KaMaxaqueni and Nhamankulu Districts), where a drop of blood will be collected onto filter paper from consenting individuals of any age (>6 months old) with confirmed clinical malaria. In medium-to-high transmission areas, targeted sampling will focus on children aged 2-10 years of age attending selected health facilities with clinical symptoms of malaria and a parasitologically confirmed malaria diagnosis via RDT (**Table 2**). Ten health facilities will be targeted in each district.

Pregnant women attending their first antenatal care visit (any trimester) will be invited to participate both in low (Magude in Maputo Province) and high transmission districts (Maxixe in Inhambane, Manjacaze in Gaza, Mecuburi and Malema in Nampula, Cuamba in Niassa, Guro and Gondala in Manica, Chemba in Sofala), irrespectively of malaria clinical symptoms.

Enrolment of participants

Dense sampling in Magude and Matutuine districts will be coordinated with district malaria focal points, community health workers (CHW), malaria volunteers (who provide a link between the CHW and the health facility, and assist the CHW in the follow-up of cases and administration of medication) and health facilities. All *P. falciparum* positive RDTs (SD Bioline Malaria Ag Pf, 05FK50, Abbott) will be stored for molecular analysis. RDTs of *P. falciparum*-confirmed household contacts will be also collected to estimate the rate of within-household transmission. Targeted sampling through health facility-based surveys (HFS) in low and medium-to-high transmission settings will be carried out by one team comprised of one maternal and child health nurse, a laboratory technician or a medical technician. The number of people to be screened in each health facility and the duration of recruitment to achieve the sample size will be dependent on the RDT-positivity rate among people meeting the eligibility criteria. A second test including a non-HRP2 line (StandardQ

For peer review only - http://bmjopen.bmj.com/site/about/guidelines.xhtml

Malaria Pf/Pan Ag Test, SD Biosensor) will be carried out in HFS during the rainy season and discrepant results suggestive of *pfhrp2/3* deletions will be recorded and further analysed to confirm the deletion. Nurses at the ANC clinics will be in charge of the recruitment of pregnant women at their first visit. Pregnant women will be tested for malaria using a routine RDT and the result will be recorded in a standard questionnaire, together with routine ANC tests. Each enrolled individual will be assigned with a unique identification (UID) number and a barcode.

Data and sample collection

Field workers and nurses will be trained to ask for informed consent (**Appendix 1-4**), perform a simple questionnaire (**Appendix 5-8**) and collect biological samples for molecular analysis. The survey questionnaire will be administered to all study participants or children's parents/guardians meeting the inclusion criteria and will include inclusion criteria check, characteristics of the participant and malaria related information. For pregnant participants, data will be collected on parity and gestational age at first ANC visit, as well as information related with malaria and use of preventive measures. A telephone contact number will be collected from pregnant women in low transmissions settings in order to locate their residence for spatial analysis. A Site Coordinator will be responsible for supervising the work of field workers, nurses and the data entry clerk, and reviewing and comparing questionnaires and samples for correct matching, completeness and accuracy.

Nurses will be trained to collect blood by finger pricking (**Supplemental Table 1**) following standard (**Supplementary Appendix 9**) and COVID19 safety procedures (**Supplementary Appendix 10**). For each participant, either the *P. falciparum*-positive RDT used for routine malaria diagnosis (dense sampling) or four blood spots onto two filter papers (Whatman® 3MM; targeted sampling) will be collected. Specimens will be labelled anonymously (patient UID, study health facility and date), dried for 24 hours and kept in individual plastic bags with desiccants at 4ºC. Every two to six weeks, the completed questionnaires, informed consents and samples will be sent to the data entry clerk at CISM through a local transportation agency. Informed consents will be received by study investigators. A data manager will be responsible for the receipt of the informed consents and double data entry at CISM, and a laboratory technician will be responsible for receiving the samples and store them at -20ºC until analysis. Part of the dried blood spot will be stored in RNA-preserving solution. All samples will be kept in the CISM Laboratory for a period of approximately 15 years. For quality control purposes, up to 5% of the samples will be analyzed at UCSF (San Francisco, USA) and/or ISGlobal (Barcelona, Spain). In order to identify errors in data or sample collections and take necessary corrective actions, a standardized checklist (**Supplementary Appendix 11**) will be filled in by the monitoring officer during biweekly monitoring visits.

Molecular analyses

Informative SNPs (including -but not restricted to- markers of resistance to artemisinin [*pfkelch13*][37], sulfadoxine-pyrimethamine [*pfdhfr, pfdhps]*[38], or chloroquine [*pfcrt*][39]), microhaplotypes[40] and *pfhrp2* and *pfhrp3* regions[6-8]) will be targeted using multiplexed primers on flanking sequences, with a range of amplicon size of ~225-275 bp (covered by a paired end read). Targeted amplicons obtained by PCR on genomic DNA using Illumina-specific adaptors and sample-specific barcode will be pooled to create a single product library, which will be sequenced (paired-end 150-bp) on a Miseq Illumina sequencer in the country or higher performing equipment when available. Amplicon representation and SNP and haplotype calling will be assessed in demultiplexed and trimmed sequencing reads after filtering sequencing errors. The designed panel will be validated using mixtures of *P. falciparum* lines to determine precision and repeatability. Samples will be also used for other molecular analysis of programmatic interest, such as the detection of *Plasmodium* species, parasite antigens, serological markers of parasite exposure (antibodies) and parasite RNA-based markers (i.e., gametocytes). A quality control program based on the sequencing of an artificially-created set of samples (i.e. mixtures of known laboratory controls at specific proportions and densities) will be processed at predefined times to guarantee the quality of the processes during the life of the project.

Data Management

Data will be collected using paper (targeted sampling) and password-protected electronic devices (dense sampling). Data collected using paper will be double entered into the study database using RedCap[41]. Automatic quality checks will be performed to ensure data completeness. Confidentiality and secxurity will be ensured through automatic encryption of sensitive data, storage in password protected computers and locked

For peer review only

For peer review only - http://bmjopen.bmj.com/site/about/guidelines.xhtml

locations, and data sharing using password-protected, encrypted files. Prior to analysis, data will be de-identified with the exception of geo-location codes, which are necessary for specific analyses. The study will also use data available from the NMCP, including intervention coverage, historical prevalence surveys, travel history or other mobility assessments, and entomological data. Sequences generated through the analysis of samples will be integrated into a curated catalogue of genomic data together with relevant anonymized clinical and epidemiological information and will be made publicly available in public repositories such as the European Nucleotide Archive (ENA) and MalariaGen Resource Center. In order to facilitate data accessibility and use, and to obtain a meaningful integration with other sources of surveillance data, genetic information will be incorporated into the DHIS2-based Integrated malaria information storage system (iMISS), which is currently being rolled out in Mozambique[42].

Study outcomes and sample size calculations

The primary endpoints are: a) Prevalence of molecular markers of diagnostic and antimalarial resistance by period, study area and population (use case 1); b) Genetic relatedness indicators between pairs of samples and populations by period, study area and population (use case 2); and c) Genetic diversity indicators by period, study area and population (use case 3). Sample size per sampling domain (Province) has been estimated considering antimalarial and diagnostic resistance as a primary use case, considering the negligible carriage of molecular markers of artemisinin resistance[5] and *pfhrp2/3* deletions[6] in Mozambique, and setting 5% as the warning threshold [43]. Assuming a 10% of loss of samples or uninterpretable analysis, a sample size of up to 500 per sampling domain would be adequate to: a) estimate a proportion of 0.05 (markers of drug resistance or *pfhrp2* deletion) with 0.026 absolute precision and 95% confidence and b) achieve a power of 80% for detecting an increase of genetic marker (resistance or deletion) from 0 ‰ to 5% at a two-sided p-value of 0.01. A flexible and adaptive sampling scheme will be followed, where a) estimates generated during the first half of the project will inform subsequent sampling schemes and b) not all the samples collected will be analysed (some of them will be stored as reference materials, for confirmation of findings or future studies on Plasmodium biology). The number of pregnant women to be recruited in order to reach the sample number will depend on the parasite rates in the study areas; assuming an overall RDT positivity rate of 25%, we expect we will be needing to recruit a total of 2,000 pregnant women per site to get 500 *P. falciparum* positive samples, although numbers may differ between sites.

Analysis Plan

Demographic and clinical charateristics of study participants will be described using summary statistics. A user-friendly and locally executable bioinformatic pipeline will be developed for analysis of *P. falciparum* targeted sequencing data. Highly informative SNPs and microhaplotypes showing geographic structuring will be selected using a supervised machine learning approach trained by genomes from known geographic origin in Mozambique. Population-level genetic diversity will be quantified using expected heterozygosity (He), number of alleles per locus, allele frequency, complexity of infection (COI)[23] as well as other genetic metrics. Deletions and copy number variations will be assessed based on sequencing coverage ratios[10,44]. We will use regression models adjusted by potential confounders (demographic and clinical factors, among others) to compare genetic metrics between seasons, before and after the antimalarial interventions, between pregnant women and community sampling populations and across different intensities of malaria transmission. Finally, we will integrate genomic surveillance data into epidemiological and transmission network models. For the first one, we will leverage two recent models developed at the Institute of Disease Modelling[45] (a malaria genetic model calibrated to a longitudinal genetic study in Senegal[18] and a disease transmission model calibrated with the Magude data) to build an end-to-end malaria transmission and genetics model for Mozambique (**Figure 3**). The transmission network model will include data for densely sampled in low transmission areas on individual and community-level case classification (imported, local, introduced), the extent and duration of sustained local transmission and how these change over space and time. Summary indicators will be visualized in graphical and tabular forms in the iMISS through genetic dashboards. We will establish risk profile algorithms and interpretation components that are capable of generating outputs on a) country-wide antimalarial resistance profiles (rolling-basis); b) in very low transmission areas (e.g. Magude district), genetic connectivity and case classification (together with travel history and other parameters obtained from case-based notification tools); and c) "high burden to high impact"-specific analysis (i.e., stratification and trend analyses for exploring the potential impact of intervention mixes implemented).

For peer review only - http://bmjopen.bmj.com/site/about/guidelines.xhtml

Ethics and dissemination

Written informed consent will be sought from all study participants before blood sample collection is conducted (**Appendix 1)**. Two copies will be signed, one will be kept by participant and the other by the investigators in a locked space. The information sheet and consent form will also include text explaining informed consent for future use of biological specimens to conduct additional analyses of the Plasmodium parasite. In case of minors (less than 18 years of age), consent will be sought from parents, relatives or guardians. Informed consents will specify that the data will be made public. First line treatment for malaria will be provided to the enrolled participants in line with national treatment guidelines. Considerations related to preventing the risk of SARS-COV-2 transmission are detailed in **Supplementary Appendix 10**. There will not be any economic incentive to participate in the study. Transference of data and materials out of Mozambique will be done only when appropriate data and material transfer agreements are signed between participating institutions (**Supplementary Table 2**).

Patient and Public Involvement

Patients and the public were not involved in the development of this protocol.

For peer review only

Discussion

There is a growing acceptance that genomics can play a critical role in policy and programmatic decisions. With the aim of demonstrating the programmatic application and feasibility of malaria genomic surveillance in Mozambique, we will generate parasite genomic data across varying transmission scenarios for supporting strategic decision-making. First, MMS data will inform drug and diagnostic choices through the monitoring of molecular markers of antimalarial and diagnostic resistance. The emergence of *hrp2/3* deletions[6-8], resistance to artemisinin[37] and partner drugs, as well as the resistance to sulfadoxine-pyrimethamine (SP) used for intermittent preventive treatment (in both pregnancy and infancy) and seasonal malaria chemoprofilaxis[38 46 47], threatens the global effort to reduce the burden of malaria[33]. The WHO recommends that countries with reports of *pfhrp2/3* deletions, and neighbouring countries, should conduct representative baseline surveys among suspected malaria cases. If the prevalence of molecular markers of antimarial resistance or deletions causing false negative RDT results reaches the threshold of >5%, then there is need to consider alternative antimalarials and RDTs. Second, the project will help to target the reservoirs sustaining transmission by quantifying parasite importation, identifying sources and characterizing local transmission in near-elimination settings[48 49]. Genomic surveillance and phylogenetic analyses have enabled the near real-time estimation of transmission chains of non-sexually recombining, rapidly evolving pathogens such as Ebola[50], influenza[51] and COVID19[52]. However, molecular and analytic advancements are stil required to characterise transmission patterns of pathogens such as *P. falciparum* with a sexually recombining stage[49]. Third, the project will assess the value of *P. falciparum* genetic diversity measures to supplement traditional surveillance for improving stratification, monitoring and impact evaluations in different epidemiological contexts, especially where surveillance data is sparse. This use case still requires development of analytical and interpretative to infer malaria burden[18 20 53-58] and effectiveness of interventions[18-23 53 59-61], as well as validation of sampling frameworks[4]. Finally, the project will test if parasite populations within pregnant women are representative of the general population and expand the usefulness of this approach to inform genomic surveillance indicators.

The project will use state-of-the-art sequencing and modelling approaches. Current *P. falciparum* genetic markers based on biallelic SNPs have limited support for polyclonal samples, which are frequent across all transmission intensities, and have limited resolution to calculate genetic relatedness between parasites, to estimate allele frequencies[23 62], or to distinguish geographic origin[21 23 63]. Multi-allelic short-range haplotypes (microhaplotypes) covered by a single read from high-throughput DNA sequencers allow an accurate statistical inference of phase and have the potential to derive more accurate information than biallelic loci[64-66], particularly in polyclonal infections, to tailor the genomic tool to specific transmission and geographic settings. In addition to being useful for identification and lineage/family relationships, microhaps can provide information on biogeographic ancestry and can be useful for strain detection and deconvolution[64-67]. Methods such as IBD[11 68 69] that can exploit the signal left by recombination on these microhaplotypes may have the power to detect geographic differentiation at small spatial scales relevant for malaria control programs. Machine learning approaches[70] will be used for the selection of key SNPs and microhaplotypes that allow accurate inference of malaria transmission and geographical origin. Finally, models that integrate genomic and epidemiological data

For peer review only - http://bmjopen.bmj.com/site/about/guidelines.xhtml

will be developed to assess the programmatic effectiveness of new malaria interventions and characterize sources of malaria transmission (imported versus local)[45].

This project, guided by programmatic priorities and based on collaborative efforts, aims to boost the use of the genetic data for decision making. To successfully achieve this, the project is grounded on three main principles: a) strengthen sequencing capacities to implement a robust MMS system; b) strong partnership and coordination to make MMS data sharing common practice for malaria control and elimination; and c) effective operationalization of MMS implementation activities. Technical capacities will be built by establishing at CISM a sequencing platform and ancillary equipment for library preparation and quality control. Computational infrastructure and analytical tools will be also developed by establishing a user-friendly automated platform to analyse genomic data with simplified interpretation into actionable information. Training activities will target molecular biologists for wet laboratory analysis, a bioinformatician and molecular epidemiologists for data analysis and interpretation and a field epidemiologist for interpretation of the generated data, and public health specialists for adoption of the findings into policy. Genetic data-to-action culture and engagement of NMCP on genetic analysis will be promoted by integrating genetic aspects in the NMCP activities (i.e., data review meetings) as well as in training and annual meetings, by integrating genetic information with other surveillance data onto the iMISS, and by documenting all the processes, successes and failures to inform future molecular activities. The project will pursue the use of MMS data as an adjunct to traditional surveillance information for elimination initiatives in southern Mozambique and burden reduction in the north through the engagement with regional malaria elimination initiatives (e.g. E8 and MOSASWA, a trilateral initiative to eliminate malaria from Mozambique, South Africa, and Swaziland[71,72]) and linking decision making with the 'high burden to high impact' initiative under the guidance of WHO.

We expect that the genomic intelligence developed through this project will complement current and new surveillance systems to drive decision-making for the control and eventual elimination of malaria in Mozambique and other malaria endemic countries. However, further steps are required beyond this three-year project. Enabling policies and regulatory mechanisms for sample storage and sharing[73], adequate procurement of materials and infrastructure, as well as local expertise for equipment installation and maintenance, need to be developed ofr an effective integration of genomic surveillance into public health. Countries, with appropriate support from mainstream funding bodies, should also develop sustainability plans as part of national disease control programmes, emergency responses, and other surveillance programmes (i.e., antimicrobial resistance) to ensure resources for genomic surveillance. Finally, regular assessments of the efficiency and effectiveness of incorporating genomic data in routine public health surveillance systems will be crucial to stimulate the use of genetic data for policy making.

Ethics approval: The protocol has been approved by the institutional (CISM; Ref: CIBS-CISM/044/2021) and national (Ref: 604/CNBS/21) ethics committees of Mozambique and the Hospital Clinic of Barcelona (Ref. HCB/2022/097).

Authors' contributions: Conceived and designed the study: AM, CB, ARF, BG. Gave inputs to protocol methodology: BC, CG, AC, ERV, CS, FS, SE, PA, SB, MR, NC, PA. Wrote the first draft of the manuscript: AM. Wrote, reviewed and approved the manuscript: all authors.

Funding statement: This work is supported by Bill and Melinda Gates Foundation grant number INV-019032.

Competing interests: There are no competing interests for any author.

For peer review only - http://bmjopen.bmj.com/site/about/guidelines.xhtml

References

1. Armstrong GL, MacCannell DR, Taylor J, et al. Pathogen Genomics in Public Health. *N Engl J Med* 2019;381(26):2569-80. doi: 10.1056/NEJMsr1813907
2. Inzaule SC, Tessema SK, Kebede Y, et al. Genomic-informed pathogen surveillance in Africa: opportunities and challenges. *Lancet Infect Dis* 2021;21(9):e281-e89. doi: 10.1016/S1473-3099(20)30939-7 [published Online First: 20210212]
3. Tessema SK, Raman J, Duffy CW, et al. Applying next-generation sequencing to track falciparum malaria in sub-Saharan Africa. *Malar J* 2019;18(1):268. doi: 10.1186/s12936-019-2880-1 [published Online First: 2019/09/04]
4. WHO. Meeting report of the technical consultation on the role of parasite and anopheline genetics in malaria surveillance 2019 [Available from: https://www.who.int/publications/m/item/meeting-report-of-the-technical-consultation-on-the-role-of-parasite-and-anopheline-genetics-in-malaria-surveillance.
5. Gupta H, Macete E, Bulo H, et al. Drug-Resistant Polymorphisms and Copy Numbers in Plasmodium falciparum, Mozambique, 2015. *Emerg Infect Dis* 2018;24(1):40-48. doi: 10.3201/eid2401.170864 [published Online First: 2017/12/21]
6. Gupta H, Matambisso G, Galatas B, et al. Molecular surveillance of pfhrp2 and pfhrp3 deletions in Plasmodium falciparum isolates from Mozambique. *Malar J* 2017;16(1):416. doi: 10.1186/s12936-017-2061-z [published Online First: 2017/10/19]
7. Gamboa D, Ho MF, Bendezu J, et al. A large proportion of P. falciparum isolates in the Amazon region of Peru lack pfhrp2 and pfhrp3: implications for malaria rapid diagnostic tests. *PLoS One* 2010;5(1):e8091. doi: 10.1371/journal.pone.0008091
8. Agaba BB, Yeka A, Nsobya S, et al. Systematic review of the status of pfhrp2 and pfhrp3 gene deletion, approaches and methods used for its estimation and reporting in Plasmodium falciparum populations in Africa: review of published studies 2010-2019. *Malaria journal* 2019;18(1):355. doi: 10.1186/s12936-019-2987-4 [published Online First: 2019/11/07]
9. Mobegi VA, Duffy CW, Amambua-Ngwa A, et al. Genome-wide analysis of selection on the malaria parasite Plasmodium falciparum in West African populations of differing infection endemicity. *Mol Biol Evol* 2014;31(6):1490-9. doi: 10.1093/molbev/msu106 [published Online First: 2014/03/20]
10. Simam J, Rono M, Ngoi J, et al. Gene copy number variation in natural populations of Plasmodium falciparum in Eastern Africa. *BMC Genomics* 2018;19(1):372. doi: 10.1186/s12864-018-4689-7 [published Online First: 2018/05/23]
11. Schaffner SF, Taylor AR, Wong W, et al. hmmIBD: software to infer pairwise identity by descent between haploid genotypes. *Malar J* 2018;17(1):196. doi: 10.1186/s12936-018-2349-7 [published Online First: 2018/05/17]
12. Rice BL, Golden CD, Anjaranirina EJ, et al. Genetic evidence that the Makira region in northeastern Madagascar is a hotspot of malaria transmission. *Malar J* 2016;15(1):596. doi: 10.1186/s12936-016-1644-4 [published Online First: 2016/12/22]
13. Spanakos G, Snounou G, Pervanidou D, et al. Genetic Spatiotemporal Anatomy of Plasmodium vivax Malaria Episodes in Greece, 2009-2013. *Emerg Infect Dis* 2018;24(3):541-48. doi: 10.3201/eid2403.170605 [published Online First: 2018/02/21]
14. Chang HH, Wesolowski A, Sinha I, et al. Mapping imported malaria in Bangladesh using parasite genetic and human mobility data. *Elife* 2019;8 doi: 10.7554/eLife.43481 [published Online First: 2019/04/03]
15. Chenet SM, Silva-Flannery L, Lucchi NW, et al. Molecular Characterization of a Cluster of Imported Malaria Cases in Puerto Rico. *Am J Trop Med Hyg* 2017;97(3):758-60. doi: 10.4269/ajtmh.16-0837 [published Online First: 2017/07/28]
16. Patel JC, Taylor SM, Juliao PC, et al. Genetic Evidence of Importation of Drug-Resistant Plasmodium falciparum to Guatemala from the Democratic Republic of the Congo. *Emerg Infect Dis* 2014;20(6):932-40. doi: 10.3201/eid2006.131204 [published Online First: 2014/05/27]
17. Daniels RF, Schaffner SF, Bennett A, et al. Evidence for Reduced Malaria Parasite Population after Application of Population-Level Antimalarial Drug Strategies in Southern Province, Zambia. *Am J Trop Med Hyg* 2020 doi: 10.4269/ajtmh.19-0666 [published Online First: 2020/07/04]
18. Daniels RF, Schaffner SF, Wenger EA, et al. Modeling malaria genomics reveals transmission decline and rebound in Senegal. *Proc Natl Acad Sci U S A* 2015;112(22):7067-72. doi: 10.1073/pnas.1505691112 [published Online First: 2015/05/06]
19. Daniels R, Chang HH, Sene PD, et al. Genetic surveillance detects both clonal and epidemic transmission of malaria following enhanced intervention in Senegal. *PLoS One* 2013;8(4):e60780. doi: 10.1371/journal.pone.0060780 [published Online First: 2013/04/18]
20. Nkhoma SC, Nair S, Al-Saai S, et al. Population genetic correlates of declining transmission in a human pathogen. *Mol Ecol* 2013;22(2):273-85. doi: 10.1111/mec.12099 [published Online First: 2012/11/06]
21. Sisya TJ, Kamn'gona RM, Vareta JA, et al. Subtle changes in Plasmodium falciparum infection complexity following enhanced intervention in Malawi. *Acta Trop* 2015;142:108-14. doi: 10.1016/j.actatropica.2014.11.008 [published Online First: 2014/12/03]

For peer review only - http://bmjopen.bmj.com/site/about/guidelines.xhtml

22. Daniels R, Volkman SK, Milner DA, et al. A general SNP-based molecular barcode for Plasmodium falciparum identification and tracking. *Malar J* 2008;7:223. doi: 10.1186/1475-2875-7-223 [published Online First: 2008/10/31]
23. Chang HH, Worby CJ, Yeka A, et al. THE REAL McCOIL: A method for the concurrent estimation of the complexity of infection and SNP allele frequency for malaria parasites. *PLoS Comput Biol* 2017;13(1):e1005348. doi: 10.1371/journal.pcbi.1005348 [published Online First: 2017/01/27]
24. Mayor A, Menendez C, Walker PGT. Targeting Pregnant Women for Malaria Surveillance. *Trends Parasitol* 2019 doi: 10.1016/j.pt.2019.07.005 [published Online First: 2019/08/10]
25. Kwesigabo G, Killewo JZ, Urassa W, et al. Monitoring of HIV-1 infection prevalence and trends in the general population using pregnant women as a sentinel population: 9 years experience from the Kagera region of Tanzania. *J Acquir Immune Defic Syndr* 2000;23(5):410-7.
26. Korenromp EL, Mahiane SG, Nagelkerke N, et al. Syphilis prevalence trends in adult women in 132 countries - estimations using the Spectrum Sexually Transmitted Infections model. *Sci Rep* 2018;8(1):11503. doi: 10.1038/s41598-018-29805-9 [published Online First: 2018/08/02]
27. Galatas B, Bassat Q, Mayor A. Malaria Parasites in the Asymptomatic: Looking for the Hay in the Haystack. *Trends Parasitol* 2016;32(4):296-308. doi: 10.1016/j.pt.2015.11.015
28. Hellewell J, Walker P, Ghani A, et al. Using ante-natal clinic prevalence data to monitor temporal changes in malaria incidence in a humanitarian setting in the Democratic Republic of Congo. *Malar J* 2018;17(1):312. doi: 10.1186/s12936-018-2460-9 [published Online First: 2018/08/31]
29. Mayor A, Bardaji A, Macete E, et al. Changing Trends in P. falciparum Burden, Immunity, and Disease in Pregnancy. *N Engl J Med* 2015;373(17):1607-17. doi: 10.1056/NEJMoa1406459 [published Online First: 2015/10/22]
30. Kitojo C, Gutman JR, Chacky F, et al. Estimating malaria burden among pregnant women using data from antenatal care centres in Tanzania: a population-based study. *Lancet Glob Health* 2019;7(12):e1695-e705. doi: 10.1016/S2214-109X(19)30405-X [published Online First: 2019/11/12]
31. van Eijk A, Hill J, Snow R, et al. The relationship between the prevalence of malaria in pregnant women and the prevalence of malaria in children and non-pregnant women in Sub-Saharan Africa. *American Society of Tropical Medicine and Hygiene Meeting* 2014;91(Suppl 5):287.
32. Willilo RA, Molteni F, Mandike R, et al. Pregnant women and infants as sentinel populations to monitor prevalence of malaria: results of pilot study in Lake Zone of Tanzania. *Malar J* 2016;15(1):392. doi: 10.1186/s12936-016-1441-0
33. Organization WH. World Malaria Report 2021. 2021.
34. WHO. Surveillance template protocol for pfhrp2/pfhrp3 gene deletions. Geneva: World Health Organization 2020 [
35. Chidimatembue A, Svigel SS, Mayor A, et al. Molecular surveillance for polymorphisms associated with artemisinin-based combination therapy resistance in Plasmodium falciparum isolates collected in Mozambique, 2018. *Malar J* 2021;20(1):398. doi: 10.1186/s12936-021-03930-9 [published Online First: 20211012]
36. Nhama A, Nhamussua L, Macete E, et al. In vivo efficacy and safety of artemether-lumefantrine and amodiaquine-artesunate for uncomplicated Plasmodium falciparum malaria in Mozambique, 2018. *Malar J* 2021;20(1):390. doi: 10.1186/s12936-021-03922-9 [published Online First: 20211002]
37. Ariey F, Witkowski B, Amaratunga C, et al. A molecular marker of artemisinin-resistant Plasmodium falciparum malaria. *Nature* 2014;505(7481):50-5. doi: 10.1038/nature12876 [published Online First: 20131218]
38. Venkatesan M, Alifrangis M, Roper C, et al. Monitoring antifolate resistance in intermittent preventive therapy for malaria. *Trends Parasitol* 2013;29(10):497-504. doi: 10.1016/j.pt.2013.07.008 [published Online First: 20130812]
39. Fidock DA, Nomura T, Talley AK, et al. Mutations in the P. falciparum digestive vacuole transmembrane protein PfCRT and evidence for their role in chloroquine resistance. *Mol Cell* 2000;6(4):861-71. doi: 10.1016/s1097-2765(05)00077-8
40. Tessema SK, Hathaway NJ, Teyssier NB, et al. Sensitive, highly multiplexed sequencing of microhaplotypes from the Plasmodium falciparum heterozygome. *J Infect Dis* 2020 doi: 10.1093/infdis/jiaa527 [published Online First: 2020/08/26]
41. Harris PA, Taylor R, Thielke R, et al. Research electronic data capture (REDCap)--a metadata-driven methodology and workflow process for providing translational research informatics support. *J Biomed Inform* 2009;42(2):377-81. doi: 10.1016/j.jbi.2008.08.010
42. Consortium M. Strengthening malaria surveillance for data-driven decision making in Mozambique 2019 [Available from: https://www.malariaconsortium.org/resources/publications/1255/strengthening-malaria-surveillance-for-data-driven-decision-making-in-mozambique.
43. Organization WH. Master protocol for surveillance of pfhrp2/3 deletions and biobanking to support future research. Geneva: WHO; 2020 [Available from: https://apps.who.int/iris/bitstream/handle/10665/331197/9789240002050-eng.pdf?ua=1.

For peer review only - http://bmjopen.bmj.com/site/about/guidelines.xhtml

44. Ruiz-Arenas C, Caceres A, Lopez-Sanchez M, et al. scoreInvHap: Inversion genotyping for genome-wide association studies. *PLoS Genet* 2019;15(7):e1008203. doi: 10.1371/journal.pgen.1008203 [published Online First: 2019/07/04]
45. Eckhoff P. Mathematical models of within-host and transmission dynamics to determine effects of malaria interventions in a variety of transmission settings. *Am J Trop Med Hyg* 2013;88(5):817-27. doi: 10.4269/ajtmh.12-0007 [published Online First: 2013/04/17]
46. Amimo F, Lambert B, Magit A, et al. Plasmodium falciparum resistance to sulfadoxine-pyrimethamine in Africa: a systematic analysis of national trends. *BMJ Glob Health* 2020;5(11) doi: 10.1136/bmjgh-2020-003217
47. Some AF, Zongo I, Compaore YD, et al. Selection of drug resistance-mediating Plasmodium falciparum genetic polymorphisms by seasonal malaria chemoprevention in Burkina Faso. *Antimicrob Agents Chemother* 2014;58(7):3660-5. doi: 10.1128/AAC.02406-14 [published Online First: 2014/04/16]
48. Neafsey DE, Volkman SK. Malaria Genomics in the Era of Eradication. *Cold Spring Harb Perspect Med* 2017;7(8) doi: 10.1101/cshperspect.a025544 [published Online First: 2017/04/09]
49. Wesolowski A, Taylor AR, Chang HH, et al. Mapping malaria by combining parasite genomic and epidemiologic data. *BMC Med* 2018;16(1):190. doi: 10.1186/s12916-018-1181-9 [published Online First: 20181018]
50. Dudas G, Carvalho LM, Bedford T, et al. Virus genomes reveal factors that spread and sustained the Ebola epidemic. *Nature* 2017;544(7650):309-15. doi: 10.1038/nature22040 [published Online First: 20170412]
51. Lemey P, Rambaut A, Bedford T, et al. Unifying viral genetics and human transportation data to predict the global transmission dynamics of human influenza H3N2. *PLoS Pathog* 2014;10(2):e1003932. doi: 10.1371/journal.ppat.1003932 [published Online First: 20140220]
52. Saravanan KA, Panigrahi M, Kumar H, et al. Role of genomics in combating COVID-19 pandemic. *Gene* 2022;823:146387. doi: 10.1016/j.gene.2022.146387 [published Online First: 20220304]
53. Volkman SK, Neafsey DE, Schaffner SF, et al. Harnessing genomics and genome biology to understand malaria biology. *Nat Rev Genet* 2012;13(5):315-28. doi: 10.1038/nrg3187 [published Online First: 2012/04/13]
54. Galinsky K, Valim C, Salmier A, et al. COIL: a methodology for evaluating malarial complexity of infection using likelihood from single nucleotide polymorphism data. *Malar J* 2015;14:4. doi: 10.1186/1475-2875-14-4 [published Online First: 2015/01/21]
55. Kateera F, Nsobya SL, Tukwasibwe S, et al. Malaria case clinical profiles and Plasmodium falciparum parasite genetic diversity: a cross sectional survey at two sites of different malaria transmission intensities in Rwanda. *Malar J* 2016;15:237. doi: 10.1186/s12936-016-1287-5 [published Online First: 2016/04/27]
56. Mohd Abd Razak MR, Sastu UR, Norahmad NA, et al. Genetic Diversity of Plasmodium falciparum Populations in Malaria Declining Areas of Sabah, East Malaysia. *PLoS One* 2016;11(3):e0152415. doi: 10.1371/journal.pone.0152415 [published Online First: 2016/03/31]
57. Nabet C, Doumbo S, Jeddi F, et al. Genetic diversity of Plasmodium falciparum in human malaria cases in Mali. *Malar J* 2016;15:353. doi: 10.1186/s12936-016-1397-0 [published Online First: 2016/07/13]
58. Vafa M, Troye-Blomberg M, Anchang J, et al. Multiplicity of Plasmodium falciparum infection in asymptomatic children in Senegal: relation to transmission, age and erythrocyte variants. *Malar J* 2008;7:17. doi: 10.1186/1475-2875-7-17 [published Online First: 2008/01/25]
59. Chang HH, Park DJ, Galinsky KJ, et al. Genomic sequencing of Plasmodium falciparum malaria parasites from Senegal reveals the demographic history of the population. *Mol Biol Evol* 2012;29(11):3427-39. doi: 10.1093/molbev/mss161 [published Online First: 2012/06/27]
60. Niang M, Thiam LG, Loucoubar C, et al. Spatio-temporal analysis of the genetic diversity and complexity of Plasmodium falciparum infections in Kedougou, southeastern Senegal. *Parasit Vectors* 2017;10(1):33. doi: 10.1186/s13071-017-1976-0 [published Online First: 2017/01/21]
61. Volkman SK, Ndiaye D, Diakite M, et al. Application of genomics to field investigations of malaria by the international centers of excellence for malaria research. *Acta Trop* 2012;121(3):324-32. doi: 10.1016/j.actatropica.2011.12.002 [published Online First: 2011/12/21]
62. Zhu SJ, Almagro-Garcia J, McVean G. Deconvolution of multiple infections in Plasmodium falciparum from high throughput sequencing data. *Bioinformatics* 2018;34(1):9-15. doi: 10.1093/bioinformatics/btx530 [published Online First: 2017/09/30]
63. Cerqueira GC, Cheeseman IH, Schaffner SF, et al. Longitudinal genomic surveillance of Plasmodium falciparum malaria parasites reveals complex genomic architecture of emerging artemisinin resistance. *Genome Biol* 2017;18(1):78. doi: 10.1186/s13059-017-1204-4 [published Online First: 2017/04/30]
64. Baetscher DS, Clemento AJ, Ng TC, et al. Microhaplotypes provide increased power from short-read DNA sequences for relationship inference. *Mol Ecol Resour* 2018;18(2):296-305. doi: 10.1111/1755-0998.12737 [published Online First: 2017/11/17]

For peer review only

65. Kidd KK, Pakstis AJ, Speed WC, et al. Current sequencing technology makes microhaplotypes a powerful new type of genetic marker for forensics. *Forensic Sci Int Genet* 2014;12:215-24. doi: 10.1016/j.fsigen.2014.06.014 [published Online First: 2014/07/20]
66. Bennett L, Oldoni F, Long K, et al. Mixture deconvolution by massively parallel sequencing of microhaplotypes. *Int J Legal Med* 2019;133(3):719-29. doi: 10.1007/s00414-019-02010-7 [published Online First: 2019/02/14]
67. Kidd KK, Speed WC. Criteria for selecting microhaplotypes: mixture detection and deconvolution. *Investig Genet* 2015;6:1. doi: 10.1186/s13323-014-0018-3 [published Online First: 2015/03/10]
68. Henden L, Lee S, Mueller I, et al. Identity-by-descent analyses for measuring population dynamics and selection in recombining pathogens. *PLoS Genet* 2018;14(5):e1007279. doi: 10.1371/journal.pgen.1007279 [published Online First: 2018/05/24]
69. Lawson DJ, Hellenthal G, Myers S, et al. Inference of population structure using dense haplotype data. *PLoS Genet* 2012;8(1):e1002453. doi: 10.1371/journal.pgen.1002453 [published Online First: 2012/02/01]
70. Libbrecht MW, Noble WS. Machine learning applications in genetics and genomics. *Nat Rev Genet* 2015;16(6):321-32. doi: 10.1038/nrg3920 [published Online First: 2015/05/08]
71. Moonasar D, Maharaj R, Kunene S, et al. Towards malaria elimination in the MOSASWA (Mozambique, South Africa and Swaziland) region. *Malar J* 2016;15(1):419. doi: 10.1186/s12936-016-1470-8 [published Online First: 2016/08/20]
72. Lover AA, Harvard KE, Lindawson AE, et al. Regional initiatives for malaria elimination: Building and maintaining partnerships. *PLoS Med* 2017;14(10):e1002401. doi: 10.1371/journal.pmed.1002401 [published Online First: 20171005]
73. de Vries J, Munung SN, Matimba A, et al. Regulation of genomic and biobanking research in Africa: a content analysis of ethics guidelines, policies and procedures from 22 African countries. *BMC Med Ethics* 2017;18(1):8. doi: 10.1186/s12910-016-0165-6 [published Online First: 20170202]

For peer review only

Figure legends

Figure 1. Malaria genomic use cases and National Malaria Control Program (NMCP) decisions.

The letter on the left (A-D) expresses the level of action described in the WHO Technical consultation on the role of parasite and anopheline genetics in malaria surveillance. A: Immediate action; B: Medium-term action; C: Long-term action. Arrows in color at the right express the research required for action in the medium and long-term (grey: not essential for action; green: immediate evidence; yellow: medium-term evidence). Abbreviations: ANC, antenatal care clinics; IPT, intermittent preventive treatment; MDA, mass drug administration; rfMDA, reactive focal MDA; SMC, seasonal malaria chemoprevention.

Figure 2. Low and medium-to-high transmission study districts targeted in the protocol.

Figure 3. Modelling approaches for malaria genomics.
Overview of the components of a joint malaria epidemiology-genetic model, that builds on the capabilities of two models previously developed at the Institute of Disease Modelling (a malaria genetic model calibrated to a longitudinal genetic study in Senegal and a disease transmission model calibrated with the Magude data).

For peer review only

For peer review only - http://bmjopen.bmj.com/site/about/guidelines.xhtml

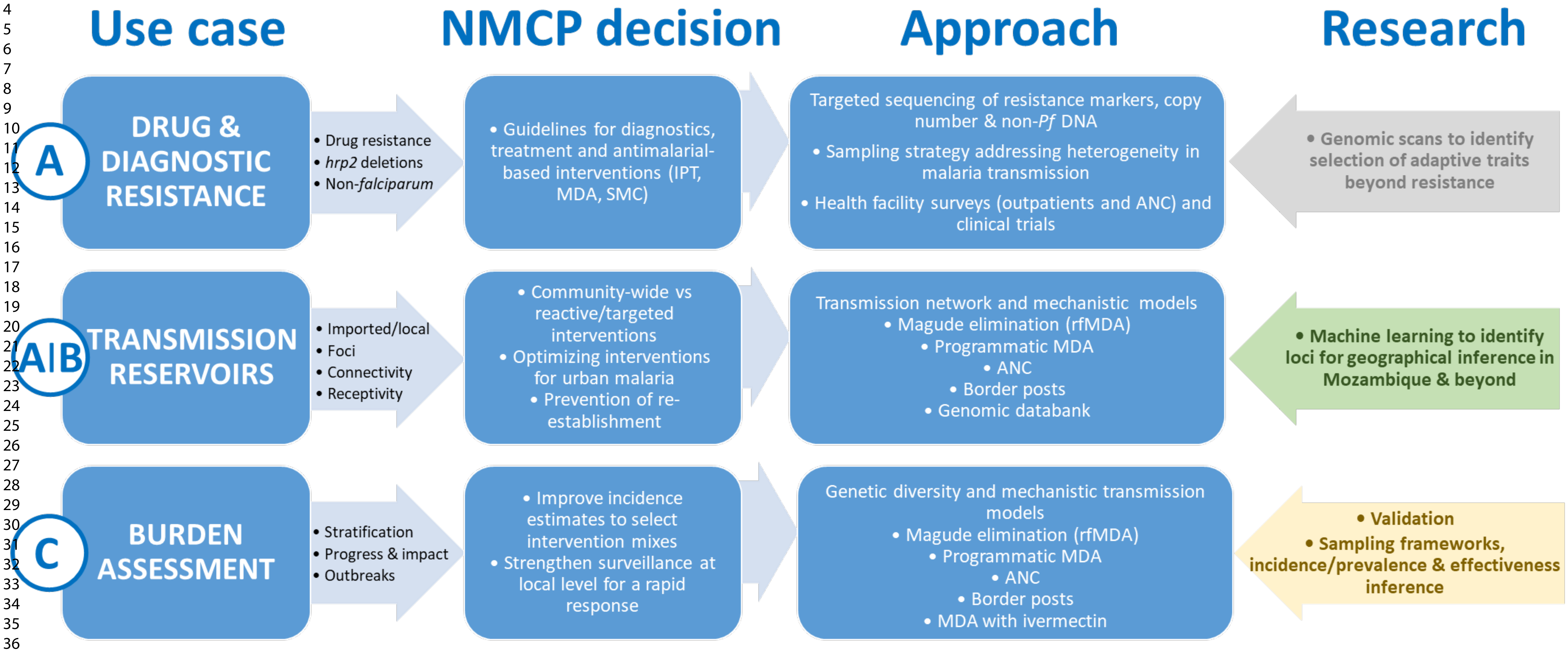
Use case
NMCP decision
Approach
Research
A
DRUG & DIAGNOSTIC RESISTANCE
• Drug resistance
• hrp2 deletions
• Non-falciparum
• Guidelines for diagnostics, treatment and antimalarial-based interventions (IPT, MDA, SMC)
Targeted sequencing of resistance markers, copy number & non-Pf DNA
• Sampling strategy addressing heterogeneity in malaria transmission
• Health facility surveys (outpatients and ANC) and clinical trials
• Genomic scans to identify selection of adaptive traits beyond resistance
A|B
TRANSMISSION RESERVOIRS
• Imported/local
• Foci
• Connectivity
• Receptivity
• Community-wide vs reactive/targeted interventions
• Optimizing interventions for urban malaria
• Prevention of re-establishment
Transmission network and mechanistic models
• Magude elimination (rfMDA)
• Programmatic MDA
• ANC
• Border posts
• Genomic databank
• Machine learning to identify loci for geographical inference in Mozambique & beyond
C
BURDEN ASSESSMENT
• Stratification
• Progress & impact
• Outbreaks
• Improve incidence estimates to select intervention mixes
• Strengthen surveillance at local level for a rapid response
Genetic diversity and mechanistic transmission models
• Magude elimination (rfMDA)
• Programmatic MDA
• ANC
• Border posts
• MDA with ivermectin
• Validation
• Sampling frameworks, incidence/prevalence & effectiveness inference

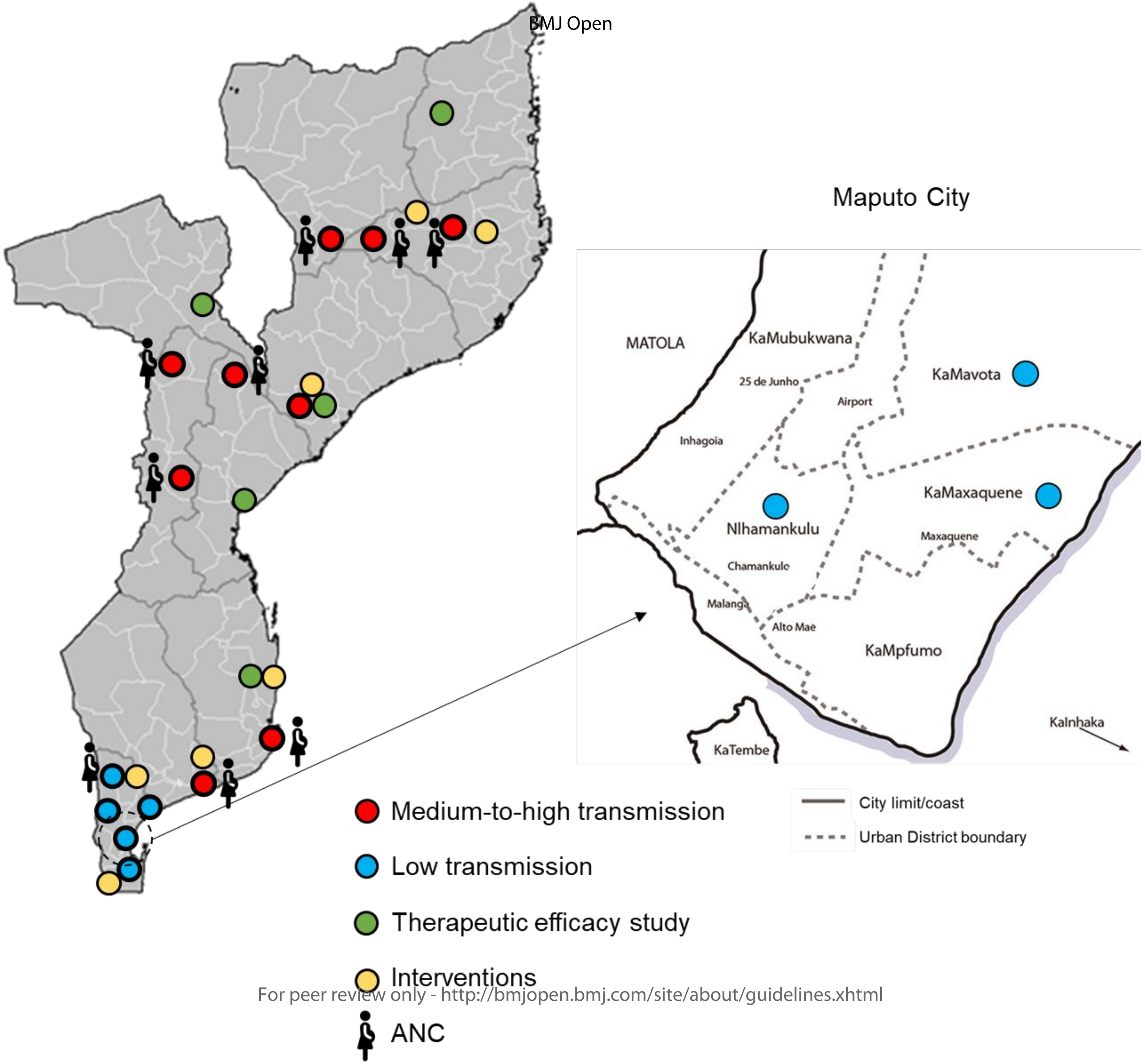
Maputo City
MATOLA
KaMubukwana
25 de Junho
Airport
KaMavota
Inhagoia
KaMaxaquene
Nlhamankulu
Maxaquene
Chamankulo
Malanga
Alto Mae
KaMpfumo
KaInhaka
KaTembe
City limit/coast
Urban District boundary
Medium-to-high transmission
Low transmission
Therapeutic efficacy study
Interventions
ANC

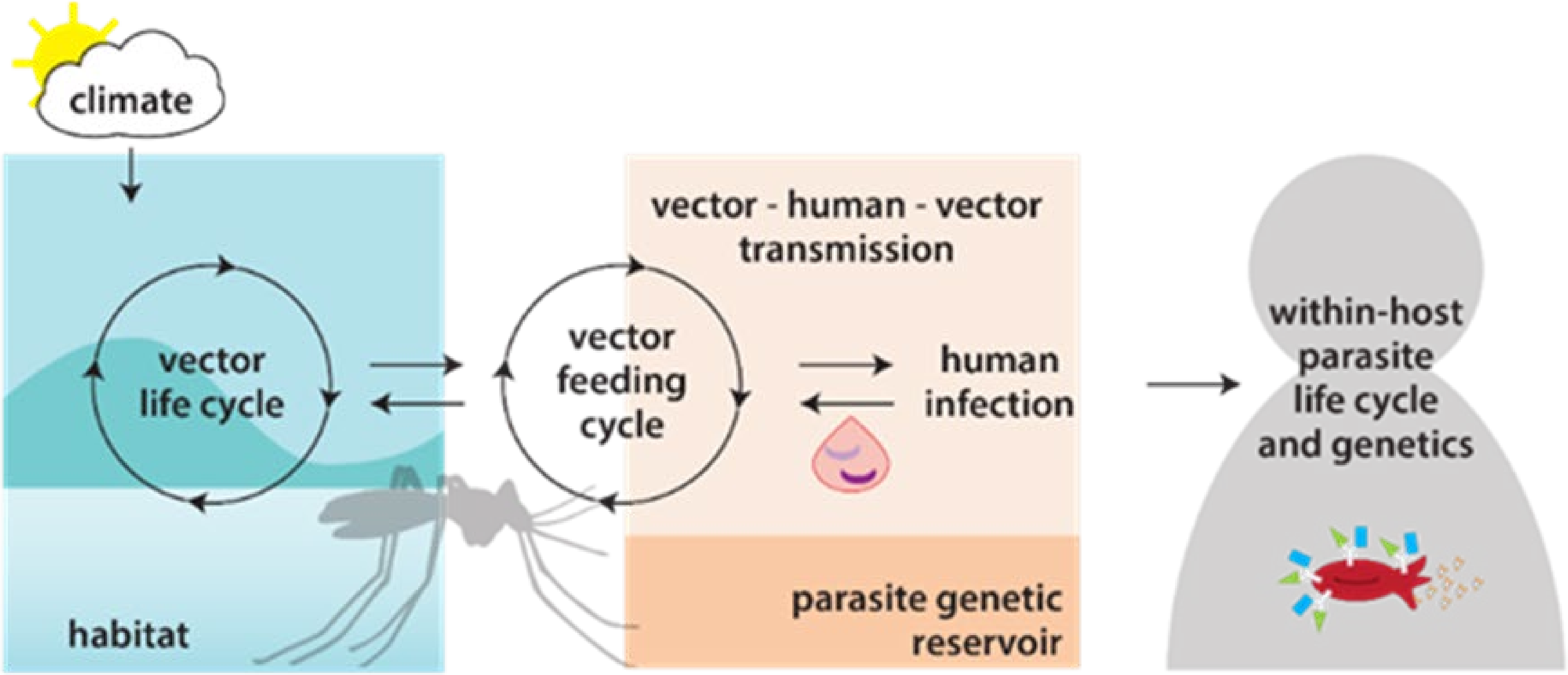
climate
vector
life cycle
habitat
vector
feeding
cycle
vector - human - vector
transmission
human
infection
parasite genetic
reservoir
within-host
parasite
life cycle
and genetics

Supplementary Table 1. Control measures for bool collection through fingerprick.

Potential Hazards	Likelihood	Consequences	Control Measures
Lancet stick injury	Rare	Possibility of infection (hep B or HIV)	Wear PPE and work slowly and carefully. The lancets for finger pricks are designed in such a way that they can only be used once, thereby minimising the possibility of cross contamination.
Incorrect blood collection procedure	Moderate	No blood drawn	Follow detailed SOPs for blood collection procedures for finger picks; dispose of all contaminated waste in the biohazard bags.
Haematoma	Rare	Bruising and painful lancet entry site	Follow correct collection procedures (SOPs), if unable to draw blood, withdraw the lancet and apply light pressure to the site. Do not attempt to withdraw blood at the same site again.
Fainting	Moderate	Subject may feel faint at the sight of blood	Lie the patient down and stay with them until they have recovered. Little sips of water and a wet towel applied to the forehead. Verbal communication throughout the procedure will reassure the subject.

For peer review only

Supplementary Table 2. Roles of each partner organization.

Organisation	Role
**National Malaria Control Programme**	Technical oversight of all research
**Malaria Consortium**	Development of the sampling protocol and ethical clearance<br>Training of data collectors<br>Field collection of blood samples and participant data<br>Transfer of samples and data to CISM<br>Creation of a surveillance dashboard<br>Transference of activities to NMCP
**Centro de Investigação em Saúde de Manhiça (CISM)**	Financial, organizational and overall coordination<br>Genetic analysis<br>Long-term storage of study samples<br>Analysis of data<br>Scientific/programmatic dissemination of results
**National Institute of Health (INS)**	Malaria Indicator and ANC Surveys in Inhambane, Gaza, Maputo and Maputo City<br>Sample analysis
**ISGlobal**	Supervision of epidemiological aspects<br>Technical support for sampling strategy, data collection and epidemiological analysis<br>Development of sequencing pipeline
**Institute for Disease Modelling**	Development and calibration of an epidemiological genomic model for Mozambique regions
**University of California San Francisco**	Development of sequencing and analytical pipelines<br>Development of transmission network model<br>Training and supervision of genetic and bioinformatics activities

For peer review only - http://bmjopen.bmj.com/site/about/guidelines.xhtml

Appendixes

Appendix 1. Information sheet and informed consent for participants over 18 years of age

Appendix 2. Information sheet and informed assent for minors aged between 12 and 17 years old

Appendix 3. Information sheet and informed consent for adult pregnant women

Appendix 4. Information sheet and informed assent for pregnant women between 12 and 18 years of age

Appendix 5. Questionnaire for Medium-high transmission area, children under 2-10 years old

Appendix 6. Questionnaire for Low transmission area, all ages

Appendix 7. Questionnaire for Pregnant women attending ANC clinic in medium-high transmission area

Appendix 8. Questionnaire for Pregnant women attending ANC clinic in low transmission area

Appendix 9. Procedures for the collection, handling and storage of dried blood samples on filter paper and rapid diagnostic tests (RDT).

Appendix 10. COVID-19 safety and research considerations

Appendix 11. Worksheet for Monitoring and Evaluation of Field Activities

For peer review only

Appendix 1. Information sheet and informed consent for participants over 18 years of age

PART I

INFORMATION SHEET AND INFORMED CONSENT FOR PARTICIPANTS OVER 18 YEARS OF AGE

Name of Affiliated Institutions

1. Manhiça Health Research Center (CISM), Manhiça, Mozambique
2. Malaria Consortium, Maputo, Mozambique
3. Barcelona Global Health Institute (ISGlobal), Barcelona, Spain
4. National Malaria Control Programme, Ministry of Health, Maputo, Mozambique
5. University of California, San Francisco, USA
6. Clinton Health Access Initiative, Boston, USA
7. Institute of Disease Modeling, Bill and Melinda Gates Foundation, Seattle, USA
8. Bill and Melinda Gates Foundation, Seattle, USA

Protocol title and version: "A prospective surveillance study to detect antimalarial drug resistance, gene deletions of diagnostic relevance and genetic diversity of *Plasmodium falciparum* in Mozambique," version number 7, 25 August 2021.

Name and affiliation of Principal Investigator(s): Baltazar Candrinho, National Malaria Control Programme, Ministry of Health, Maputo, Mozambique and Alfredo Mayor, Manhiça Health Research Center, Manhiça, Mozambique.

Study funder: Bill and Melinda Gates Foundation, USA

Introduction: The National Malaria Control Programme in partnership with Malaria Consortium and the Manhiça Health Research Center are conducting a study to analyse the genetics of malaria parasites to identify the best ways to control and/or eliminate this disease from the country.

Please read this form with care. This form provides important information about participating in this study. All the information which follows, discussed below, is to allow you to understand what the study involves and the steps that would need your collaboration, so that before becoming involved in the study, you can decide freely if you wish to participate.

You can take the time that you feel necessary to decide about your participation in this study. If you have questions about the study, or any part of this form, please ask us. If you decide to participate in this research, you will be asked to sign this form. One copy of the signed form will be provided to you for your records. If at any time you feel that you do not understand the information that is being provided, please do not hesitate to interrupt so that we can explain and clarify everything again.

After receiving your consent to participate, we will ask you some personal questions about your age, date of birth, recent illnesses, including history of fever, occupation, travel history, residence, use of insecticide treated mosquito nets or taking of antimalarial medications in the last month and then we will take a few drops of blood from your finger.

Rationale: Mozambique constitutes a main goal for the World Health Organization and partnership initiative, namely, Roll Back Malaria, to end malaria in the world. In this context, through involvement in regional malaria elimination initiatives, the use of molecular malaria surveillance data, as a complement to traditional surveillance information, can contribute to the elimination of malaria in Southern Mozambique and a reduction of the burden in the north of the country. However, there is a lack of malaria diagnostic and drug resistance data and other measures of the genetic diversity of the parasite that causes malaria in different transmission settings. Therefore, more evidence is needed to demonstrate the feasibility of using genetic data as a driver of the intensity of transmission in high transmission areas. Additionally, understanding the prevalence of diagnostic and drug resistance and genetic diversity will inform more appropriate and impactful interventions to reduce malaria morbidity and mortality in Mozambique. The integration of genetic data into routine

For peer review only - http://bmjopen.bmj.com/site/about/guidelines.xhtml

surveillance activities has the potential to increase knowledge for programmatic decision-making on the optimal combination of control and elimination measures in Mozambique.

Research objectives: Your participation in this study will help us to identify the prevalence of molecular markers of antimalarial resistance along with other genetic markers, which will inform the National Malaria Control Programme to support decision-making on the use of antimalarials and best strategies to control and eliminate malaria in the country.

Type of research/Intervention: This is prospective, operational surveillance research.

Selection of participants: You were invited to participate in this research because you are part of a group that is the focus of this study: **adults over 18 years of age** with malaria, confirmed by a rapid diagnostic test, living in this region.

Voluntary participation: It's your choice if you want to participate in this study or not. Refusing to participate or withdrawing your participation will not result in any penalty or loss of health benefits or services. You will continue to receive medical care if you choose not to participate in this study. Your decision will not change the care that you receive now or in the future. Participating in this study is your choice. If you decide to participate in this study, you can leave at any time without consequences. If you want to stop participating in the study, just let the research team know.

Procedures: We will take a few drops of blood from your finger and four drops will be placed on two small pieces of paper (filter paper), two drops on each paper. The filter papers containing four drops of blood each will be kept in the Health Unit and sent to Manhiça Health Research Center where the analysis will be done. If necessary, the filter papers may be sent to a laboratory located outside of Mozambique (specifically, the ISGlobal laboratory in Spain or the University of California, San Francisco laboratory, in the United States) for additional analysis and molecular characterisation of the malaria parasites (alleles related to antimalarial resistance as well as genetic composition and other molecular markers of relevance to malaria surveillance, both in the parasite and human host). The filter papers will be stored by the Manhiça Health Research Center for future human and parasite malaria molecular studies for a period of up to 10 years. In addition to drops of blood, all participants will also be asked about their age, date of birth, recent illnesses, including history of fever, occupation, travel history, residence, use of insecticide treated mosquito nets or antimalaria medication taking in the past 24/48 hours.

Risks, Discomfort and Inconvenience: You may feel a little pain or fear when your finger is pricked. The pain will dissipate within a few hours.

Benefits: There are no direct benefits for you to participate in this study. However, the findings generated from the study will inform the National Malaria Control Programme in decision-making about the use of antimalarials and the best strategies to control and eliminate malaria from the country.

Costs of Participation/Compensation: You will not receive any money or compensation to take part in this study.

Privacy: The data collected will be anonymous, however the data obtained in this study may be shared with collaborating partners: The National Malaria Control Programme, Malaria Consortium Mozambique, Manhiça Health Research Center, ISGlobal, Institute of Disease Modeling and the University of California, San Francisco, USA. In relation to the DNA sequences of the malaria parasite, or your personal data, these will be archived in an online database that can be shared with other scientists and researchers when the data are sent to scientific publications to report the results of this study.

Confidentiality: The information collected will be kept confidential and only the study team will have access to individuals' information. The results of the study will be published and made available so that other interested people can learn from our study, but confidential information will not be shared in any circumstance. Your data will be completely anonymised.

For peer review only - http://bmjopen.bmj.com/site/about/guidelines.xhtml

Sharing of results: Results from this research will be shared on open access platforms online, in public data repositories or directly in scientific publications, in order to facilitate further collaboration, enhance trust in the findings and goodwill among researchers. We will specifically focus on data sharing among other African countries in the region which are engaging in similar approaches to the molecular surveillance of malaria.

Whom to Contact (Investigators and Ethics Committee): in case of any of these situations:

- If your questions, concerns or complaints are not being addressed by the research team;
- If you are unable to contact the research team;
- If you would like to speak with someone who is not part of the research team;
- If you have questions about your rights as a research participant;
- If you wish to obtain information or provide information about this research; or
- If you think that the study has caused harm.

Please return to the Health Unit and speak with the workers involved in the study or contact the study focal person, assigned by Malaria Consortium Mozambique, Neide Canana on telephone number: 860450563, or you can contact her at: Malaria Consortium Mozambique, Sita Av. Lucas Elias Kumato nr. 118, Bairro da Sommerschield – Maputo City, Mozambique, or you can also contact Manhiça Health Research Center, located at: Street 12, Bairro Cambeve in Município da Manhiça Maputo Province, Mozambique, or by telephone: 21810002. In case you are not satisfied with the responses provided, you may also contact the National Bioethics for Health Committee, Ministry of Health, Mozambique on the numbers: 824066350/844693186.

Ethics Committee approval of this study: This study was approved by the Manhiça Health Research Center Institutional Bioethics Health Committee and the National Bioethics for Health Committee.

PART II
DECLARATION OF INFORMED CONSENT

Study Title: "A prospective surveillance study to detect antimalarial drug resistance, gene deletions of diagnostic relevance and genetic diversity of *Plasmodium falciparum* in Mozambique."

Declaration: I have read the information provided in this consent form, including the risks and possible benefits. All my questions about the research have been answered satisfactorily. I understand that I am free to withdraw from the study at any time without repercussions or loss of benefits to which I am entitled.

I give my consent to participate in this study.

INFORMED CONSENT
If there is any part of this consent form that you do not understand, ask the investigator before you sign.

I, ______________________ (Name of participant) give my voluntary consent to participate in the study: "A prospective surveillance study to detect antimalarial drug resistance, gene deletions of diagnostic relevance and genetic diversity of *Plasmodium falciparum* in Mozambique."

My questions have all been answered by _________________ (Name of researcher) in my own language. In case I have any other questions, I know that I can contact the study focal person assigned to Malaria Consortium and the National Bioethics for Health Committee through the contacts provided. I understand that I may withdraw my participation from the study, at any time for any reason, without any repercussions.

Do you allow your samples to be stored and used in future research? ▫ **Yes** ▫ **No**

I agree to take part in this study.

For peer review only - http://bmjopen.bmj.com/site/about/guidelines.xhtml

Signatures

Participant's fingerprint if they cannot sign

_____________________________ ____________
Signature of participant Date and time

____
Name of participant (in capital letters)

____
Signature of the person who explained consent

_________________________________ ______________
Name of the person who explained consent (in capital letters) Date and time

If the participant does not know how to read, an impartial witness must also sign this form:

____ ___________
Signature of the impartial witness Date and time

____
Name of the impartial witness (in capital letters)

For peer review only

Appendix 2. Information sheet and informed assent for minors aged between 12 and 17 years old.

PART I

INFORMATION SHEET AND INFORMED ASSENT FOR MINORS AGED BETWEEN 12 AND 17 YEARS OLD

Name of Affiliated Institutions

9. Manhiça Health Research Center (CISM), Manhiça, Mozambique
10. Malaria Consortium, Maputo, Mozambique
11. Barcelona Global Health Institute (ISGlobal), Barcelona, Spain
12. National Malaria Control Programme, Ministry of Health, Maputo, Mozambique
13. University of California, San Francisco, USA
14. Clinton Health Access Initiative, Boston, USA
15. Institute of Disease Modeling, Bill and Melinda Gates Foundation, Seattle, USA
16. Bill and Melinda Gates Foundation, Seattle, USA

Protocol title and version: "A prospective surveillance study to detect antimalarial drug resistance, gene deletions of diagnostic relevance and genetic diversity of *Plasmodium falciparum* in Mozambique," version number 7, 25 August 2021.

Name and affiliation of Principal Investigator(s): Baltazar Candrinho, National Malaria Control Programme, Ministry of Health, Maputo, Mozambique and Alfredo Mayor, Manhiça Health Research Center, Manhiça, Mozambique.

Study funder: Bill and Melinda Gates Foundation, USA

Introduction: The National Malaria Control Programme in partnership with Malaria Consortium and the Manhiça Health Research Center are conducting a study to analyse the genetics of malaria parasites to identify the best ways to control and/or eliminate this disease from the country.

Please read this form with care. This form provides important information about participating in this study. All the information which follows, discussed below, is to allow you to understand what the study involves and the steps that would need your collaboration, so that before becoming involved in the study, you can decide freely if you wish to participate.

You can take the time that you feel necessary to decide about your participation in this study. If you have questions about the study, or any part of this form, please ask us. If you decide to participate in this research, you will be asked to sign this form. One copy of the signed form will be provided to you for your records. If at any time you feel that you do not understand the information that is being provided, please do not hesitate to interrupt so that we can explain and clarify everything again.

After receiving your consent to participate, we will ask you some personal questions about your age, date of birth, recent illnesses, including history of fever, occupation, travel history, residence, use of insecticide treated mosquito nets or taking of antimalarial medications in the last month and then we will take a few drops of blood from your finger.

Rationale: Mozambique constitutes a main goal for the World Health Organization and partnership initiative, namely, Roll Back Malaria, to end malaria in the world. In this context, through involvement in regional malaria elimination initiatives, the use of molecular malaria surveillance data, as a complement to traditional surveillance information, can contribute to the elimination of malaria in Southern Mozambique and a reduction of the burden in the north of the country. However, there is a lack of malaria diagnostic and drug resistance data and other measures of the genetic diversity of the parasite that causes malaria in different transmission settings. Therefore, more evidence is needed to demonstrate the feasibility of using genetic data as a driver of the intensity of transmission in high transmission areas. Additionally, understanding the prevalence of diagnostic and drug resistance and genetic diversity will inform more appropriate and impactful interventions

For peer review only - http://bmjopen.bmj.com/site/about/guidelines.xhtml

to reduce malaria morbidity and mortality in Mozambique. The integration of genetic data into routine surveillance activities has the potential to increase knowledge for programmatic decision-making on the optimal combination of control and elimination measures in Mozambique.

Research objectives: Your participation in this study will help us to identify the prevalence of molecular markers of antimalarial resistance along with other genetic markers, which will inform the National Malaria Control Programme to support decision-making on the use of antimalarials and best strategies to control and eliminate malaria in the country.

Type of research/Intervention: This is prospective, operational surveillance research.

Selection of participants: You were invited to participate in this research because you are part of a group that is the focus of this study: **minors aged between 0 and 18 years of age** with malaria, confirmed by a rapid diagnostic test, living in this region.

Voluntary participation: It's your choice if you want to participate in this study or not. Refusing to participate or withdrawing your participation will not result in any penalty or loss of health benefits or services. You will continue to receive medical care if you choose not to participate in this study. Your decision will not change the care that you receive now or in the future. Participating in this study is your choice. If you decide to participate in this study, you can leave at any time without consequences. If you want to stop participating in the study, just let the research team know.

Procedures: We will take a few drops of blood from your finger and four drops will be placed on two small pieces of paper (filter paper), two drops on each paper. The filter papers containing four drops of blood each will be kept in the Health Unit and sent to Manhiça Health Research Center where the analysis will be done. If necessary, the filter papers may be sent to a laboratory located outside of Mozambique (specifically, the ISGlobal laboratory in Spain or the University of California, San Francisco laboratory, in the United States) for additional analysis and molecular characterisation of the malaria parasites (alleles related to antimalarial resistance as well as genetic composition and other molecular markers of relevance to malaria surveillance, both in the parasite and human host). The filter papers will be stored by the Manhiça Health Research Center for future human and parasite malaria molecular studies for a period of up to 10 years. In addition to drops of blood, all participants will also be asked about their age, date of birth, recent illnesses, including history of fever, occupation, travel history, residence, use of insecticide treated mosquito nets or antimalaria medication taking in the past 24/48 hours.

Risks, Discomfort and Inconvenience: You may feel a little pain or fear when your finger is pricked. The pain will dissipate within a few hours.

Benefits: There are no direct benefits for you to participate in this study. However, the findings generated from the study will inform the National Malaria Control Programme in decision-making about the use of antimalarials and the best strategies to control and eliminate malaria from the country.

Costs of Participation/Compensation: You will not receive any money or compensation to take part in this study.

Privacy: The data collected will be anonymous, however the data obtained in this study may be shared with collaborating partners: the National Malaria Control Programme, Malaria Consortium Mozambique, Manhiça Health Research Center, ISGlobal, Institute of Disease Modeling and the University of California, San Francisco, USA. In relation to the DNA sequences of the malaria parasite, or your personal data, these will be archived in an online database that can be shared with other scientists and researchers when the data are sent to scientific publications to report the results of this study.

Confidentiality: The information collected will be kept confidential and only the study team will have access to individuals' information. The results of the study will be published and made available so that other interested

For peer review only

people can learn from our study, but confidential information will not be shared in any circumstance. Your data will be completely anonymised.

Sharing of results: Results from this research will be shared on open access platforms online, in public data repositories or directly in scientific publications, in order to facilitate further collaboration, enhance trust in the findings and goodwill among researchers. We will specifically focus on data sharing among other African countries in the region which are engaging in similar approaches to the molecular surveillance of malaria.

Whom to Contact (Investigators and Ethics Committee): in case of any of these situations:

- If your questions, concerns or complaints are not being addressed by the research team;
- If you are unable to contact the research team;
- If you would like to speak with someone who is not part of the research team;
- If you have questions about your rights as a research participant;
- If you wish to obtain information or provide information about this research; or
- If you think that the study has caused harm.

Please return to the Health Unit and speak with the workers involved in the study or contact the study focal person, assigned by Malaria Consortium Mozambique, Neide Canana on telephone number: 860450563, or you can contact her at: Malaria Consortium Mozambique, Sita Av. Lucas Elias Kumato nr. 118, Bairro da Sommerschield – Maputo City, Mozambique, or you can also contact Manhiça Health Research Center, located at: Street 12, Bairro Cambeve in Município da Manhiça Maputo Province, Mozambique, or by telephone: 21810002. In case you are not satisfied with the responses provided, you may also contact the National Bioethics for Health Committee, Ministry of Health, Mozambique on the numbers: 824066350/844693186.

Ethics Committee approval of this study: This study was approved by the Manhiça Health Research Center Institutional Bioethics Health Committee and the National Bioethics for Health Committee.

PART II

DECLARATION OF ASSENT

Study Title: "A prospective surveillance study to detect antimalarial drug resistance, gene deletions of diagnostic relevance and genetic diversity of *Plasmodium falciparum* in Mozambique."

Declaration: I have read the information provided in this assent form, including the risks and possible benefits. All of my questions about the research have been answered satisfactorily. I understand that I am free to withdraw from the study at any time without repercussions or loss of benefits to which I am entitled.

I give my assent to participate in this study.

INFORMED ASSENT

If there is any part of this assent form that you do not understand, ask the investigator before you sign.

I, ______________________ (Name of participant) give my voluntary assent to participate in the study: "A prospective surveillance study to detect antimalarial drug resistance, gene deletions of diagnostic relevance and genetic diversity of *Plasmodium falciparum* in Mozambique."

My questions have all been answered by __________________ (Name of researcher) in my own language. In case I have any other questions, I know that I can contact the study focal person assigned to Malaria Consortium and the National Bioethics for Health Committee through the contacts provided. I understand that I may withdraw my participation from the study, at any time for any reason, without any repercussions.

For peer review only - http://bmjopen.bmj.com/site/about/guidelines.xhtml

Do you allow your samples to be stored and used in future research? □ **Yes** □ **No**

I agree to take part in this study.

Signatures

Minor's fingerprint if they cannot sign

______________________ ______________
Signature of the minor Date and time

____
Minor's name (in capital letters)

____
Signature of the person who explained assent

______________________________ ______________
Name of the person who explained assent (in capital letters) Date and time

If the minor does not know how to read, an impartial witness must also sign this form:

______________________________ ______________
Signature of the impartial witness Date and time

____
Name of the impartial witness (in capital letters)

For peer review only

PART III

INFORMATION SHEET AND INFORMED CONSENT FOR PARENTS/GUARDIANS OF MINOR PARTICIPANTS LESS THAN 18 YEARS OF AGE

Name of Affiliated Institutions

17. Manhiça Health Research Center (CISM), Manhiça, Mozambique
18. Malaria Consortium, Maputo, Mozambique
19. Barcelona Global Health Institute (ISGlobal), Barcelona, Spain
20. National Malaria Control Programme, Ministry of Health, Maputo, Mozambique
21. University of California, San Francisco, USA
22. Clinton Health Access Initiative, Boston, USA
23. Institute of Disease Modeling, Bill and Melinda Gates Foundation, Seattle, USA
24. Bill and Melinda Gates Foundation, Seattle, USA

Protocol title and version: "A prospective surveillance study to detect antimalarial drug resistance, gene deletions of diagnostic relevance and genetic diversity of *Plasmodium falciparum* in Mozambique," version number 7, 25 August 2021.

Name and affiliation of Principal Investigator(s): Baltazar Candrinho, National Malaria Control Programme, Ministry of Health, Maputo, Mozambique and Alfredo Mayor, Manhiça Health Research Center, Manhiça, Mozambique.

Study funder: Bill and Melinda Gates Foundation, USA

Introduction: The National Malaria Control Programme in partnership with Malaria Consortium and the Manhiça Health Research Center are conducting a study to analyse the genetics of malaria parasites to identify the best ways to control and/or eliminate this disease from the country.

Please read this form with care. This form provides important information about participating in this study. All the information which follows, discussed below, is to allow you to understand what the study involves and the steps that would need your collaboration, so that before becoming involved in the study, you can decide freely if you wish for your child to participate.

You can take the time that you feel necessary to decide about your child's participation in this study. If you have questions about the study, or any part of this form, please ask us. If you decide for your child to participate in this research, you will be asked to sign this form. One copy of the signed form will be provided to you for your records. If at any time you feel that you do not understand the information that is being provided, please do not hesitate to interrupt so that we can explain and clarify everything again.

After receiving your consent for your child to participate, we will ask them some personal questions about their age, date of birth, recent illnesses, including history of fever, occupation, travel history, residence, use of insecticide treated mosquito nets or taking of antimalarial medications in the last month and then we will take a few drops of blood from their finger.

Rationale: Mozambique constitutes a main goal for the World Health Organization and partnership initiative, namely, Roll Back Malaria, to end malaria in the world. In this context, through involvement in regional malaria elimination initiatives, the use of molecular malaria surveillance data, as a complement to traditional surveillance information, can contribute to the elimination of malaria in Southern Mozambique and a reduction of the burden in the north of the country. However, there is a lack of malaria diagnostic and drug resistance data and other measures of the genetic diversity of the parasite that causes malaria in different transmission settings. Therefore, more evidence is needed to demonstrate the feasibility of using genetic data as a driver of the intensity of transmission in high transmission areas. Additionally, understanding the prevalence of diagnostic and drug resistance and genetic diversity will inform more appropriate and impactful interventions to reduce malaria morbidity and mortality in Mozambique. The integration of genetic data into routine surveillance activities has the potential to increase knowledge for programmatic decision-making on the optimal combination of control and elimination measures in Mozambique.

Research objectives: Your child's participation in this study will help us to identify the prevalence of molecular markers of antimalarial resistance along with other genetic markers, which will inform the National Malaria Control Programme to support decision-making on the use of antimalarials and best strategies to control and eliminate malaria in the country.

Type of research/Intervention: This is prospective, operational surveillance research.

Selection of participants: Your child was invited to participate in this research because they are part of a group that is the focus of this study: **minors aged between 0 and 18 years of age** with malaria, confirmed by a rapid diagnostic test, living in this region.

Voluntary participation: It's your and your child's choice if you want them to participate in this study or not. Refusing to participate or withdrawing their participation will not result in any penalty or loss of health benefits or services. Your child will continue to receive medical care if you choose for them not to participate in this study. Your/their decision will not change the care that they receive now or in the future. Participating in this study is your/their choice. If you decide for your child to participate in this study, they can leave at any time without consequences. If they want to stop participating in the study, just let the research team know.

Procedures: We will take a few drops of blood from your child's finger and four drops will be placed on two small pieces of paper (filter paper), two drops on each paper. The filter papers containing four drops of blood each will be kept in the Health Unit and sent to Manhiça Health Research Center where the analysis will be done. If necessary, the filter papers may be sent to a laboratory located outside of Mozambique (specifically, the ISGlobal laboratory in Spain or the University of California, San Francisco laboratory, in the United States) for additional analysis and molecular characterisation of the malaria parasites (alleles related to antimalarial resistance as well as genetic composition and other molecular markers of relevance to malaria surveillance, both in the parasite and human host). The filter papers will be stored by the Manhiça Health Research Center

For peer review only - http://bmjopen.bmj.com/site/about/guidelines.xhtml

for future human and parasite malaria molecular studies for a period of up to 10 years. In addition to drops of blood, all participants will also be asked about their age, date of birth, recent illnesses, including history of fever, occupation, travel history, residence, use of insecticide treated mosquito nets or antimalaria medication taking in the past 24/48 hours.

Risks, Discomfort and Inconvenience: Your child may feel a little pain or fear when their finger is pricked. The pain will dissipate within a few hours.

Benefits: There are no direct benefits for your child to participate in this study. However, the findings generated from the study will inform the National Malaria Control Programme in decision-making about the use of antimalarials and the best strategies to control and eliminate malaria from the country.

Costs of Participation/Compensation: You will not receive any money or compensation for your child to take part in this study.

Privacy: The data collected will be anonymous, however the data obtained in this study may be shared with collaborating partners: the National Malaria Control Programme, Malaria Consortium Mozambique, Manhiça Health Research Center, ISGlobal, Institute of Disease Modeling and the University of California, San Francisco, USA. In relation to the DNA sequences of the malaria parasite, or your child's personal data, these will be archived in an online database that can be shared with other scientists and researchers when the data are sent to scientific publications to report the results of this study.

Confidentiality: The information collected will be kept confidential and only the study team will have access to individuals' information. The results of the study will be published and made available so that other interested people can learn from our study, but confidential information will not be shared in any circumstance. Your child's data will be completely anonymised.

Sharing of results: Results from this research will be shared on open access platforms online, in public data repositories or directly in scientific publications, in order to facilitate further collaboration, enhance trust in the findings and goodwill among researchers. We will specifically focus on data sharing among other African countries in the region which are engaging in similar approaches to the molecular surveillance of malaria.

Whom to Contact (Investigators and Ethics Committee): in case of any of these situations:

- If your questions, concerns or complaints are not being addressed by the research team;
- If you are unable to contact the research team;
- If you would like to speak with someone who is not part of the research team;
- If you have questions about your rights as a research participant;
- If you wish to obtain information or provide information about this research; or
- If you think that the study has caused harm.

Please return to the Health Unit and speak with the workers involved in the study or contact the study focal person, assigned by Malaria Consortium Mozambique, Neide Canana on telephone number: 860450563, or you can contact her at: Malaria Consortium Mozambique, Sita Av. Lucas Elias Kumato nr. 118, Bairro da Sommerschield – Maputo City, Mozambique, or you can also contact Manhiça Health Research Center, located at: Street 12, Bairro Cambeve in Município da Manhiça Maputo Province, Mozambique, or by telephone: 21810002. In case you are not satisfied with the responses provided, you may also contact the National Bioethics for Health Committee, Ministry of Health, Mozambique on the numbers: 824066350/844693186.

Ethics Committee approval of this study: This study was approved by the Manhiça Health Research Center Institutional Bioethics Health Committee and the National Bioethics for Health Committee.

PART IV

For peer review only - http://bmjopen.bmj.com/site/about/guidelines.xhtml
For peer review only

DECLARATION OF INFORMED CONSENT

Study Title: “A prospective surveillance study to detect antimalarial drug resistance, gene deletions of diagnostic relevance and genetic diversity of *Plasmodium falciparum* in Mozambique.”

Declaration: I have read the information provided in this consent form, including the risks and possible benefits. All my questions about the research have been answered satisfactorily. I understand that my child is free to withdraw from the study at any time without repercussions or loss of benefits to which they are entitled.

I give my consent for my child/ward to participate in this study.

INFORMED CONSENT

If there is any part of this consent form that you do not understand, ask the investigator before you sign.

I, ____________________ (Name of father/mother/guardian) give my voluntary consent for my child or ward to participate in the study: “A prospective surveillance study to detect antimalarial drug resistance, gene deletions of diagnostic relevance and genetic diversity of *Plasmodium falciparum* in Mozambique.”

My questions have all been answered by ________________ (Name of researcher) in my own language. In case I have any other questions, I know that I can contact the study focal person assigned to Malaria Consortium and the National Bioethics for Health Committee through the contacts provided. I understand that I may withdraw my child’s participation from the study, at any time for any reason, without any repercussions.

Do you allow your child/ward’s samples to be stored and used in future research? □ **Yes** □ **No**

I agree for my child/ward to take part in this study.

Signatures

_____________________________ ____________

Signature of father/mother/guardian Date and time

Father/Mother/guardian fingerprint if they cannot sign

____

Name of father/mother/guardian (in capital letters)

____

Signature of the person who explained consent

_____________________________________ ______________

Name of the person who explained consent (in capital letters) Date and time

If the father/mother/guardian does not know how to read, an impartial witness must also sign this form:

____ ___________

Signature of the impartial witness Date and time

____

Name of the impartial witness (in capital letters)

For peer review only - http://bmjopen.bmj.com/site/about/guidelines.xhtml

Appendix 3. Information sheet and informed consent for adult pregnant women.

PART I

INFORMATION SHEET AND INFORMED CONSENT FOR ADULT PREGNANT WOMEN

Name of Affiliated Institutions

25. Manhiça Health Research Center (CISM), Manhiça, Mozambique
26. Malaria Consortium, Maputo, Mozambique
27. Barcelona Global Health Institute (ISGlobal), Barcelona, Spain
28. National Malaria Control Programme, Ministry of Health, Maputo, Mozambique
29. University of California, San Francisco, USA
30. Clinton Health Access Initiative, Boston, USA
31. Institute of Disease Modeling, Bill and Melinda Gates Foundation, Seattle, USA
32. Bill and Melinda Gates Foundation, Seattle, USA

Protocol title and version: "A prospective surveillance study to detect antimalarial drug resistance, gene deletions of diagnostic relevance and genetic diversity of *Plasmodium falciparum* in Mozambique," version number 7, 25 August 2021.

Name and affiliation of Principal Investigator(s): Baltazar Candrinho, National Malaria Control Programme, Ministry of Health, Maputo, Mozambique and Alfredo Mayor, Manhiça Health Research Center, Manhiça, Mozambique.

Study funder: Bill and Melinda Gates Foundation, USA

Introduction: The National Malaria Control Programme in partnership with Malaria Consortium and the Manhiça Health Research Center are conducting a study to analyse the genetics of malaria parasites to identify the best ways to control and/or eliminate this disease from the country.

Please read this form with care. This form provides important information about participating in this study. All the information which follows, discussed below, is to allow you to understand what the study involves and the steps that would need your collaboration, so that before becoming involved in the study, you can decide freely if you wish to participate.

You can take the time that you feel necessary to decide about your participation in this study. If you have questions about the study, or any part of this form, please ask us. If you decide to participate in this research, you will be asked to sign this form. One copy of the signed form will be provided to you for your records. If at any time you feel that you do not understand the information that is being provided, please do not hesitate to interrupt so that we can explain and clarify everything again.

After receiving your consent to participate, we will ask you some personal questions about your age, date of birth, recent illnesses, including history of fever, occupation, travel history, residence, use of insecticide treated mosquito nets or taking of antimalarial medications in the last month and then we will take a few drops of blood from your finger.

Rationale: Mozambique constitutes a main goal for the World Health Organization and partnership initiative, namely, Roll Back Malaria, to end malaria in the world. In this context, through involvement in regional malaria elimination initiatives, the use of molecular malaria surveillance data, as a complement to traditional surveillance information, can contribute to the elimination of malaria in Southern Mozambique and a reduction of the burden in the north of the country. However, there is a lack of malaria diagnostic and drug resistance data and other measures of the genetic diversity of the parasite that causes malaria in different transmission settings. Therefore, more evidence is needed to demonstrate the feasibility of using genetic data as a driver of the intensity of transmission in high transmission areas. Additionally, understanding the prevalence of

For peer review only - http://bmjopen.bmj.com/site/about/guidelines.xhtml

diagnostic and drug resistance and genetic diversity will inform more appropriate and impactful interventions to reduce malaria morbidity and mortality in Mozambique. The integration of genetic data into routine surveillance activities has the potential to increase knowledge for programmatic decision-making on the optimal combination of control and elimination measures in Mozambique.

Research objectives: Your participation in this study will help us to identify the prevalence of molecular markers of antimalarial resistance along with other genetic markers, which will inform the National Malaria Control Programme to support decision-making on the use of antimalarials and best strategies to control and eliminate malaria in the country.

Type of research/Intervention: This is prospective, operational surveillance research.

Selection of participants: You were invited to participate in this research because you are part of a group that is the focus of this study: **adult pregnant women** with malaria, confirmed by a rapid diagnostic test, living in this region.

Voluntary participation: It's your choice if you want to participate in this study or not. Refusing to participate or withdrawing your participation will not result in any penalty or loss of health benefits or services. You will continue to receive medical care if you choose not to participate in this study. Your decision will not change the care that you receive now or in the future. Participating in this study is your choice. If you decide to participate in this study, you can leave at any time without consequences. If you want to stop participating in the study, just let the research team know.

Procedures: We will take a few drops of blood from your finger and four drops will be placed on two small pieces of paper (filter paper), two drops on each paper. The filter papers containing four drops of blood each will be kept in the Health Unit and sent to Manhiça Health Research Center where the analysis will be done. If necessary, the filter papers may be sent to a laboratory located outside of Mozambique (specifically, the ISGlobal laboratory in Spain or the University of California, San Francisco laboratory, in the United States) for additional analysis and molecular characterisation of the malaria parasites (alleles related to antimalarial resistance as well as genetic composition and other molecular markers of relevance to malaria surveillance, both in the parasite and human host). The filter papers will be stored by the Manhiça Health Research Center for future human and parasite malaria molecular studies for a period of up to 10 years. In addition to drops of blood, all participants will also be asked about their age, date of birth, recent illnesses, including history of fever, occupation, travel history, residence, use of insecticide treated mosquito nets or antimalaria medication taking in the past 24/48 hours.

Risks, Discomfort and Inconvenience: You may feel a little pain or fear when your finger is pricked. The pain will dissipate within a few hours.

Benefits: There are no direct benefits for you to participate in this study. However, the findings generated from the study will inform the National Malaria Control Programme in decision-making about the use of antimalarials and the best strategies to control and eliminate malaria from the country.

Costs of Participation/Compensation: You will not receive any money or compensation to take part in this study.

Privacy: The data collected will be anonymous, however the data obtained in this study may be shared with collaborating partners: the National Malaria Control Programme, Malaria Consortium Mozambique, Manhiça Health Research Center, ISGlobal, Institute of Disease Modeling and the University of California, San Francisco, USA. In relation to the DNA sequences of the malaria parasite, or your personal data, these will be archived in an online database that can be shared with other scientists and researchers when the data are sent to scientific publications to report the results of this study.

Confidentiality: The information collected will be kept confidential and only the study team will have access to individuals' information. The results of the study will be published and made available so that other interested

For peer review only

people can learn from our study, but confidential information will not be shared in any circumstance. Your data will be completely anonymised.

Sharing of results: Results from this research will be shared on open access platforms online, in public data repositories or directly in scientific publications, in order to facilitate further collaboration, enhance trust in the findings and goodwill among researchers. We will specifically focus on data sharing among other African countries in the region which are engaging in similar approaches to the molecular surveillance of malaria.

Whom to Contact (Investigators and Ethics Committee): in case of any of these situations:

- If your questions, concerns or complaints are not being addressed by the research team;
- If you are unable to contact the research team;
- If you would like to speak with someone who is not part of the research team;
- If you have questions about your rights as a research participant;
- If you wish to obtain information or provide information about this research; or
- If you think that the study has caused harm.

Please return to the Health Unit and speak with the workers involved in the study or contact the study focal person, assigned by Malaria Consortium Mozambique, Neide Canana on telephone number: 860450563, or you can contact her at: Malaria Consortium Mozambique, Sita Av. Lucas Elias Kumato nr. 118, Bairro da Sommerschield – Maputo City, Mozambique, or you can also contact Manhiça Health Research Center, located at: Street 12, Bairro Cambeve in Município da Manhiça Maputo Province, Mozambique, or by telephone: 21810002. In case you are not satisfied with the responses provided, you may also contact the National Bioethics for Health Committee, Ministry of Health, Mozambique on the numbers: 824066350/844693186.

Ethics Committee approval of this study: This study was approved by the Manhiça Health Research Center Institutional Bioethics Health Committee and the National Bioethics for Health Committee.

PART II

DECLARATION OF INFORMED CONSENT

Study Title: "A prospective surveillance study to detect antimalarial drug resistance, gene deletions of diagnostic relevance and genetic diversity of *Plasmodium falciparum* in Mozambique."

Declaration: I have read the information provided in this consent form, including the risks and possible benefits. All my questions about the research have been answered satisfactorily. I understand that I am free to withdraw from the study at any time without repercussions or loss of benefits to which I am entitled.

I give my consent to participate in this study.

INFORMED CONSENT

If there is any part of this consent form that you do not understand, ask the investigator before you sign.

I, ______________________ (Name of participant) give my voluntary consent to participate in the study: "A prospective surveillance study to detect antimalarial drug resistance, gene deletions of diagnostic relevance and genetic diversity of *Plasmodium falciparum* in Mozambique."

My questions have all been answered by _________________ (Name of researcher) in my own language. In case I have any other questions, I know that I can contact the study focal person assigned to Malaria Consortium and the National Bioethics for Health Committee through the contacts provided. I understand that I may withdraw my participation from the study, at any time for any reason, without any repercussions.

For peer review only - http://bmjopen.bmj.com/site/about/guidelines.xhtml

Do you allow your samples to be stored and used in future research? □ **Yes** □ **No**

I agree to take part in this study.

Signatures

Participant's fingerprint if they cannot sign

Signature of participant   Date and time

Name of participant (in capital letters)

Signature of the person who explained consent

Name of the person who explained consent (in capital letters)   Date and time

If the participant does not know how to read, an impartial witness must also sign this form:

Signature of the impartial witness   Date and time

Name of the impartial witness (in capital letters)

For peer review only

Appendix 4. Information sheet and informed assent for pregnant women between 12 and 18 years of age.

PART I

INFORMATION SHEET AND INFORMED ASSENT FOR PREGNANT WOMEN BETWEEN 12 AND 18 YEARS OF AGE

Name of Affiliated Institutions

33. Manhiça Health Research Center (CISM), Manhiça, Mozambique
34. Malaria Consortium, Maputo, Mozambique
35. Barcelona Global Health Institute (ISGlobal), Barcelona, Spain
36. National Malaria Control Programme, Ministry of Health, Maputo, Mozambique
37. University of California, San Francisco, USA
38. Clinton Health Access Initiative, Boston, USA
39. Institute of Disease Modeling, Bill and Melinda Gates Foundation, Seattle, USA
40. Bill and Melinda Gates Foundation, Seattle, USA

Protocol title and version: "A prospective surveillance study to detect antimalarial drug resistance, gene deletions of diagnostic relevance and genetic diversity of *Plasmodium falciparum* in Mozambique," version number 7, 25 August 2021.

Name and affiliation of Principal Investigator(s): Baltazar Candrinho, National Malaria Control Programme, Ministry of Health, Maputo, Mozambique and Alfredo Mayor, Manhiça Health Research Center, Manhiça, Mozambique.

Study funder: Bill and Melinda Gates Foundation, USA

Introduction: The National Malaria Control Programme in partnership with Malaria Consortium and the Manhiça Health Research Center are conducting a study to analyse the genetics of malaria parasites to identify the best ways to control and/or eliminate this disease from the country.

Please read this form with care. This form provides important information about participating in this study. All the information which follows, discussed below, is to allow you to understand what the study involves and the steps that would need your collaboration, so that before becoming involved in the study, you can decide freely if you wish to participate.

You can take the time that you feel necessary to decide about your participation in this study. If you have questions about the study, or any part of this form, please ask us. If you decide to participate in this research, you will be asked to sign this form. One copy of the signed form will be provided to you for your records. If at any time you feel that you do not understand the information that is being provided, please do not hesitate to interrupt so that we can explain and clarify everything again.

After receiving your consent to participate, we will ask you some personal questions about your age, date of birth, recent illnesses, including history of fever, occupation, travel history, residence, use of insecticide treated mosquito nets or taking of antimalarial medications in the last month and then we will take a few drops of blood from your finger.

Rationale: Mozambique constitutes a main goal for the World Health Organization and partnership initiative, namely, Roll Back Malaria, to end malaria in the world. In this context, through involvement in regional malaria elimination initiatives, the use of molecular malaria surveillance data, as a complement to traditional surveillance information, can contribute to the elimination of malaria in Southern Mozambique and a reduction of the burden in the north of the country. However, there is a lack of malaria diagnostic and drug resistance data and other measures of the genetic diversity of the parasite that causes malaria in different transmission settings. Therefore, more evidence is needed to demonstrate the feasibility of using genetic data as a driver of the intensity of transmission in high transmission areas. Additionally, understanding the prevalence of

For peer review only - http://bmjopen.bmj.com/site/about/guidelines.xhtml

diagnostic and drug resistance and genetic diversity will inform more appropriate and impactful interventions to reduce malaria morbidity and mortality in Mozambique. The integration of genetic data into routine surveillance activities has the potential to increase knowledge for programmatic decision-making on the optimal combination of control and elimination measures in Mozambique.

Research objectives: Your participation in this study will help us to identify the prevalence of molecular markers of antimalarial resistance along with other genetic markers, which will inform the National Malaria Control Programme to support decision-making on the use of antimalarials and best strategies to control and eliminate malaria in the country.

Type of research/Intervention: This is prospective, operational surveillance research.

Selection of participants: You were invited to participate in this research because you are part of a group that is the focus of this study: **pregnant women between 12 to 18 years of age** with malaria, confirmed by a rapid diagnostic test, living in this region.

Voluntary participation: It's your choice if you want to participate in this study or not. Refusing to participate or withdrawing your participation will not result in any penalty or loss of health benefits or services. You will continue to receive medical care if you choose not to participate in this study. Your decision will not change the care that you receive now or in the future. Participating in this study is your choice. If you decide to participate in this study, you can leave at any time without consequences. If you want to stop participating in the study, just let the research team know.

Procedures: We will take a few drops of blood from your finger and four drops will be placed on two small pieces of paper (filter paper), two drops on each paper. The filter papers containing four drops of blood each will be kept in the Health Unit and sent to Manhiça Health Research Center where the analysis will be done. If necessary, the filter papers may be sent to a laboratory located outside of Mozambique (specifically, the ISGlobal laboratory in Spain or the University of California, San Francisco laboratory, in the United States) for additional analysis and molecular characterisation of the malaria parasites (alleles related to antimalarial resistance as well as genetic composition and other molecular markers of relevance to malaria surveillance, both in the parasite and human host). The filter papers will be stored by the Manhiça Health Research Center for future human and parasite malaria molecular studies for a period of up to 10 years. In addition to drops of blood, all participants will also be asked about their age, date of birth, recent illnesses, including history of fever, occupation, travel history, residence, use of insecticide treated mosquito nets or antimalaria medication taking in the past 24/48 hours.

Risks, Discomfort and Inconvenience: You may feel a little pain or fear when your finger is pricked. The pain will dissipate within a few hours.

Benefits: There are no direct benefits for you to participate in this study. However, the findings generated from the study will inform the National Malaria Control Programme in decision-making about the use of antimalarials and the best strategies to control and eliminate malaria from the country.

Costs of Participation/Compensation: You will not receive any money or compensation to take part in this study.

Privacy: The data collected will be anonymous, however the data obtained in this study may be shared with collaborating partners: the National Malaria Control Programme, Malaria Consortium Mozambique, Manhiça Health Research Center, ISGlobal, Institute of Disease Modeling and the University of California, San Francisco, USA. In relation to the DNA sequences of the malaria parasite, or your personal data, these will be archived in an online database that can be shared with other scientists and researchers when the data are sent to scientific publications to report the results of this study.

Confidentiality: The information collected will be kept confidential and only the study team will have access to individuals' information. The results of the study will be published and made available so that other interested

For peer review only - http://bmjopen.bmj.com/site/about/guidelines.xhtml

people can learn from our study, but confidential information will not be shared in any circumstance. Your data will be completely anonymised.

Sharing of results: Results from this research will be shared on open access platforms online, in public data repositories or directly in scientific publications, in order to facilitate further collaboration, enhance trust in the findings and goodwill among researchers. We will specifically focus on data sharing among other African countries in the region which are engaging in similar approaches to the molecular surveillance of malaria.

Whom to Contact (Investigators and Ethics Committee): in case of any of these situations:

- If your questions, concerns or complaints are not being addressed by the research team;
- If you are unable to contact the research team;
- If you would like to speak with someone who is not part of the research team;
- If you have questions about your rights as a research participant;
- If you wish to obtain information or provide information about this research; or
- If you think that the study has caused harm.

Please return to the Health Unit and speak with the workers involved in the study or contact the study focal person, assigned by Malaria Consortium Mozambique, Neide Canana on telephone number: 860450563, or you can contact her at: Malaria Consortium Mozambique, Sita Av. Lucas Elias Kumato nr. 118, Bairro da Sommerschield – Maputo City, Mozambique, or you can also contact Manhiça Health Research Center, located at: Street 12, Bairro Cambeve in Município da Manhiça Maputo Province, Mozambique, or by telephone: 21810002. In case you are not satisfied with the responses provided, you may also contact the National Bioethics for Health Committee, Ministry of Health, Mozambique on the numbers: 824066350/844693186.

Ethics Committee approval of this study: This study was approved by the Manhiça Health Research Center Institutional Bioethics Health Committee and the National Bioethics for Health Committee.

PART II

DECLARATION OF ASSENT

Study Title: "A prospective surveillance study to detect antimalarial drug resistance, gene deletions of diagnostic relevance and genetic diversity of *Plasmodium falciparum* in Mozambique."

Declaration: I have read the information provided in this assent form, including the risks and possible benefits. All my questions about the research have been answered satisfactorily. I understand that I am free to withdraw from the study at any time without repercussions or loss of benefits to which I am entitled.

I give my assent to participate in this study.

INFORMED ASSENT

If there is any part of this assent form that you do not understand, ask the investigator before you sign.

I, ______________________ (Name of participant) give my voluntary assent to participate in the study: "A prospective surveillance study to detect antimalarial drug resistance, gene deletions of diagnostic relevance and genetic diversity of *Plasmodium falciparum* in Mozambique."

My questions have all been answered by __________________ (Name of researcher) in my own language. In case I have any other questions, I know that I can contact the study focal person assigned to Malaria Consortium and the National Bioethics for Health Committee through the contacts provided. I understand that I may withdraw my participation from the study, at any time for any reason, without any repercussions.

For peer review only - http://bmjopen.bmj.com/site/about/guidelines.xhtml

Do you allow your samples to be stored and used in future research? □ **Yes** □ **No**

I agree to take part in this study.

Signatures

Minor's fingerprint if they cannot sign

______________________ ____________
Signature of the minor Date and time

____
Minor's name (in capital letters)

____
Signature of the person who explained assent

______________________ ____________
Name of the person who explained assent (in capital letters) Date and time

If the minor does not know how to read, an impartial witness must also sign this form:

______________________ ____________
Signature of the impartial witness Date and time

____
Name of the impartial witness (in capital letters)

PART III

INFORMATION SHEET AND INFORMED CONSENT FOR PARENTS/GUARDIANS OF PREGNANT WOMEN LESS THAN 18 YEARS OF AGE

Name of Affiliated Institutions

41. Manhiça Health Research Center (CISM), Manhiça, Mozambique
42. Malaria Consortium, Maputo, Mozambique
43. Barcelona Global Health Institute (ISGlobal), Barcelona, Spain
44. National Malaria Control Programme, Ministry of Health, Maputo, Mozambique
45. University of California, San Francisco, USA
46. Clinton Health Access Initiative, Boston, USA
47. Institute of Disease Modeling, Bill and Melinda Gates Foundation, Seattle, USA
48. Bill and Melinda Gates Foundation, Seattle, USA

Protocol title and version: "A prospective surveillance study to detect antimalarial drug resistance, gene deletions of diagnostic relevance and genetic diversity of *Plasmodium falciparum* in Mozambique," version number 7, 25 August 2021.

Name and affiliation of Principal Investigator(s): Baltazar Candrinho, National Malaria Control Programme, Ministry of Health, Maputo, Mozambique and Alfredo Mayor, Manhiça Health Research Center, Manhiça, Mozambique.

Study funder: Bill and Melinda Gates Foundation, USA

Introduction: The National Malaria Control Programme in partnership with Malaria Consortium and the Manhiça Health Research Center are conducting a study to analyse the genetics of malaria parasites to identify the best ways to control and/or eliminate this disease from the country.

For peer review only - http://bmjopen.bmj.com/site/about/guidelines.xhtml

Please read this form with care. This form provides important information about participating in this study. All the information which follows, discussed below, is to allow you to understand what the study involves and the steps that would need your collaboration, so that before becoming involved in the study, you can decide freely if you wish to participate.

You can take the time that you feel necessary to decide about your participation in this study. If you have questions about the study, or any part of this form, please ask us. If you decide for your child/ward to participate in this research, you will be asked to sign this form. One copy of the signed form will be provided to you for your records. If at any time you feel that you do not understand the information that is being provided, please do not hesitate to interrupt so that we can explain and clarify everything again.

After receiving your consent for your child/ward to participate, we will ask them some personal questions about their age, date of birth, recent illnesses, including history of fever, occupation, travel history, residence, use of insecticide treated mosquito nets or taking of antimalarial medications in the last month and then we will take a few drops of blood from their finger.

Rationale: Mozambique constitutes a main goal for the World Health Organization and partnership initiative, namely, Roll Back Malaria, to end malaria in the world. In this context, through involvement in regional malaria elimination initiatives, the use of molecular malaria surveillance data, as a complement to traditional surveillance information, can contribute to the elimination of malaria in Southern Mozambique and a reduction of the burden in the north of the country. However, there is a lack of malaria diagnostic and drug resistance data and other measures of the genetic diversity of the parasite that causes malaria in different transmission settings. Therefore, more evidence is needed to demonstrate the feasibility of using genetic data as a driver of the intensity of transmission in high transmission areas. Additionally, understanding the prevalence of diagnostic and drug resistance and genetic diversity will inform more appropriate and impactful interventions to reduce malaria morbidity and mortality in Mozambique. The integration of genetic data into routine surveillance activities has the potential to increase knowledge for programmatic decision-making on the optimal combination of control and elimination measures in Mozambique.

Research objectives: Your child/ward's participation in this study will help us to identify the prevalence of molecular markers of antimalarial resistance along with other genetic markers, which will inform the National Malaria Control Programme to support decision-making on the use of antimalarials and best strategies to control and eliminate malaria in the country.

Type of research/Intervention: This is prospective, operational surveillance research.

Selection of participants: Your child/ward was invited to participate in this research because they are part of a group that is the focus of this study: **pregnant women between 12 to 18 years of age** with malaria, confirmed by a rapid diagnostic test, living in this region.

Voluntary participation: It's your choice if you want your child/ward to participate in this study or not. Refusing to participate or withdrawing their participation will not result in any penalty or loss of health benefits or services. Your child/ward will continue to receive medical care if you/they choose not to participate in this study. Your decision will not change the care that they receive now or in the future. Participating in this study is your/their choice. If you decide for them to participate in this study, they can leave at any time without consequences. If they want to stop participating in the study, just let the research team know.

Procedures: We will take a few drops of blood from your child/ward's finger and four drops will be placed on two small pieces of paper (filter paper), two drops on each paper. The filter papers containing four drops of blood each will be kept in the Health Unit and sent to Manhiça Health Research Center where the analysis will be done. If necessary, the filter papers may be sent to a laboratory located outside of Mozambique (specifically, the ISGlobal laboratory in Spain or the University of California, San Francisco laboratory, in the United States) for additional analysis and molecular characterisation of the malaria parasites (alleles related to antimalarial resistance as well as genetic composition and other molecular markers of relevance to malaria surveillance,

For peer review only - http://bmjopen.bmj.com/site/about/guidelines.xhtml

both in the parasite and human host). The filter papers will be stored by the Manhiça Health Research Center for future human and parasite malaria molecular studies for a period of up to 10 years. In addition to drops of blood, all participants will also be asked about their age, date of birth, recent illnesses, including history of fever, occupation, travel history, residence, use of insecticide treated mosquito nets or antimalaria medication taking in the past 24/48 hours.

Risks, Discomfort and Inconvenience: Your child/ward may feel a little pain or fear when their finger is pricked. The pain will dissipate within a few hours.

Benefits: There are no direct benefits for you to participate in this study. However, the findings generated from the study will inform the National Malaria Control Programme in decision-making about the use of antimalarials and the best strategies to control and eliminate malaria from the country.

Costs of Participation/Compensation: You will not receive any money or compensation for your child/ward to take part in this study.

Privacy: The data collected will be anonymous, however the data obtained in this study may be shared with collaborating partners: the National Malaria Control Programme, Malaria Consortium Mozambique, Manhiça Health Research Center, ISGlobal, Institute of Disease Modeling and the University of California, San Francisco, USA. In relation to the DNA sequences of the malaria parasite, or your child/ward's personal data, these will be archived in an online database that can be shared with other scientists and researchers when the data are sent to scientific publications to report the results of this study.

Confidentiality: The information collected will be kept confidential and only the study team will have access to individuals' information. The results of the study will be published and made available so that other interested people can learn from our study, but confidential information will not be shared in any circumstance. Your child/ward's data will be completely anonymised.

Sharing of results: Results from this research will be shared on open access platforms online, in public data repositories or directly in scientific publications, in order to facilitate further collaboration, enhance trust in the findings and goodwill among researchers. We will specifically focus on data sharing among other African countries in the region which are engaging in similar approaches to the molecular surveillance of malaria.

Whom to Contact (Investigators and Ethics Committee): in case of any of these situations:

- If your questions, concerns or complaints are not being addressed by the research team;
- If you are unable to contact the research team;
- If you would like to speak with someone who is not part of the research team;
- If you have questions about your rights as a research participant;
- If you wish to obtain information or provide information about this research; or
- If you think that the study has caused harm.

Please return to the Health Unit and speak with the workers involved in the study or contact the study focal person, assigned by Malaria Consortium Mozambique, Neide Canana on telephone number: 860450563, or you can contact her at: Malaria Consortium Mozambique, Sita Av. Lucas Elias Kumato nr. 118, Bairro da Sommerschield – Maputo City, Mozambique, or you can also contact Manhiça Health Research Center, located at: Street 12, Bairro Cambeve in Município da Manhiça Maputo Province, Mozambique, or by telephone: 21810002. In case you are not satisfied with the responses provided, you may also contact the National Bioethics for Health Committee, Ministry of Health, Mozambique on the numbers: 824066350/844693186.

Ethics Committee approval of this study: This study was approved by the Manhiça Health Research Center Institutional Bioethics Health Committee and the National Bioethics for Health Committee.

PART IV

For peer review only - http://bmjopen.bmj.com/site/about/guidelines.xhtml

DECLARATION OF INFORMED CONSENT

Study Title: "A prospective surveillance study to detect antimalarial drug resistance, gene deletions of diagnostic relevance and genetic diversity of *Plasmodium falciparum* in Mozambique."

Declaration: I have read the information provided in this consent form, including the risks and possible benefits. All my questions about the research have been answered satisfactorily. I understand that my child/ward is free to withdraw from the study at any time without repercussions or loss of benefits to which I am entitled.

I give my consent for my child/ward to participate in this study.

INFORMED CONSENT

If there is any part of this consent form that you do not understand, ask the investigator before you sign.

I, ______________________ (Name of father/mother/guardian) give my voluntary consent for my child or ward to participate in the study: "A prospective surveillance study to detect antimalarial drug resistance, gene deletions of diagnostic relevance and genetic diversity of *Plasmodium falciparum* in Mozambique."

My questions have all been answered by _________________ (Name of researcher) in my own language. In case I have any other questions, I know that I can contact the study focal person assigned to Malaria Consortium and the National Bioethics for Health Committee through the contacts provided. I understand that my child/ward may withdraw their participation from the study, at any time for any reason, without any repercussions.

Do you allow your child/ward's samples to be stored and used in future research? □ **Yes** □ **No**

I agree for my child/ward to take part in this study.

Signatures

________________________________ ______________

Signature of father/mother/guardian Date and time

Father/mother/guardian fingerprint if they cannot sign

____

Name of father/mother/guardian (in capital letters)

____

Signature of the person who explained consent

____ ________________

Name of the person who explained consent (in capital letters) Date and time

If the participant/legal representative does not know how to read, an impartial witness must also sign this form:

____ _____________

Signature of the impartial witness Date and time

____

Name of the impartial witness (in capital letters)

For peer review only

Appendix 5. Questionnaire for medium-high transmission area, children under 2-10 years old.

	**Study site information**	
1.	Date of sample collection (dd/mm/yy)	|__|__||__|__||__|__|
2.	Province of residence	[ ]
3.	District of residence	[ ]
4.	Administrative post	[ ]
5.	Place of residence	[ ]
6.	Health Unit (name or code)	[ ]
7.	Referred by APE in the community	Yes ☐ No ☐
	**Inclusion criteria**	
8.	Was the informed consent form signed?<br>***If no, end the survey.***	Yes ☐ No ☐
9.	History of fever/hot body in the last 24 hours?	Yes ☐ No ☐
10.	Axillary temperature at the time of the survey<br>***If temperature is <37.5ºC, end the survey.***	|__|__|.|__| ºC
11.	Date of birth (dd/mm/yyyy)<br><br>11.1. Age (years)	|__|__|/|__|__|/|__|__|__|__| |__|__|
12.	Does the participant have severe malaria?<br>**If yes, end the survey.**	Yes ☐ No ☐
13.	Does the participant reside in the study area (district)?<br>***If no, end the survey.***	Yes ☐ No ☐
14.	Has the child taken antimalarials in the past 14 days? (check yellow health card)<br>***If yes, end the survey.***	Yes ☐ No ☐
15.	Was a routine RDT performed?<br>***If no, end the survey.***	Yes ☐ No ☐
15.1	*If yes, the result was:*<br><br>***If negative, end the survey.***	Positive ☐ Negative ☐ Inconclusive ☐
16.	Was an additional RDT performed?	Yes ☐ No ☐ Not applicable ☐<br>**If 'not applicable', skip to question 17.**
	16.1. Result of line T1 (HRP2)	Positive ☐ Negative ☐ Inconclusive ☐
	16.2. Result of line T2 (LDH)	Positive ☐ Negative ☐ Inconclusive ☐
	**Participant information**	
17.	Sex	Male ☐ Female ☐
18.	Study ID number<br><br>*US*|__|__|-|__|__|-|__|__|__|	19. Sample ID number — *Insert bar code*<br><br>**Now put the sample ID number on the informed consent form.**
	**Travel information**	
20.	Have you travelled in the past 28 days?	Yes ☐ No ☐

For peer review only

If not, go to question 21.

20.1 When did you start your trip? (date: dd/mm)	|__|__||__|__|
20.2 If yes, for how many nights?	
20.3 Where did you travel?:	|__|__|
Country	[____________]
Province	[____________]
District	[____________]
20.4 During the trip, did you sleep under a mosquito net?	Yes ☐ No ☐

Information related to malaria

21. How many times has the child had episodes of fever in the past month?	|__|__|
22. Did the child sleep under a mosquito net last night??	Yes ☐ No ☐
22.1 *If yes, was it an insecticide treated net?*	Yes ☐ No ☐
23. Has there been indoor residual spraying in the past 6 months?	Yes ☐ No ☐
24. Has the child taken antimalarial medications in the past month?	Yes ☐ No ☐
25. Is the child taking cotrimoxazole?	Yes ☐ No ☐ Don`t know ☐

Now label the two filter papers (sample ID number)

26. Was a blood sample collected on the filter paper?	Yes ☐ No ☐
27. If yes, state the number of papers	2 ☐ Other |__|

Interviewer information

28. Interviewer number	|__|__|__|__|__|
29. Interviewer initials	|__|__|__|__|__|
30. Date of interview	(dd/mm/yyyy) |__|__|/|__|__|/|__|__|__|__|

For peer review only

Appendix 6. Questionnaire for low transmission area, all ages.

	**Study site information**	
1.	Date of sample collection (dd/mm/yy)	|__|__||__|__||__|__|
2.	Province of residence	[ ]
3.	District of residence	[ ]
4.	Administrative post	[ ]
5.	Place of residence	[ ]
6.	Health Unit (name or code)	[ ]
7.	Referred by APE in the community	Yes ☐ No ☐
	**Inclusion criteria**	
8.	Was the informed consent form signed? ***If no, end the survey.***	Yes ☐ No ☐
9.	History of fever/hot body in the last 24 hours?	Yes ☐ No ☐
10.	Axillary temperature at the time of the survey ***If temperature is <37.5ºC, end the survey.***	|__|__|.|__| ºC
11.	Date of birth (dd/mm/yyyy)<br>11.1. Age (years)	|__|__|/|__|__|/|__|__|__|__| |__|__|
12.	Does the participant have severe malaria? ***If yes, end the survey.***	Yes ☐ No ☐
13.	Does the participant reside in the study area (district)? ***If no, end the survey.***	Yes ☐ No ☐
14.	Has the child/adult taken antimalarials in the past 14 days? (check yellow health card) ***If yes, end the survey.***	Yes ☐ No ☐
15.	Was a routine RDT performed? ***If no, end the survey.***<br>15.1 *If yes, the result was:*<br>***If negative, end the survey.***	Yes ☐ No ☐<br>Positive ☐ Negative ☐ Inconclusive ☐
16.	Was an additional RDT performed?	Yes ☐ No ☐ Not applicable ☐ **If 'not applicable', skip to question 17.**
	16.1. Result of line T1 (HRP2)	Positive ☐ Negative ☐ Inconclusive ☐
	16.2. Result of line T2 (LDH)	Positive ☐ Negative ☐ Inconclusive ☐
	**Participant information**	
17.	Sex	Male ☐ Female ☐
18.	Occupation	[ ]
19.	Study ID number<br>US|__|__|-|__|__|-|__|__|__|	**Sample ID number** *Insert bar code*<br>**Now put the sample ID number on the informed consent form.**

For peer review only - http://bmjopen.bmj.com/site/about/guidelines.xhtml

Travel information		
20.	Have you travelled in the past 28 days?<br>***If not, go to question 21.***	Yes ☐ No ☐
	20.1 When did you start your trip? (date: dd/mm)	|__|__||__|__|
	20.2 If yes, for how many nights?	|__|__|
	20.3 Where did you travel?:<br>Country<br>Province<br>District	[____________]<br>[____________]<br>[____________]
	20.4 During the trip, did you sleep under a mosquito net?	Yes ☐ No ☐
**Information related to malaria**		
21.	How many times has the child/adult had episodes of fever in the past month?	|__|__|
22.	Did the child/adult sleep under a mosquito net last night?	Yes ☐ No ☐
	22.1 *If yes, was it an insecticide treated net?*	Yes ☐ No ☐
23.	Has there been indoor residual spraying in the past 6 months?	Yes ☐ No ☐
24.	Has the child/adult taken antimalarial medications in the past month?	Yes ☐ No ☐
25.	Is the child/adult taking cotrimoxazole?	Yes ☐ No ☐ Don`t know ☐
	Now label the two filter papers (sample ID number)	
26.	Was a blood sample collected on the filter paper?	Yes ☐ No ☐
27.	If yes, state the number of papers	2 ☐ Other |__|
**Interviewer information**		
28.	Interviewer number	|__|__|__|__|
29.	Interviewer initials	|__|__|__|__|
30.	Date of interview	(dd/mm/yyyy) |__|__|/|__|__|/|__|__|__|__|

For peer review only

Appendix 7. Questionnaire for Pregnant women attending ANC clinic in medium-high transmission area.

	**Study site information**	
1.	Date of sample collection (dd/mm/yy)	|__|__||__|__||__|__|
2.	Province of residence	[________________]
3.	District of residence	[________________]
4.	Administrative post	[________________]
5.	Place of residence	[________________]
6.	Health Unit (name or code)	[________________]
7.	Referred by APE in the community	Yes ☐ No ☐
	**Inclusion criteria**	
8.	Was the informed consent form signed?<br>***If no, end the survey.***	Yes ☐ No ☐
9.	Is the participant pregnant?<br>***If no, end the survey.***	Yes ☐ No ☐
10.	Is this your first prenatal consult?<br>***If no, end the survey.***	Yes ☐ No ☐
11.	Date of birth (dd/mm/yyyy)	|__|__|/|__|__|/|__|__|__|__|
	11.1. Age (years)<br>***If aged <12 years, end the survey.***	|__|__|
12.	Does the participant reside in the study area (district)?<br>***If no, end the survey.***	Yes ☐ No ☐
13.	Does the participant have severe malaria?<br>***If yes, end the survey.***	Yes ☐ No ☐
	**Participant information**	
14.	Occupation	[________________]
15.	Study ID number<br>**PN**|__|__|-|__|__|-|__|__|__|__|	Sample ID number: *Insert bar code*

Now put the sample ID number on the informed consent form.

	**Participant characteristics**	
16.	History of fever/hot body in the last 24 hours?	Yes ☐ No ☐
17.	Axillary temperature at the time of the survey	|__|__|.|__| ºC
18.	Was an HIV test performed during this visit? *(check HIV card or proof of testing)*	Yes ☐ No ☐
	18.1. *If yes, HIV test result at this visit*	Positive ☐ Negative ☐ Inconclusive ☐
	18.2. *If no, state previous HIV test result (check HIV card or proof of testing)*	Negative ☐ Positive ☐
19.	Are you receiving ART? *(check in the woman's personal health record)*	Yes ☐ No ☐
20.	Are you taking cotrimoxazole? *(check in the woman's book)*	Yes ☐ No ☐
21.	Current haemoglobin result *(Hemocue test result from today)*	|__|__|, |__| g/dL
22.	Was a malaria RDT performed?	Yes ☐ No ☐

For peer review only - http://bmjopen.bmj.com/site/about/guidelines.xhtml

23.	*If yes, the result:*	Positive ☐ Negative ☐ Inconclusive ☐
24.	How many weeks pregnant are you currently?	|__|__|
25.	Method used to determine gestational age:<br><br>a) Last menstrual period ☐<br>b) Fundal height ☐<br>c) Other (specify) [________________]	
26.	How many previous pregnancies has the participant had before this one?	|__|__|
27.	Has the participant moved from the area during the last pregnancy?	Yes ☐ No ☐
	**Travel information**	
28.	Have you travelled during this pregnancy and spent the night away from home?	Yes ☐ No ☐
	28.1 When did you start your trip? (date: dd/mm)	|__|__||__|__|
	28.2 If yes, for how many nights?	
	28.3 Where did you travel?:	|__|__|
	Country	[________________]
	Province	[________________]
	District	[________________]
	28.4 During the trip, did you sleep under a mosquito net?	Yes ☐ No ☐
	**Information related to malaria**	
29.	How many times have you had episodes of fever in the past month?	|__|__|
30.	Did you sleep under a mosquito net last night?	Yes ☐ No ☐
30.1	*If yes, was it an insecticide treated net?*	Yes ☐ No ☐
31.	Has there been indoor residual spraying in the past 6 months?	Yes ☐ No ☐
32.	Has the participant received intermittent preventive treatment (IPT) before this visit for this pregnancy?	Yes ☐ No ☐
33.	Has the participant taken antimalarial medications in the past month?	Yes ☐ No ☐ Don`t know ☐
	**Now label the filter paper with the sample ID number**	
34.	Was a blood sample collected on the filter paper?	Yes ☐ No ☐
35.	If yes, state the number of papers	2 ☐ Other |__|

	**Interviewer information**	
36.	Interviewer number	|__|__|__|__|__|
37.	Interviewer initials	|__|__|__|__|__|
38.	Date of interview	(dd/mm/yyyy) |__|__|/|__|__|/|__|__|__|__|

For peer review only - http://bmjopen.bmj.com/site/about/guidelines.xhtml

Appendix 8. Questionnaire for Pregnant women attending ANC clinic in low transmission area.

	**Study site information**	
1	Date of sample collection (dd/mm/yy)	|__|__||__|__||__|__|
2.	Province of residence	[ ]
3.	District of residence	[ ]
4.	Administrative post	[ ]
5.	Place of residence	[ ]
6.	Neighbourhood of residence	[ ]
7.	Mobile phone number	[ ]
8.	Health Unit (name or code)	[ ]
9.	Referred by APE in the community	Yes ☐ No ☐
	**Inclusion criteria**	
10.	Was the informed consent form signed? ***If no, end the survey.***	Yes ☐ No ☐
11.	Is the participant pregnant? ***If no, end the survey.***	Yes ☐ No ☐
12.	Is this your first prenatal consult? ***If no, end the survey.***	Yes ☐ No ☐
13.	Date of birth (dd/mm/yyyy) I__|__I/|__|__I/|__|__|__|__| 11.1. Age (years) |__|__| ***If aged <12 years, end the survey.***	
14.	Does the participant reside in the study area (district)? ***If no, end the survey.***	Yes ☐ No ☐
15.	Does the participant have severe malaria? ***If yes, end the survey.***	Yes ☐ No ☐
	**Participant information**	
16.	Participant name	[ ]
17.	Occupation	[ ]
18.	Study number<br>**PN**|__|__|-|__|__|-|__|__|__|	Sample ID number: *Insert bar code*

Now put the sample ID number on the informed consent form.

	**Participant characteristics**	
19.	History of fever/hot body in the last 24 hours?	Yes ☐ No ☐
20.	Axillary temperature at the time of the survey	|__|__|.|__| ºC
21.	Was an HIV test performed during this visit? (*check HIV card or proof of testing)*	Yes ☐ No ☐
	18.1. *If yes, HIV test result at this visit*	Positive ☐ Negative ☐ Inconclusive ☐
	18.2. *If no, state previous HIV test result (check HIV card or proof of testing)*	Negative ☐ Positive ☐

For peer review only - http://bmjopen.bmj.com/site/about/guidelines.xhtml

22.	Are you receiving ART? *(check in the woman's personal health record)*	Yes ☐ No ☐
23.	Are you taking cotrimoxazole? *(check in the woman's book)*	Yes ☐ No ☐
24.	Current haemoglobin result *(Hemocue test result from today)*	|__|__|, |__| g/dL
25.	Was a malaria RDT performed?	Yes ☐ No ☐
26.	*If yes, the result:*	Positive ☐ Negative ☐ Inconclusive ☐
27.	How many weeks pregnant are you currently?	|__|__|
28.	Method used to determine gestational age:<br>a) Last menstrual period ☐<br>b) Fundal height ☐<br>c) Other (specify) [____________]	
29.	How many previous pregnancies has the participant had before this one?	|__|__|
30.	Has the participant moved from the area during the last pregnancy?	Yes ☐ No ☐
	**Travel information**	
31.	Have you travelled during this pregnancy and spent the night away from home?	Yes ☐ No ☐
	31.1 When did you start your trip? (date: dd/mm)	|__|__||__|__|
	31.2 If yes, for how many nights?	
	31.3 Where did you travel?:	|__|__|
	Country	[____________]
	Province	[____________]
	District	[____________]
	28.4 During the trip, did you sleep under a mosquito net?	Yes ☐ No ☐
	**Information related to malaria**	
32.	How many times have you had episodes of fever in the past month?	|__|__|
33.	Did you sleep under a mosquito net last night?	Yes ☐ No ☐
33.1	*If yes, was it an insecticide treated net?*	Yes ☐ No ☐
34.	Has there been indoor residual spraying in the past 6 months?	Yes ☐ No ☐
35.	Has the participant received intermittent preventive treatment (IPT) before this visit for this pregnancy?	Yes ☐ No ☐
36.	Has the participant taken antimalarial medications in the past month?	Yes ☐ No ☐ Don\`t know ☐
	**Now label the filter paper with the sample ID number**	
37.	Was a blood sample collected on the filter paper?	Yes ☐ No ☐
38.	If yes, state the number of papers	2 ☐ Other |__|
	**Interviewer information**	
39.	Interviewer number	|__|__|__|__|__|
40.	Interviewer initials	|__|__|__|__|__|
41.	Date of interview	(dd/mm/yyyy) |__|__|/|__|__|/|__|__|__|__|

For peer review only - http://bmjopen.bmj.com/site/about/guidelines.xhtml

Appendix 9. Procedures for the collection, handling and storage of dried blood samples on filter paper and rapid diagnostic tests.

1 OBJECTIVES

To describe the correct collection, handling and storage procedures for dried blood samples on filter paper and rapid diagnostic tests (RDTs).

2 DEFINITIONS

- Filter paper: semipermeable paper used as a laboratory tool to collect and store blood samples for further molecular analysis. The filter paper code that will be used is Whatman Grade CF12 cut to 76x30mm (equal to the size of a microscope slide).
- **Rapid diagnostic test (RDT):** Lateral flow immunochromatographic tests. The RDTs for human malaria detect parasite specific antigens which are present in the blood of infected people. The most commonly used antigens are *Plasmodium falciparum Histidine-rich Protein 2* (PfHRP-2) and *Plasmodium Lactate Dehydrogenase* (pLDH).

3 APPLICABLE FOR

- All personnel responsible for the collection, handling and storage of dried blood samples on filter paper rand RDTs within the scope of malaria studies at CISM.

4 RESPONSIBILITIES

- **Investigators:** to guarantee that the SOPs are up to date and that technical personnel are properly trained and strictly follow the procedures described therein.
- **All technical personnel:** whether researcher, laboratory technicians, phlebotomists, physicians, or others who are engaged in field, clinical or laboratory activities involving filter papers or RDTs; all must know and strictly follow the content of these SOPs.

5 RELATED SOPS

- **POP_LB_012_PT**: Procedures for performing the malaria rapid diagnostic test (RDT)

6 SUPPLIES AND EQUIPMENT

Table 1. Requisite supplies

Type	Item
**Documents**	• Laboratory requisition form<br>• Health Unit sample record form<br>• Laboratory RDT sample placement form<br>• Laboratory filter paper placement form<br>• (Electronic document) Sample control in the laboratory
**Items for collection, transport and storage**	• Whatman Grade CF12 (ref. WHA10538018, slides 580x580mm) cut to slides sized 76x30mm<br>• Ziplock bags for individual samples (minimum length 80mm to the zip)<br>• Large Ziplock bags<br>• Lancets<br>• Silica gel<br>• Cotton balls<br>• Band aids<br>• Incinerator box<br>• Disposable gowns<br>• Freezer<br>• Refrigerator

For peer review only - http://bmjopen.bmj.com/site/about/guidelines.xhtml

	• Gloves (HI-CARE 2023-05)<br>• 8 sample identification numbers<br>• Alcohol (70%)<br>• Masks<br>• Sample transport cases<br>• Box for ground transport of samples
**Office supplies**	• Staples and staplers<br>• Markers (sharpie)<br>• Pens / Pencils<br>• 1 Printer<br>• Computer<br>• Toner<br>• Notebook<br>• Clipboard

7 PROCEDURE

7.1 Sample collection

7.1.1 Collection of blood sample on filter paper

- For each participant, four (4) circular capillary blood spots of approximately 50µl (equivalent to a diameter of 1.5-2cm) will be put on two Whatman CF12 filter papers, with two spots on each paper (**Figure 1**) in accordance with the following steps:
  1) Prepare 2 papers (76x30mm), alcohol (70%), cotton balls, sterile lancet.
  2) Clean the finger with a cotton ball soaked in 70% alcohol and wait for it to dry; it is recommended to use the middle or ring finger.
  3) Remove the protector part to release the sterile lancet.
  4) Firmly prick the side of the fingertip.
  5) Carefully squeeze and wipe the first drop of blood with a dry cotton ball.
  6) Let one or two drops of blood drip onto the filter paper for each blood spot in a diameter of approximately 1.5-2cm (**Figure 1**). It is important not to let the finger touch the filter paper, to avoid contamination; only a drop of blood may touch the paper.
  7) Wipe the fingertip with another cotton ball soaked in alcohol.

> NOTE: In case you collect a sample from a baby, and it is not possible to obtain two drops of blood from one of their fingers, alternatively, you can prick their heel. In this case, only one drop of blood on the filter paper is needed.

Figure 1. Placement of blood spots on the filter paper.

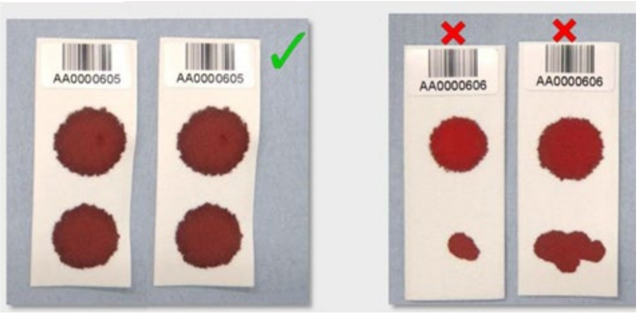

- Identify each sample with a sample identification number (the same sample identification number for each of the two filter papers) and include the collection date of the sample and the study acronym, using a pen.

For peer review only

- Put the same sample identification number on the **Laboratory analysis requisition form** (current version of POP_MAL_001_A01_PT) and fill out the form with the patient's details.
- Keep the remaining sample identification numbers stapled to each sample order for use at CISM.
- Record the sample collection data in the **Health Unit sample record form** (current version of POP_MAL_001_A02_PT).
- After blood collection, the filter paper must be dried at room temperature for 24 hours, in a safe, dry, cool and ventilated place (air conditioning can be used or the windows of the room can be opened, depending on the conditions of the site).
  - The drying surface, which can be a bench, cabinet or shelf, must be easy to clean and disinfect;
  - Avoid direct exposure to the sun or heat;
  - Do not allow samples from different patients to overlap, to avoid contamination;
  - When the process of drying is complete, the dried blood spots will be darker than the fresh blood spots.
- Once drying is complete, place the two filter papers from the same patient in a small Ziplock bag.
- Samples will be placed in a large Ziplock bag containing silica gel and stored in a refrigerator with a temperature between 2 to 8°C until the date of shipment to CISM, Manhiça district.
- Record the date that the samples are stored in the Health Unit refrigerator in the **Health Unit sample record form** (current version of POP_MAL_001_A02_PT).
- The respective requisitions must be kept in plastic files to be sent simultaneously with the samples.

7.1.2. RDT blood sample collection

- Blood collection for the RDT will be carried out following the **Procedures for performing the malaria rapid diagnostic test** (current version of POP_LB_PT_012_PT), also considering the manufacturer's specific instructions; do not discard the silica gel bag in the RDT package.
- Stick the sample identification number on the RDT and the same sample identification number on the **Laboratory analysis requisition form** (current version of POP_MAL_001_A01_PT). Write the collection date and study acronym on the RDT using a pen.
- Keep the RDT in an individual Ziplock and add the silica gel bag.
- The RDTs will be placed in a large Ziplock back and stored in a refrigerator between 2 to 8°C until the shipment date to CISM, Manhiça district.
- Record the storage of the samples on the **Health Unit sample record form** (current version of POP_MAL_001_A02_PT).
- Keep the remaining sample identification numbers stapled to each sample requisition for use at CISM.

7.2 Transport of filter papers to the CISM Laboratory

- Study personnel will contact the CISM study leader to prepare the shipment.
- Shipment logistics will be organised as follows:

  For land transport:
  - ambient temperature
  - the samples and documents will be placed in cases that must be exclusively used for this purpose (**Figure 2**)

  For air transport:
  - preferably using Portador Diário (https://www.portadordiario.co.mz/).
  - ambient temperature
  - the person responsible for the study will record the shipping code that will be assigned to the samples for later use at the time of collection at the final destination, as well as to monitor the location of the samples along the way
- At least one day before transport, verify the agreement between the actual number of samples and the records in the **Health Unit sample record form** (current version of POP_MAL_001_A02_PT).
- Whenever possible, the plastic boxes for transporting samples should be sanitised before and after use with soap and water, then disinfected with 70% alcohol.
- On the arranged day of transport, place samples (filter papers or RDTs), the requisition forms, the control forms and other study-specific documents in the shipping boxes.

For peer review only - http://bmjopen.bmj.com/site/about/guidelines.xhtml

Figure 2. Case for transporting samples (filter papers and RDTs) by land

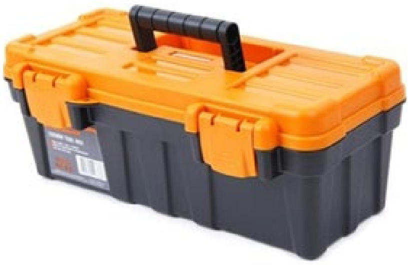

7.3 Receipt of samples at CISM

- Dried blood samples on filter paper for RDTs will be received at the CISM Laboratory, along with a laboratory requisition form.
- The Laboratory reception will verify the agreement between the sample identification numbers of the samples and the respective laboratory requisitions, and whether the number of samples received corresponds to the number of requisition forms.
- After verifying that everything is in order, the request will be entered into the SERVOLAB system, if not, the coordinator responsible must be informed so that they may follow up until the situation is resolved.

> NOTE: Samples without a laboratory requisition from partners will go through the laboratory reception for verification, however, these will not be entered into SERVOLAB due to insufficient data. The verification of these samples will be carried out together with the person responsible for the study, who must fill out the Excel Database **Control of samples in the laboratory** (current version of POP_MAL_001_A05_PT). This Excel document will be archived in electronic format and shared with the study team.

7.4 Storage of samples in the molecular biology laboratory

7.4.1. Storage of filter papers

- For storage, the two filter papers in each bag will be wrapped with aluminium foil and will be properly identified with the study name, bag number and group (A or B) using a permanent marker; the sample identification number will be stuck onto the aluminium foil;
- Place samples A in a large Ziplock bag (up to 100 filter papers) and samples B in a B Bag (up to 100 filter papers); then add 100g of silica gel to each bag (**Figure 3**).
- During the wrapping process, the **Filter paper placement form** (current version of POP_MAL_001_A03_PT) will be filled out simultaneously, which will then be verified by the technician responsible.
- The bags will be identified externally with the study name, bag group number (A or B) using a permanent marker and a paper containing the same information will be placed inside the bag.

Figure 3. Identification and storage of filter papers in the laboratory

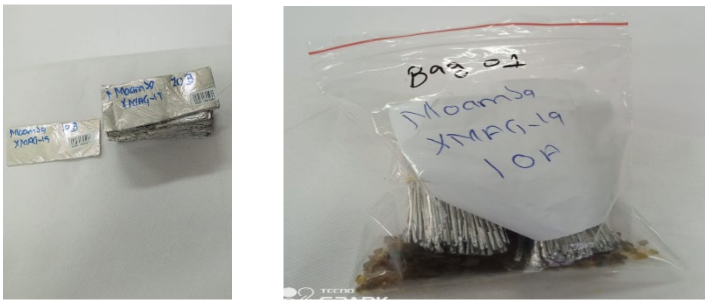

- Store A and B bags in a -20 degree freezer.

For peer review only - http://bmjopen.bmj.com/site/about/guidelines.xhtml

- The placement of the filter papers must be indicated in SERVOLAB (Seroteca Servolab>Type of Box 10x10->Box Name->filter paper bag X->placement).
- Lastly, fill out the Excel Database **Control of samples in the laboratory** (current version of POP_MAL_001_A05_PT).

7.4.2. Storage of RDTs

- For storage, the RDTs will be wrapped with aluminium foil and a sample identification number will be stuck onto the aluminium foil.
- During the wrapping process, the **RDT placement form** (current version of POP_MAL_001_A04_PT) will simultaneously be filled out, and then checked by the technician responsible.
- Place the samples from the same placement sheet in a bag (20 RDTs), then add 20g of silica gel.
- The bag will be identified externally with the study name, bag group number using a permanent marker and a paper containing the same information will be placed inside the bag.
- Place the bag of samples in a -20 degree freezer.
- The placement of the RDTs must be indicated on SERVOLAB (Seroteca Servolab>Type of Box 10x10->Box Name->RDT bag X->placement).
- Lastly, fill out the Excel Database **Control of samples in the laboratory** (current version of POP_MAL_001_A05_PT).

For peer review only

Appendix 10. COVID-19 safety and research considerations.

1. COVID19-related biosafety capacities: This project will not involve the use of SARS-CoV-2 for any purpose, as we will focus on the detection of malaria molecular markers for surveillance and research purposes. The only samples that will be collected and managed in this project will be dried blood spots, obtained from individuals in the community and pregnant women at antenatal clinics, which will minimize the risk of COVID-19 infection among health workers and laboratory staff during sample collection and processing, respectively. All personnel involved in the study will be trained in the most up to date Malaria Consortium procedures for infection prevention and control. A daily monitoring of the health personnel involved will be conducted through the measurement of axillary temperature and identification of respiratory symptoms. In case of clinical signs, domiciliary isolation and COVID-19 testing will be recommended. CISM has developed a biosafety plan considering the following considerations:

Before starting any project-related activity, a new risk assessment will be completed using the template provided by WHO at their last version of the "Laboratory biosafety guidance related to coronavirus disease (COVID-19): interim guidance" (https://apps.who.int/iris/handle/10665/331500).

2. Collection of specimens: Finger or heel prick bloods will be collected from pregnant women at antenatal clinics and individuals in the community by community health workers. No nasopharyngeal nor oropharyngeal swabs will be collected. Samples will be collected following biosafety WHO guidelines (use of personal protective equipment [PPE]: N95 or KF94 mask, disposable gloves, protective clothing, eye protection and frequent hand washing) as described in WHO guidance on specimen collection, processing and laboratory testing: https://www.who.int/publicationsdetail/laboratory-testing-for-2019-novel-coronavirus-in-suspected-human-cases-20200117, and biosafety procedures https://apps.who.int/iris/bitstream/handle/10665/331138/WHO-WPE-GIH-2020.1-eng.pdf.

3. Laboratory biosafety: All biological samples for molecular assays will be managed at CISM. Given the nature of the samples (dried blood spot), there is a minimal risk of producing aerosols. In general, de-capping is considered a low-risk procedure. However, it depends on the design of the lid and container. Whether to proceed with the testing will be determined following a risk assessment, which considers the need for centrifugation, mixing, and aliquoting. In addition, the use of a BSC will be considered at any time when there is a high risk. All risky procedures will be carried out in a validated class II Biosafety cabinet.

4. Emergency/incident response plan: Contingency plans will be developed to reduce the likelihood of exposure to/release of a biological agent, or to reduce the consequences of such incidents by providing specific standard operating procedures (SOPs) to be followed in possible emergency scenarios that apply to the work and local environment. Personnel will be trained on these procedures and have periodic refresher training to maintain competency. First-aid kits, including medical supplies such as bottled eye washes and bandages, will be available and easily accessible to personnel. All incidents will be reported to the appropriate personnel in a timely manner. A written record of accidents and incidents will be maintained. Any incident will be reported and investigated in a timely manner and used for updating laboratory procedures and emergency response plans. Spill kits, including disinfectant, will be easily accessible to personnel. Written procedures for cleaning and decontaminating spills will be developed for the laboratory and followed by suitably trained personnel.

5. COVID-19 prevention: To avoid contamination and or and the spread of the infection, all field personnel will be provided with personal protective material for COVID 19, including face masks and/or visors and alcohol gel. Soap will also be distributed to the health facilities for use by patients.

For peer review only - http://bmjopen.bmj.com/site/about/guidelines.xhtml

Appendix 11. Worksheet for monitoring and evaluation of field activities.

**Date**	
**Site**	
	Field
	Health Facility
**Samples**	
	Filter paper
	Rapid diagnostic test
**Quality**	
	Quality of the filter paper
	Witness the collection process if applicable
	Quantity of blood collected
	Quality of the blood collected
	Identification of the samples
**Cross-check sample data vs questionnaire and control sheet**	
	Compatible identification
**Information in source document**	
	Legibility of Information
	Data filled in the right place
	Confirm data with original document if applicable
**Total number of documents**	
	Questionnaire
	Informed consent
	Requisition form
**Total number of samples**	
	Filter paper
	RDT
	Number of discrepant RDT results
**Number of non-compliant documents**	
	Questionnaire
	Informed consent
	Requisition form
**Quantity of samples with non-conformity**	
	Filter paper
	RDT
**Evaluated by**	
	Name
	Date
**Revised by**	
	Name
	Date of the next monitoring

For peer review only - http://bmjopen.bmj.com/site/about/guidelines.xhtml

BMJ Open

Protocol for a prospective surveillance study to detect antimalarial drug resistance, gene deletions of diagnostic relevance and genetic diversity of Plasmodium falciparum in Mozambique

Journal:	*BMJ Open*
Manuscript ID	bmjopen-2022-063456.R1
Article Type:	Protocol
Date Submitted by the Author:	27-May-2022

Complete List of Authors:	Mayor, Alfredo; Centro de Investigação em Saúde de Manhiça; ISGlobal<br>da Silva, Clemente; Centro de Investigação em Saúde de Manhiça<br>Rovira-Vallbona, Eduard; ISGlobal<br>Roca-Feltrer, Arantxa ; Malaria Consortium<br>Bonnington, Craig; Malaria Consortium<br>Wharton-Smith, Alexandra; Malaria Consortium<br>Greenhouse, Bryan; University of California San Francisco, Medicine<br>Bever, Caitlin; Bill & Melinda Gates Foundation<br>Chidimatembue, Arlindo; Centro de Investigação em Saúde de Manhiça<br>Guinovart, Caterina; Barcelona Institute for Global Health<br>Proctor, Josh; Bill & Melinda Gates Foundation<br>Rodrigues, Maria; Malaria Consortium<br>Canana, Neide; Malaria Consortium<br>Arnaldo, Paulo; Instituto Nacional de Saúde<br>Boene, Simone; Centro de Investigação em Saúde de Manhiça<br>Aide, Pedro; Centro de Investigação em Saúde de Manhiça<br>Enosse, Sonia; Malaria Consortium<br>Saute, Francisco; Centro de Investigação em Saúde de Manhiça<br>Candrinho, Baltazar; Ministry of Health

**Primary Subject Heading**:	Infectious diseases
Secondary Subject Heading:	Genetics and genomics, Public health, Epidemiology
Keywords:	Epidemiology < INFECTIOUS DISEASES, Molecular diagnostics < INFECTIOUS DISEASES, Public health < INFECTIOUS DISEASES, MOLECULAR BIOLOGY, PARASITOLOGY, Genetics < TROPICAL MEDICINE

SCHOLARONE™
Manuscripts

Protocol for a prospective surveillance study to detect antimalarial drug resistance, gene deletions of diagnostic relevance and genetic diversity of *Plasmodium falciparum* in Mozambique

Alfredo Mayor[1,2,3,4], Clemente da Silva[1], Eduard Rovira-Vallbona[2], Arantxa Roca-Feltrer[5], Craig Bonnington[5], Alexandra Wharton-Smith[5], Bryan Greenhouse[6], Caitlin Bever[7], Arlindo Chidimatembue[1], Caterina Guinovart[2], Josh Proctor, Maria Rodrigues[5], Neide Canana[5], Paulo Arnaldo[8], Simone Boene[1], Pedro Aide[1], Sonia Enosse[5], Francisco Saúte[1], Baltazar Candrinho[9]

1. Centro de Investigação em Saúde de Manhiça (CISM), Manhiça, Mozambique
2. ISGlobal, Hospital Clínic - Universitat de Barcelona, Barcelona, Spain
3. Department of Physiologic Sciences, Faculty of Medicine, Universidade Eduardo Mondlane, Maputo, Mozambique
4. Spanish Consortium for Research in Epidemiology and Public Health (CIBERESP), Madrid, Spain
5. Malaria Consortium, Maputo, Mozambique
6. University of California San Francisco, USA
7. Institute for Disease Modeling, Bill and Melinda Gates Foundation, USA
8. Instituto Nacional de Saúde, Maputo, Mozambique
9. National Malaria Control Program, Ministry of Health, Mozambique

Corresponding autor: Alfredo Mayor, Centro de Investigação em Saúde de Manhiça (CISM), Maputo, Moçambique. Telephone (+ 258) 21 810 002; e-mail: alfredo.mayor@isglobal.org

Key words: Malaria, genomics, pregnancy, next generation sequencing, surveillance

Abtract: 303
Text: 4286

For peer review only

Abstract

Introduction

Genomic data constitutes a valuable adjunct to routine surveillance that can guide programmatic decisions to reduce the burden of infectious diseases. However, genomic capacities remain low in Africa. This study aims to operationalize a functional malaria molecular surveillance system in Mozambique for guiding malaria control and elimination.

Methods and analyses

This prospective surveillance study seeks to generate *P. falciparum* genetic data to 1) monitor molecular markers of drug resistance and deletions in rapid diagnostic test targets; 2) characterize transmission sources in low transmission settings; and 3) quantify transmission levels and the effectiveness of antimalarial interventions. The study will take place across nineteen districts in nine provinces (Maputo city, Maputo, Gaza, Inhambane, Niassa, Manica, Nampula, Zambézia and Sofala) which span a range of transmission strata, geographies and malaria intervention types. Dried blood spot samples and rapid diagnostic tests will be collected across the study districts in 2022 and 2023 through a combination of dense (all malaria clinical cases) and targeted (a selection of malaria clinical cases) sampling. Pregnant women attending their first antenatal care visit will be also included to assess their value for molecular surveillance. We will use a multiplex amplicon-based next generation sequencing approach targeting informative single nucleotide polymorphisms, gene deletions and microhaplotypes. Genetic data will be incorporated into epidemiological and transmission models to identify the most informative relationship between genetic features, sources of malaria transmission and programmatic effectiveness of new malaria interventions. Strategic genomic information will be ultimately integrated into the national malaria informationand surveillance system to improve the use of the genetic information for programmatic decision-making.

Ethics and dissemination

The protocol was reviewed and approved by the institutional (CISM) and national ethics committees of Mozambique (Comité Nacional de Bioética para Saúde) and Spain (Hospital Clinic of Barcelona). Project results will be presented to all stakeholders and published in open-access journals.

Study registration number: ClinicalTrials.gov NCT05306067

For peer review only

Strengths and limitations of this study

- Next generation sequencing will be performed in country through the establishment of technical and computational infrastructure as well as analytical tools.
- The project builds from recent elimination experiences in southern Mozambique and uses a biorepository of already collected *P. falciparum* samples to select multi-allelic short-range haplotypes (microhaplotypes) that increase the power of biallelic loci for phase inference in polygenomic infections.
- A joint epidemiological-genetic analysis will enable better predictions of the operational efficacy of new interventions.
- We will assess the value of a new surveillance systems at antenatal visits to improve the programmatic performance of malaria control and elimination activities.
- More evidence on the association between malaria transmission intensity and genetic data is required for the use of malaria molecular surveillance data to assess the effectiveness of malaria interventions.

Introduction

Pathogen genomics has the potential to transform the surveillance, prevention and control landscape of infectious diseases. The rapid innovation in sequencing technologies has led to the development of robust next-generation sequencing (NGS) equipment with the ability for high pathogen resolution at increasingly affordable prices. This development has subsequently facilitated the incorporation of pathogen genomics in disease surveillance systems in high-income countries, allowing for targeted and effective control of disease threats through the timely and in-depth pathogen characterisation[1]. Genomics-based surveillance is therefore becoming an integral strategy towards control and elimination of diseases such as COVID19, tuberculosis, malaria, HIV and foodborne pathogens, among others[2].

The strategic use of genetic variation in *P. falciparum* can boost the capacity of malaria control and elimination programs to deploy the most efficient interventions[3]. Molecular tools and use cases for decision making are currently being considered by the World Health Organization (WHO) which, through a technical consultation on the role of parasite and anopheline genetics in malaria surveillance[4], identified different levels of action based on evidences available. Genetic data can flag the emergence of mutations conferring resistance to antimalarials (i.e., artemisinins)[5] or deletions that affect rapid diagnostic test (RDT) sensitivity (i.e., *P. falciparum histidine-rich protein 2* [*pfhrp2*])[6-8]. Genomic scans for selection[9] can identify other parasite adaptations mediated by single nucleotide polymorphisms (SNPs) and structural variations (gene copy number[10]) that may require a programmatic response. Parasite relatedness metrics such as identity by descent (IBD)[11] can be used to characterize the key drives of ongoing transmission, to identify foci[12 13] and to discriminate between indigenous and imported cases in areas approaching elimination[14-16]. Bottlenecks in parasite population driven by control and elimination efforts have been shown to reduce *P. falciparum* genetic diversity and increase similarity due to inbreeding and recent common ancestry[17]. These evidences provide the basis for modelling efforts to recapitulate features of malaria transmission from genetic data and inform about the effectiveness of antimalarial interventions[18-23]. However, further evidence is needed to demonstrate the feasibility and appropriateness of using genetic data as a proxy for transmission intensity and define the conditions under which that feasibility applies. Moreover, standardised approaches for detecting resistance through molecular markers are lacking, and variation in sample type, collection, storage, DNA extraction, marker detection and analysis of results can undermine the comparability of findings, as well as the sensitivity and specificity of methods used. Adequate genotyping methods, sampling frameworks, analytical pipelines and demonstration studies are still required across a range of malaria intensities, programmatic environments and use scenarios.

Strategic *P. falciparum* genetic information can be integrated into innovative cost-efficient surveillance approaches, such as those targeting pregnant women attending antenatal care (ANC) clinics[24]. Women at ANC are a generally healthy, easy-access population, contributing valuable data for infectious disease surveillance (ie, HIV[25] and syphilis[26]) and wider health metrics at the community level, including a proxy of the malaria burden in the community[27-32]. Moreover, ANC-level malaria surveillance can provide a routine measure of the malaria burden in pregnancy, which countries lack, whilst potentially improving pregnancy outcomes by treating infections at first trimester. Women attending ANC also provide an attractive sampling population for measures of exposure to malaria beyond simply presence or absence of parasite infection. In particular, in

For peer review only

addition to measuring complexity of infection or parasite flow-rates between populations, molecular analysis of *P. falciparum* isolates collected from pregnant women may provide a means for the identification of adaptations developed by the parasite to control strategies, such as antimalarial resistance and deletions of antigens targeted by rapid diagnostic test that can compromise diagnosis, treatment and prevention.

Despite the potential benefits and the greater need to control the high burden of infectious diseases, genomic surveillance capacity remains low for many public health programmes in Africa[2]. In order to reduce inequities in the access to sequencing technologies, this project aims to promote capacities in Mozambique for operationalizing a functional malaria molecular surveillance (MMS) system for decision making[4]. Mozambique is among the ten countries with the highest burden of malaria worldwide, with an estimated 10.8 million cases in 2020[33]. However, malaria transmission is very heterogeneous in the country, with a high burden in the north and very low transmission in the south. Therefore, the project aims to address National Malaria Control Program (NMCP) programmatic needs for elimination initiatives in southern Mozambique and burden reduction in the north (**Figure 1**).

Methods and analysis

Study design

This is a prospective genomic surveillance study of *P. falciparum* isolates to be collected between 2022 and 2023 from a variety of transmission intensities and geographies in Mozambique to inform three use cases: appropriate malaria diagnostics and treatment; characterizing transmission sources in low transmission settings; and identifying intervention mixes with optimal effectiveness to reduce burden in moderate-to-high transmission areas. To achieve this, three different sampling approaches will be performed. First, all malaria cases will be sampled throughout the year in two low transmission districts of southern Mozambique currently targeted by reactive malaria surveillance activities (*dense sampling).* Second, a targetted approach will aim to collect a predefined number of samples at selected health facilities in the country. In low transmission settings, sampling will be conducted throughout the year, while two surveys will be conducted in medium-to-high transmission settings: one during the rainy and a second one during the dry season (which extend from November to April and May to October, respectively). During the high transmission (rainy) season, an LDH-based RDT will be added to the standard routine HRP2-based diagnostics to identify potential false negative results due to *pfhrp2/3* deletions among clinical cases[34]. And third, *ANC sampling* of pregnant women at first attendance will be conducted throughout the year at selected health facilities in the country. The overarching sampling strategy for the study will however remain flexible and iterative, informed by sample analysis as the study progresses, and in view of future sampling and research activities being conducted by the Ministry of Health, National Institute of Health and other stakeholders in Mozambique, to avoid sampling overlap and ensure a diversity of sampled locations.

The project will also leverage from clinical trials and surveillance activities being conducted in Mozambique between 2021 and 2024, namely: the Malaria Indicator Survey (2022-2023) in southern Mozambique; the therapeutic efficacy survey (2022) in sentinel sites in the country (Montepuez in Cabo Delgado, Moatize in Tete, Dondo in Sofala, Mopeia in Zambézia and Massinga in Inhambane)[35 36]; reactive surveillance activities in Magude and Mautuine (Maputo Province); a Phase III cluster-randomized, open-label, clinical trial in 2022 to study the safety and efficacy of ivermectin mass drug administration to reduce malaria transmission in Mopeia District (Zambeia Province); a large-scale implementation development project aiming at maximising the delivery and uptake of intermittent preventive treatment in infancy (IPTi) in Massinga District (Inhambane; 2022-2024); a hybrid effectiveness-implementation study to evaluate the feasibility and effectiveness of seasonal malaria chemoprevention (SMC) with sulfadoxine-pyrimethamine and amodiaquine in Lalaua and Muecate districts (Nampula Province; 2022); and a programmatic delivery of a population-based mass drug administration with dihydroartemisinin-piperaquine in Manjacaze district (Gaza Province; 2022-2023).

Study settings and participants

Eight provinces were identified through consultation with the NMCP for inclusion in the study: Maputo, Gaza, Inhambane, Niassa, Manica, Nampula, Zambezia and Sofala. Selection of study sites will be stratified by transmission intensity into two major strata: A) low transmission (Maputo city and Maputo Province, where

For peer review only

individual case notification is being implemented to reach interruption of transmission), and B) medium-to-high transmission areas (Gaza, Inhambane, Niassa, Manica, Nampula, Zambezia and Sofala provinces, targeted by burden-reducing strategies). Overall, a total of 19 districts will be included, which provide a diverse range of epidemiological settings (see **Table 1** and **Figure 2**).

Table 1. Study provinces and districts targeted in the protocol.

				Sampling			
				Dense	Targeted		Other sources
Transmission	Region	Province	District		HFS	ANC	
Low	South	Maputo City	Kamavota		X^1		
			KaMaxaqueni		X^1		
			Nlhamankulu		X^1		
		Maputo Province	Boane		X^1		
			Manhiça		X^1		
			Magude	X^1		X^2	React
			Matutuine	X^1			React
Medium-to-high		Gaza	Manjacaze		X^3	X^2	MDA-DP
		Inhambane	Maxixe		X^3	X^2	
			Massinga		X^3	X^2	IPTi & TES
	Central	Manica	Guro & Gondala		X^3	X^2	
		Sofala	Chemba		X^3	X^2	
			Dondo				TES
		Tete	Moatize				TES
	North	Niassa	Cuamba		X^3	X^2	
		Nampula	Mecuburi & Malema		X^3	X^2	
			Lalaua & Muecate		X^3	X^2	SMC
		Zambezia	Mopeia		X^3	X^2	MDA-IVM & TES
		Cabo Delgado	Montepuez				TES

ANC, Antental care clinics; HFS, health facility survey; IPTi, intermittent preventive treatment in infancy; MDA-DP, Mass drug administration with dihydroartemisinin-piperaquine; MDA-IVM, Mass drug administration with Ivermectin; REACT, Reactive surveillance; SMC, seasonal malaria chemoprevention; TES, Therapeutic efficacy study.

1, Year round, all ages
2, Year round, first ANC visit
3, Rainy & Dry season; 2-10 years of age

Dense sampling will be conducted in the low transmission districts of Magude and Matutuine, where all the individuals of any age (>6 months old) with clinical symptoms of malaria (defined as axillary temperature ≥37.5ºC or history of fever in the preceding 24 hours) and a parasitologically confirmed malaria diagnosis via RDT or microscopy (**Table 2**) will be invited to donate their RDT for molecular analysis (dense sampling).

For peer review only - http://bmjopen.bmj.com/site/about/guidelines.xhtml

Table 2. Study eligibility criteria

INCLUSION CRITERIA	EXCLUSION CRITERIA
**Low transmission**	
- Any age	- Any symptoms of severe malaria
- Fever (axillary temperature ≥37.5ºC) or history of fever in the preceding 24 hours	- Negative parasitological test for malaria via RDT or microscopy (except any women at their first ANC visit, who will be recruited before testing for malaria with an RDT)
- Positive parasitological test for malaria diagnosis via RDT or microscopy	- Unwilling to provide informed, written consent
- Household contact of someone with fever/history of fever and *P. falciparum* positive RDT	- History of antimalarial treatment in the last 14 days
OR	
- Pregnant women attending first antenatal care visit in Magude district	
AND	
- Informed, written consent to participate from participant and/or guardian	
**High transmission**	
- Children aged 2-10 years of age	- Any symptoms of severe malaria
- Fever (axillary temperature ≥37.5ºC) or history of fever in the preceding 24 hours	
- Positive parasitological test for malaria diagnosis via RDT* or microscopy	- Negative parasitological test for malaria via RDT or microscopy (except any women at their first ANC visit, who will be recruited before testing for malaria with an RDT)
OR	
- Pregnant women attending first antenatal care visit	- Unwilling to provide informed, written consent
AND	- History of antimalarial treatment in the last 14 days
- Informed, written consent to participate from participant and/or guardian	

*a second RDT (HRP2-pLDH) will be provided in these locations to support detection of *P. falciparum hrp2* deletions.

review only

Targetted sampling will be conducted at selected health facilities in the low transmission districts of Boane, Manhiça and Maputo City (KaMavota, KaMaxaqueni and Nhamankulu Districts), where a drop of blood will be collected onto filter paper from consenting individuals of any age (>6 months old) with confirmed clinical malaria. In medium-to-high transmission areas, targeted sampling will focuss on children aged 2-10 years of age attending selected health facilities with clinical symptoms of malaria and a parasitologically confirmed malaria diagnosis via RDT (**Table 2**). Ten health facilities will be targeted in each district.

Pregnant women attending their first antenatal care visit (any trimester) will be invited to participate both in low (Magude in Maputo Province) and high transmission districts (Maxixe in Inhambane, Manjacaze in Gaza, Mecuburi and Malema in Nampula, Cuamba in Niassa, Guro and Gondala in Manica, Chemba in Sofala), irrespectively of malaria clinical symptoms.

Enrolment of participants

Dense sampling in Magude and Matutuine districts will be coordinated with district malaria focal points, community health workers (CHW), malaria volunteers (who provide a link between the CHW and the health facility, and assist the CHW in the follow-up of cases and administration of medication) and health facilities. All *P. falciparum* positive RDTs (SD Bioline Malaria Ag Pf, 05FK50, Abbott) will be stored for molecular analysis. RDTs of *P. falciparum*-confirmed household contacts will be also collected to estimate the rate of within-household transmission. Targeted sampling through health facility-based surveys (HFS) in low and medium-to-high transmission settings will be carried out by one team comprised of one maternal and child

For peer review only - http://bmjopen.bmj.com/site/about/guidelines.xhtml

health nurse, a laboratory technician or a medical technician. The number of people to be screened in each health facility and the duration of recruitment to achieve the sample size will be dependent on the RDT-positivity rate among people meeting the eligibility criteria. A second test including a non-HRP2 line (StandardQ Malaria Pf/Pan Ag Test, SD Biosensor) will be carried out in HFS during the rainy season and discrepant results suggestive of *pfhrp2/3* deletions will be recorded and further analysed to confirm the deletion. Nurses at the ANC clinics will be in charge of the recruitment of pregnant women at their first visit. Pregnant women will be tested for malaria using a routine RDT and the result will be recorded in a standard questionnaire, together with routine ANC tests. Each enrolled individual will be assigned with a unique identification (UID) number and a barcode.

Data and sample collection

Field workers and nurses will be trained to ask for informed consent (**Appendix 1-4**), perform a simple questionnaire (**Appendix 5-8**) and collect biological samples for molecular analysis. The survey questionnaire will be administered to all study participants or children's parents/guardians meeting the inclusion criteria and will include inclusion criteria check, characteristics of the participant and malaria related information. For pregnant participants, data will be collected on parity and gestational age at first ANC visit, as well as information related with malaria and use of preventive measures. A telephone contact number will be collected from pregnant women in low transmissions settings in order to locate their residence for spatial analysis. In areas targeted by reactive surveillance activities (Magude and Matutuine in Maputo Province), travels during the previous 30 days to the case notification are registered, including destinations and dates. A Site Coordinator will be responsible for supervising the work of field workers, nurses and the data entry clerk, and for reviewing and comparing questionnaires and samples for correct matching, completeness and accuracy.

Nurses will be trained to collect blood by finger pricking (**Supplemental Table 1**) following standard (**Supplementary Appendix 9**) and COVID19 safety procedures (**Supplementary Appendix 10**). For each participant, either the *P. falciparum*-positive RDT used for routine malaria diagnosis (dense sampling) or four blood spots onto two filter papers (Whatman® 3MM; targeted sampling) will be collected. Specimens will be labelled anonymously (patient UID, study health facility and date), dried for 24 hours and kept in individual plastic bags with desiccants at 4ºC. Every two to six weeks, the completed questionnaires, informed consents and samples will be sent to the data entry clerk at CISM through a local transportation agency. Informed consents will be received by study investigators. A data manager will be responsible for the receipt of the informed consents and double data entry at CISM, and a laboratory technician will be responsible for receiving the samples and store them at -20ºC until analysis. Part of the dried blood spot will be stored in RNA-preserving solution. All samples will be kept in the CISM Laboratory for a period of approximately 15 years. For quality control purposes, up to 5% of the samples will be analyzed at UCSF (San Francisco, USA) and/or ISGlobal (Barcelona, Spain). In order to identify errors in data or sample collections and take necessary corrective actions, a standardized checklist (**Supplementary Appendix 11**) will be filled in by the monitoring officer during biweekly monitoring visits.

Molecular analyses

Informative SNPs (including -but not restricted to- markers of resistance to artemisinin [*pfkelch13*][37], sulfadoxine-pyrimethamine [*pfdhfr, pfdhps*][38], or chloroquine [*pfcrt*][39]), microhaplotypes[40] and *pfhrp2* and *pfhrp3* regions[6-8]) will be targeted using multiplexed primers on flanking sequences, with a range of amplicon size of ~225-275 bp (covered by a paired end read). Targeted amplicons obtained by PCR on genomic DNA using Illumina-specific adaptors and sample-specific barcode will be pooled to create a single product library, which will be sequenced (paired-end 150-bp) on a Miseq Illumina sequencer in the country or higher performing equipment when available. Amplicon representation and SNP and haplotype calling will be assessed in demultiplexed and trimmed sequencing reads after filtering sequencing errors. The designed panel will be validated using mixtures of *P. falciparum* lines to determine precision and repeatability. Genotyping methods, including number of SNPs and microhaplotypes to be characterized, distribution across the parasite's chromosomes, the proportion of putatively neutral vs. non-neutral polymorphisms, pooling strategy and criteria for validating sequencing data (i.e., minimum sequencing depth and maximum error rate) will be developed as part of this project. Samples will be also used for other molecular analysis of programmatic interest, such as the detection of *Plasmodium* species, parasite antigens, serological markers of parasite exposure (antibodies) and parasite RNA-based markers (i.e., gametocytes). A quality control program based

For peer review only - http://bmjopen.bmj.com/site/about/guidelines.xhtml

on the sequencing of an artificially-created set of samples (i.e. mixtures of known laboratory controls at specific proportions and densities) will be processed at predefined times to guarantee the quality of the processes during the life of the project.

Data Management

Data will be collected using paper (targeted sampling) and password-protected electronic devices (dense sampling). Data collected using paper will be double entered into the study database using RedCap[41]. Automatic quality checks will be performed to ensure data completeness. Confidentiality and security will be ensured through automatic encryption of sensitive data, storage in password protected computers and locked locations, and data sharing using password-protected, encrypted files. Prior to analysis, data will be de-identified with the exception of geo-location codes, which are necessary for specific analyses. The study will also use data available from the NMCP, including intervention coverage, historical prevalence surveys, travel history or other mobility assessments, and entomological data. Sequences generated through the analysis of samples will be integrated into a curated catalogue of genomic data together with relevant anonymized clinical and epidemiological information and will be made publicly available in public repositories such as the European Nucleotide Archive (ENA) and MalariaGen Resource Center. In order to facilitate data accessibility and use, and to obtain a meaningful integration with other sources of surveillance data, genetic information will be incorporated into the DHIS2-based Integrated malaria information storage system (iMISS), which is currently being rolled out in Mozambique[42].

Study outcomes and sample size calculations

The primary endpoints are: a) Prevalence of molecular markers of diagnostic and antimalarial resistance by period, study area and population (use case 1); b) Genetic relatedness indicators between pairs of samples and populations by period, study area and population (use case 2); and c) Genetic diversity indicators by period, study area and population (use case 3). Sample size per sampling domain (Province) has been estimated considering antimalarial and diagnostic resistance as a primary use case, considering the negligible carriage of molecular markers of artemisinin resistance[5] and *pfhrp2/3* deletions[6] in Mozambique, and setting 5% as the warning threshold [43]. Assuming a 10% of loss of samples or uninterpretable analysis, a sample size of up to 500 per sampling domain would be adequate to: a) estimate a proportion of 0.05 (markers of drug resistance or *pfhrp2* deletion) with 0.026 absolute precision and 95% confidence and b) achieve a power of 80% for detecting an increase of genetic marker (resistance or deletion) from 0 ‰ to 5% at a two-sided p-value of 0.01. A flexible and adaptive sampling scheme will be followed, where a) estimates generated during the first half of the project will inform subsequent sampling schemes and b) not all the samples collected will be analysed (some of them will be stored as reference materials, for confirmation of findings or future studies on *Plasmodium* biology). The number of pregnant women to be recruited in order to reach the sample number will depend on the parasite rates in the study areas; assuming an overall RDT positivity rate of 25%, we expect we will be needing to recruit a total of 2,000 pregnant women per site to get 500 *P. falciparum* positive samples, although numbers may differ between sites.

Analysis Plan

Demographic and clinical charateristics of study participants will be described using summary statistics. A user-friendly and locally executable bioinformatic pipeline will be developed for analysis of *P. falciparum* targeted sequencing data. Highly informative SNPs and microhaplotypes showing geographic structuring will be selected using a supervised machine learning approach trained by genomes from known geographic origin in Mozambique. Population-level genetic diversity will be quantified using expected heterozygosity (He), number of alleles per locus, allele frequency, complexity of infection (COI)[23] as well as other genetic metrics. Deletions and copy number variations will be assessed based on sequencing coverage ratios[10 44]. Methods to be used for population genetic analysis, including the genetic connectivity among isolates, use of all versus only neutral SNPs, treatment of multiple-clone infections and integration of genetic data with travel history data) will be developed during the project. We will use regression models adjusted by potential confounders (demographic and clinical factors, among others) to compare genetic metrics between seasons, before and after the antimalarial interventions, between pregnant women and community sampling populations and across different intensities of malaria transmission. Finally, we will integrate genomic surveillance data into epidemiological and transmission network models. For the first one, we will leverage two recent models developed at the Institute of Disease Modelling[45] (a malaria genetic model calibrated to a longitudinal genetic

For peer review only - http://bmjopen.bmj.com/site/about/guidelines.xhtml

study in Senegal[18] and a disease transmission model calibrated with the Magude data) to build an end-to-end malaria transmission and genetics model for Mozambique (**Figure 3**). The transmission network model will include data for densely sampled in low transmission areas on individual and community-level case classification (imported, local, introduced), the extent and duration of sustained local transmission and how these change over space and time. Summary indicators will be visualized in graphical and tabular forms in the iMISS through genetic dashboards. We will establish risk profile algorithms and interpretation components that are capable of generating outputs on a) country-wide antimalarial resistance profiles (rolling-basis); b) in very low transmission areas (e.g. Magude district), genetic connectivity and case classification (together with travel history and other parameters obtained from case-based notification tools); and c) "high burden to high impact" specific analyses (i.e., stratification and trend investigation for exploring the potential impact of intervention mixes implemented).

Ethics and dissemination

The protocol was reviewed and approved by the institutional (CISM) and national ethics committees of Mozambique and the Hospital Clinic of Barcelona. Written informed consent will be sought from all study participants before blood sample collection is conducted (**Appendix 1)**. Two copies will be signed, one will be kept by participant and the other by the investigators in a locked space. The information sheet and consent form will also include text explaining informed consent for future use of biological specimens to conduct additional analyses of the *Plasmodium* parasite. In case of minors (less than 18 years of age), consent will be sought from parents, relatives or guardians. Informed consents will specify that the data will be made public. First line treatment for malaria will be provided to the enrolled participants in line with national treatment guidelines. Considerations related to preventing the risk of SARS-COV-2 transmission are detailed in **Supplementary Appendix 10**. There will not be any economic incentive to participate in the study. Transference of data and materials out of Mozambique will be done only when appropriate data and material transfer agreements are signed between participating institutions (**Supplementary Table 2**).

Patient and Public Involvement

Patients and the public were not involved in the development of this protocol.

Discussion

There is a growing acceptance that genomics can play a critical role in policy and programmatic decisions. With the aim of demonstrating the programmatic application and feasibility of malaria genomic surveillance in Mozambique, we will generate parasite genomic data across varying transmission scenarios for supporting strategic decision-making. First, MMS data will inform drug and diagnostic choices through the monitoring of molecular markers of antimalarial and diagnostic resistance. The emergence of *pfhrp2/3* deletions[6-8], resistance to artemisinin[37] and partner drugs, as well as the resistance to sulfadoxine-pyrimethamine (SP) used for intermittent preventive treatment (in both pregnancy and infancy) and seasonal malaria chemoprofilaxis[38 46 47], threatens the global effort to reduce the burden of malaria[33]. The WHO recommends that countries with reports of *pfhrp2/3* deletions, and neighbouring countries, should conduct representative baseline surveys among suspected malaria cases. If the prevalence of molecular markers of antimarial resistance or deletions causing false negative RDT results reaches the threshold of >5%, then there is need to consider alternative antimalarials and RDTs. Second, the project will help to target the reservoirs sustaining transmission by quantifying parasite importation, identifying sources and characterizing local transmission in near-elimination settings[48 49]. Genomic surveillance and phylogenetic analyses have enabled the near real-time estimation of transmission chains of non-sexually recombining, rapidly evolving pathogens such as Ebola[50], influenza[51] and COVID19[52]. However, molecular and analytic advancements are stil required to characterise transmission patterns of pathogens such as *P. falciparum* with a sexually recombining stage[49]. Third, the project will assess the value of *P. falciparum* genetic diversity measures to supplement traditional surveillance for improving stratification, monitoring and impact evaluations in different epidemiological contexts, especially where surveillance data is sparse. This use case still requires development of analytical and interpretative to infer malaria burden[18 20 53-58] and effectiveness of interventions[18-23 53 59-61], as well as validation of sampling frameworks[4]. Finally, the project will test if parasite populations within pregnant women are representative of the general population and expand the usefulness of this approach to inform genomic surveillance indicators.

For peer review only - http://bmjopen.bmj.com/site/about/guidelines.xhtml

The project will use state-of-the-art sequencing and modelling approaches. Current *P. falciparum* genetic markers based on biallelic SNPs have limited support for polyclonal samples, which are frequent across all transmission intensities, and have limited resolution to calculate genetic relatedness between parasites, to estimate allele frequencies[23 62], or to distinguish geographic origin[21 23 63]. Multi-allelic short-range haplotypes (microhaplotypes) covered by a single read from high-throughput DNA sequencers allow an accurate statistical inference of phase and have the potential to derive more accurate information than biallelic loci[64-66], particularly in polyclonal infections, to tailor the genomic tool to specific transmission and geographic settings. In addition to being useful for identification and lineage/family relationships, microhaps can provide information on biogeographic ancestry and can be useful for strain detection and deconvolution[64-67]. Methods such as IBD[11 68 69] that can exploit the signal left by recombination on these microhaplotypes may have the power to detect geographic differentiation at small spatial scales relevant for malaria control programs. Machine learning approaches[70] will be used for the selection of key SNPs and microhaplotypes that allow accurate inference of malaria transmission and geographical origin. Finally, models that integrate genomic and epidemiological data will be developed to assess the programmatic effectiveness of new malaria interventions and characterize sources of malaria transmission (imported versus local)[45].

This project, guided by programmatic priorities and based on collaborative efforts, aims to boost the use of the genetic data for decision making. To successfully achieve this, the project is grounded on three main principles: a) strengthen sequencing capacities to implement a robust MMS system; b) strong partnership and coordination to make MMS data sharing common practice for malaria control and elimination; and c) effective operationalization of MMS implementation activities. Technical capacities will be built by establishing at CISM a sequencing platform and ancillary equipment for library preparation and quality control. Computational infrastructure and analytical tools will be also developed by establishing a user-friendly automated platform to analyse genomic data with simplified interpretation into actionable information. Training activities will target molecular biologists for wet laboratory analysis, a bioinformatician and molecular epidemiologists for data analysis and interpretation and a field epidemiologist for interpretation of the generated data, and public health specialists for adoption of the findings into policy. Genetic data-to-action culture and engagement of NMCP on genetic analysis will be promoted by integrating genetic aspects in the NMCP activities (i.e., data review meetings) as well as in training and annual meetings, by integrating genetic information with other surveillance data onto the iMISS, and by documenting all the processes, successes and failures to inform future molecular activities. The project will pursue the use of MMS data as an adjunct to traditional surveillance information for elimination initiatives in southern Mozambique and burden reduction in the north through the engagement with regional malaria elimination initiatives (e.g. E8 and MOSASWA, a trilateral initiative to eliminate malaria from Mozambique, South Africa, and Swaziland[7172]) and linking decision making with the 'high burden to high impact' initiative under the guidance of WHO.

We expect that the genomic intelligence developed through this project will complement current and new surveillance systems to drive decision-making for the control and eventual elimination of malaria in Mozambique and other malaria endemic countries. However, further steps are required beyond this three-year project. Enabling policies and regulatory mechanisms for sample storage and sharing[73], adequate procurement of materials and infrastructure, as well as local expertise for equipment installation and maintenance, need to be developed for an effective integration of genomic surveillance into public health. Countries, with appropriate support from mainstream funding bodies, should also develop sustainability plans as part of national disease control programmes, emergency responses, and other surveillance programmes (i.e., antimicrobial resistance) to ensure resources for genomic surveillance. Finally, regular assessments of the efficiency and effectiveness of incorporating genomic data in routine public health surveillance systems will be crucial to stimulate the use of genetic data for policy making.

Ethics approval: The protocol has been approved by the institutional (CISM; Ref: CIBS-CISM/044/2021) and national (Ref: 604/CNBS/21) ethics committees of Mozambique and the Hospital Clinic of Barcelona (Ref. HCB/2022/097).

Authors' contributions: Conceived and designed the protocol: AM, CB, ARF, BG. Gave inputs to protocol methodology: BC, CG, AC, ERV, CS, FS, SE, AWS, PAr, SB, MR, NC, PAi, JP. Wrote the first draft of the

For peer review only - http://bmjopen.bmj.com/site/about/guidelines.xhtml

manuscript: AM. Wrote, reviewed and approved the manuscript: all authors. Responsible for the overall content: AM.

Funding statement: This work is supported by Bill and Melinda Gates Foundation grant number INV-019032.

Competing interests: There are no competing interests for any author.

For peer review only

References

1. Armstrong GL, MacCannell DR, Taylor J, et al. Pathogen Genomics in Public Health. *N Engl J Med* 2019;381(26):2569-80. doi: 10.1056/NEJMsr1813907
2. Inzaule SC, Tessema SK, Kebede Y, et al. Genomic-informed pathogen surveillance in Africa: opportunities and challenges. *Lancet Infect Dis* 2021;21(9):e281-e89. doi: 10.1016/S1473-3099(20)30939-7 [published Online First: 20210212]
3. Tessema SK, Raman J, Duffy CW, et al. Applying next-generation sequencing to track falciparum malaria in sub-Saharan Africa. *Malar J* 2019;18(1):268. doi: 10.1186/s12936-019-2880-1 [published Online First: 2019/09/04]
4. WHO. Meeting report of the technical consultation on the role of parasite and anopheline genetics in malaria surveillance 2019 [Available from: https://www.who.int/publications/m/item/meeting-report-of-the-technical-consultation-on-the-role-of-parasite-and-anopheline-genetics-in-malaria-surveillance.
5. Gupta H, Macete E, Bulo H, et al. Drug-Resistant Polymorphisms and Copy Numbers in Plasmodium falciparum, Mozambique, 2015. *Emerg Infect Dis* 2018;24(1):40-48. doi: 10.3201/eid2401.170864 [published Online First: 2017/12/21]
6. Gupta H, Matambisso G, Galatas B, et al. Molecular surveillance of pfhrp2 and pfhrp3 deletions in Plasmodium falciparum isolates from Mozambique. *Malar J* 2017;16(1):416. doi: 10.1186/s12936-017-2061-z [published Online First: 2017/10/19]
7. Gamboa D, Ho MF, Bendezu J, et al. A large proportion of P. falciparum isolates in the Amazon region of Peru lack pfhrp2 and pfhrp3: implications for malaria rapid diagnostic tests. *PLoS One* 2010;5(1):e8091. doi: 10.1371/journal.pone.0008091
8. Agaba BB, Yeka A, Nsobya S, et al. Systematic review of the status of pfhrp2 and pfhrp3 gene deletion, approaches and methods used for its estimation and reporting in Plasmodium falciparum populations in Africa: review of published studies 2010-2019. *Malaria journal* 2019;18(1):355. doi: 10.1186/s12936-019-2987-4 [published Online First: 2019/11/07]
9. Mobegi VA, Duffy CW, Amambua-Ngwa A, et al. Genome-wide analysis of selection on the malaria parasite Plasmodium falciparum in West African populations of differing infection endemicity. *Mol Biol Evol* 2014;31(6):1490-9. doi: 10.1093/molbev/msu106 [published Online First: 2014/03/20]
10. Simam J, Rono M, Ngoi J, et al. Gene copy number variation in natural populations of Plasmodium falciparum in Eastern Africa. *BMC Genomics* 2018;19(1):372. doi: 10.1186/s12864-018-4689-7 [published Online First: 2018/05/23]
11. Schaffner SF, Taylor AR, Wong W, et al. hmmIBD: software to infer pairwise identity by descent between haploid genotypes. *Malar J* 2018;17(1):196. doi: 10.1186/s12936-018-2349-7 [published Online First: 2018/05/17]
12. Rice BL, Golden CD, Anjaranirina EJ, et al. Genetic evidence that the Makira region in northeastern Madagascar is a hotspot of malaria transmission. *Malar J* 2016;15(1):596. doi: 10.1186/s12936-016-1644-4 [published Online First: 2016/12/22]
13. Spanakos G, Snounou G, Pervanidou D, et al. Genetic Spatiotemporal Anatomy of Plasmodium vivax Malaria Episodes in Greece, 2009-2013. *Emerg Infect Dis* 2018;24(3):541-48. doi: 10.3201/eid2403.170605 [published Online First: 2018/02/21]
14. Chang HH, Wesolowski A, Sinha I, et al. Mapping imported malaria in Bangladesh using parasite genetic and human mobility data. *Elife* 2019;8 doi: 10.7554/eLife.43481 [published Online First: 2019/04/03]
15. Chenet SM, Silva-Flannery L, Lucchi NW, et al. Molecular Characterization of a Cluster of Imported Malaria Cases in Puerto Rico. *Am J Trop Med Hyg* 2017;97(3):758-60. doi: 10.4269/ajtmh.16-0837 [published Online First: 2017/07/28]
16. Patel JC, Taylor SM, Juliao PC, et al. Genetic Evidence of Importation of Drug-Resistant Plasmodium falciparum to Guatemala from the Democratic Republic of the Congo. *Emerg Infect Dis* 2014;20(6):932-40. doi: 10.3201/eid2006.131204 [published Online First: 2014/05/27]
17. Daniels RF, Schaffner SF, Bennett A, et al. Evidence for Reduced Malaria Parasite Population after Application of Population-Level Antimalarial Drug Strategies in Southern Province, Zambia. *Am J Trop Med Hyg* 2020 doi: 10.4269/ajtmh.19-0666 [published Online First: 2020/07/04]
18. Daniels RF, Schaffner SF, Wenger EA, et al. Modeling malaria genomics reveals transmission decline and rebound in Senegal. *Proc Natl Acad Sci U S A* 2015;112(22):7067-72. doi: 10.1073/pnas.1505691112 [published Online First: 2015/05/06]
19. Daniels R, Chang HH, Sene PD, et al. Genetic surveillance detects both clonal and epidemic transmission of malaria following enhanced intervention in Senegal. *PLoS One* 2013;8(4):e60780. doi: 10.1371/journal.pone.0060780 [published Online First: 2013/04/18]
20. Nkhoma SC, Nair S, Al-Saai S, et al. Population genetic correlates of declining transmission in a human pathogen. *Mol Ecol* 2013;22(2):273-85. doi: 10.1111/mec.12099 [published Online First: 2012/11/06]
21. Sisya TJ, Kamn'gona RM, Vareta JA, et al. Subtle changes in Plasmodium falciparum infection complexity following enhanced intervention in Malawi. *Acta Trop* 2015;142:108-14. doi: 10.1016/j.actatropica.2014.11.008 [published Online First: 2014/12/03]

For peer review only - http://bmjopen.bmj.com/site/about/guidelines.xhtml

22. Daniels R, Volkman SK, Milner DA, et al. A general SNP-based molecular barcode for Plasmodium falciparum identification and tracking. *Malar J* 2008;7:223. doi: 10.1186/1475-2875-7-223 [published Online First: 2008/10/31]
23. Chang HH, Worby CJ, Yeka A, et al. THE REAL McCOIL: A method for the concurrent estimation of the complexity of infection and SNP allele frequency for malaria parasites. *PLoS Comput Biol* 2017;13(1):e1005348. doi: 10.1371/journal.pcbi.1005348 [published Online First: 2017/01/27]
24. Mayor A, Menendez C, Walker PGT. Targeting Pregnant Women for Malaria Surveillance. *Trends Parasitol* 2019 doi: 10.1016/j.pt.2019.07.005 [published Online First: 2019/08/10]
25. Kwesigabo G, Killewo JZ, Urassa W, et al. Monitoring of HIV-1 infection prevalence and trends in the general population using pregnant women as a sentinel population: 9 years experience from the Kagera region of Tanzania. *J Acquir Immune Defic Syndr* 2000;23(5):410-7.
26. Korenromp EL, Mahiane SG, Nagelkerke N, et al. Syphilis prevalence trends in adult women in 132 countries - estimations using the Spectrum Sexually Transmitted Infections model. *Sci Rep* 2018;8(1):11503. doi: 10.1038/s41598-018-29805-9 [published Online First: 2018/08/02]
27. Galatas B, Bassat Q, Mayor A. Malaria Parasites in the Asymptomatic: Looking for the Hay in the Haystack. *Trends Parasitol* 2016;32(4):296-308. doi: 10.1016/j.pt.2015.11.015
28. Hellewell J, Walker P, Ghani A, et al. Using ante-natal clinic prevalence data to monitor temporal changes in malaria incidence in a humanitarian setting in the Democratic Republic of Congo. *Malar J* 2018;17(1):312. doi: 10.1186/s12936-018-2460-9 [published Online First: 2018/08/31]
29. Mayor A, Bardaji A, Macete E, et al. Changing Trends in P. falciparum Burden, Immunity, and Disease in Pregnancy. *N Engl J Med* 2015;373(17):1607-17. doi: 10.1056/NEJMoa1406459 [published Online First: 2015/10/22]
30. Kitojo C, Gutman JR, Chacky F, et al. Estimating malaria burden among pregnant women using data from antenatal care centres in Tanzania: a population-based study. *Lancet Glob Health* 2019;7(12):e1695-e705. doi: 10.1016/S2214-109X(19)30405-X [published Online First: 2019/11/12]
31. van Eijk A, Hill J, Snow R, et al. The relationship between the prevalence of malaria in pregnant women and the prevalence of malaria in children and non-pregnant women in Sub-Saharan Africa. *American Society of Tropical Medicine and Hygiene Meeting* 2014;91(Suppl 5):287.
32. Willilo RA, Molteni F, Mandike R, et al. Pregnant women and infants as sentinel populations to monitor prevalence of malaria: results of pilot study in Lake Zone of Tanzania. *Malar J* 2016;15(1):392. doi: 10.1186/s12936-016-1441-0
33. World Health Organization. World Malaria Report 2021. Geneva 2021. [Available from: https://www.who.int/teams/global-malaria-programme/reports/world-malaria-report-2021].
34. World Health Organization. Surveillance template protocol for pfhrp2/pfhrp3 gene deletions. Geneva 2020. [Available from: https://apps.who.int/iris/bitstream/handle/10665/331196/9789240002036-eng.pdf].
35. Chidimatembue A, Svigel SS, Mayor A, et al. Molecular surveillance for polymorphisms associated with artemisinin-based combination therapy resistance in Plasmodium falciparum isolates collected in Mozambique, 2018. *Malar J* 2021;20(1):398. doi: 10.1186/s12936-021-03930-9 [published Online First: 20211012]
36. Nhama A, Nhamussua L, Macete E, et al. In vivo efficacy and safety of artemether-lumefantrine and amodiaquine-artesunate for uncomplicated Plasmodium falciparum malaria in Mozambique, 2018. *Malar J* 2021;20(1):390. doi: 10.1186/s12936-021-03922-9 [published Online First: 20211002]
37. Ariey F, Witkowski B, Amaratunga C, et al. A molecular marker of artemisinin-resistant Plasmodium falciparum malaria. *Nature* 2014;505(7481):50-5. doi: 10.1038/nature12876 [published Online First: 20131218]
38. Venkatesan M, Alifrangis M, Roper C, et al. Monitoring antifolate resistance in intermittent preventive therapy for malaria. *Trends Parasitol* 2013;29(10):497-504. doi: 10.1016/j.pt.2013.07.008 [published Online First: 20130812]
39. Fidock DA, Nomura T, Talley AK, et al. Mutations in the P. falciparum digestive vacuole transmembrane protein PfCRT and evidence for their role in chloroquine resistance. *Mol Cell* 2000;6(4):861-71. doi: 10.1016/s1097-2765(05)00077-8
40. Tessema SK, Hathaway NJ, Teyssier NB, et al. Sensitive, highly multiplexed sequencing of microhaplotypes from the Plasmodium falciparum heterozygome. *J Infect Dis* 2020 doi: 10.1093/infdis/jiaa527 [published Online First: 2020/08/26]
41. Harris PA, Taylor R, Thielke R, et al. Research electronic data capture (REDCap)--a metadata-driven methodology and workflow process for providing translational research informatics support. *J Biomed Inform* 2009;42(2):377-81. doi: 10.1016/j.jbi.2008.08.010
42. Consortium M. Strengthening malaria surveillance for data-driven decision making in Mozambique 2019 [Available from: https://www.malariaconsortium.org/resources/publications/1255/strengthening-malaria-surveillance-for-data-driven-decision-making-in-mozambique].

For peer review only

43. World Health Organization. Master protocol for surveillance of pfhrp2/3 deletions and biobanking to support future research. Geneva: WHO; 2020 [Available from: https://apps.who.int/iris/bitstream/handle/10665/331197/9789240002050-eng.pdf?ua=1].
44. Ruiz-Arenas C, Caceres A, Lopez-Sanchez M, et al. scoreInvHap: Inversion genotyping for genome-wide association studies. *PLoS Genet* 2019;15(7):e1008203. doi: 10.1371/journal.pgen.1008203 [published Online First: 2019/07/04]
45. Eckhoff P. Mathematical models of within-host and transmission dynamics to determine effects of malaria interventions in a variety of transmission settings. *Am J Trop Med Hyg* 2013;88(5):817-27. doi: 10.4269/ajtmh.12-0007 [published Online First: 2013/04/17]
46. Amimo F, Lambert B, Magit A, et al. Plasmodium falciparum resistance to sulfadoxine-pyrimethamine in Africa: a systematic analysis of national trends. *BMJ Glob Health* 2020;5(11) doi: 10.1136/bmjgh-2020-003217
47. Some AF, Zongo I, Compaore YD, et al. Selection of drug resistance-mediating Plasmodium falciparum genetic polymorphisms by seasonal malaria chemoprevention in Burkina Faso. *Antimicrob Agents Chemother* 2014;58(7):3660-5. doi: 10.1128/AAC.02406-14 [published Online First: 2014/04/16]
48. Neafsey DE, Volkman SK. Malaria Genomics in the Era of Eradication. *Cold Spring Harb Perspect Med* 2017;7(8) doi: 10.1101/cshperspect.a025544 [published Online First: 2017/04/09]
49. Wesolowski A, Taylor AR, Chang HH, et al. Mapping malaria by combining parasite genomic and epidemiologic data. *BMC Med* 2018;16(1):190. doi: 10.1186/s12916-018-1181-9 [published Online First: 20181018]
50. Dudas G, Carvalho LM, Bedford T, et al. Virus genomes reveal factors that spread and sustained the Ebola epidemic. *Nature* 2017;544(7650):309-15. doi: 10.1038/nature22040 [published Online First: 20170412]
51. Lemey P, Rambaut A, Bedford T, et al. Unifying viral genetics and human transportation data to predict the global transmission dynamics of human influenza H3N2. *PLoS Pathog* 2014;10(2):e1003932. doi: 10.1371/journal.ppat.1003932 [published Online First: 20140220]
52. Saravanan KA, Panigrahi M, Kumar H, et al. Role of genomics in combating COVID-19 pandemic. *Gene* 2022;823:146387. doi: 10.1016/j.gene.2022.146387 [published Online First: 20220304]
53. Volkman SK, Neafsey DE, Schaffner SF, et al. Harnessing genomics and genome biology to understand malaria biology. *Nat Rev Genet* 2012;13(5):315-28. doi: 10.1038/nrg3187 [published Online First: 2012/04/13]
54. Galinsky K, Valim C, Salmier A, et al. COIL: a methodology for evaluating malarial complexity of infection using likelihood from single nucleotide polymorphism data. *Malar J* 2015;14:4. doi: 10.1186/1475-2875-14-4 [published Online First: 2015/01/21]
55. Kateera F, Nsobya SL, Tukwasibwe S, et al. Malaria case clinical profiles and Plasmodium falciparum parasite genetic diversity: a cross sectional survey at two sites of different malaria transmission intensities in Rwanda. *Malar J* 2016;15:237. doi: 10.1186/s12936-016-1287-5 [published Online First: 2016/04/27]
56. Mohd Abd Razak MR, Sastu UR, Norahmad NA, et al. Genetic Diversity of Plasmodium falciparum Populations in Malaria Declining Areas of Sabah, East Malaysia. *PLoS One* 2016;11(3):e0152415. doi: 10.1371/journal.pone.0152415 [published Online First: 2016/03/31]
57. Nabet C, Doumbo S, Jeddi F, et al. Genetic diversity of Plasmodium falciparum in human malaria cases in Mali. *Malar J* 2016;15:353. doi: 10.1186/s12936-016-1397-0 [published Online First: 2016/07/13]
58. Vafa M, Troye-Blomberg M, Anchang J, et al. Multiplicity of Plasmodium falciparum infection in asymptomatic children in Senegal: relation to transmission, age and erythrocyte variants. *Malar J* 2008;7:17. doi: 10.1186/1475-2875-7-17 [published Online First: 2008/01/25]
59. Chang HH, Park DJ, Galinsky KJ, et al. Genomic sequencing of Plasmodium falciparum malaria parasites from Senegal reveals the demographic history of the population. *Mol Biol Evol* 2012;29(11):3427-39. doi: 10.1093/molbev/mss161 [published Online First: 2012/06/27]
60. Niang M, Thiam LG, Loucoubar C, et al. Spatio-temporal analysis of the genetic diversity and complexity of Plasmodium falciparum infections in Kedougou, southeastern Senegal. *Parasit Vectors* 2017;10(1):33. doi: 10.1186/s13071-017-1976-0 [published Online First: 2017/01/21]
61. Volkman SK, Ndiaye D, Diakite M, et al. Application of genomics to field investigations of malaria by the international centers of excellence for malaria research. *Acta Trop* 2012;121(3):324-32. doi: 10.1016/j.actatropica.2011.12.002 [published Online First: 2011/12/21]
62. Zhu SJ, Almagro-Garcia J, McVean G. Deconvolution of multiple infections in Plasmodium falciparum from high throughput sequencing data. *Bioinformatics* 2018;34(1):9-15. doi: 10.1093/bioinformatics/btx530 [published Online First: 2017/09/30]
63. Cerqueira GC, Cheeseman IH, Schaffner SF, et al. Longitudinal genomic surveillance of Plasmodium falciparum malaria parasites reveals complex genomic architecture of emerging artemisinin resistance. *Genome Biol* 2017;18(1):78. doi: 10.1186/s13059-017-1204-4 [published Online First: 2017/04/30]

For peer review only - http://bmjopen.bmj.com/site/about/guidelines.xhtml

64. Baetscher DS, Clemento AJ, Ng TC, et al. Microhaplotypes provide increased power from short-read DNA sequences for relationship inference. *Mol Ecol Resour* 2018;18(2):296-305. doi: 10.1111/1755-0998.12737 [published Online First: 2017/11/17]
65. Kidd KK, Pakstis AJ, Speed WC, et al. Current sequencing technology makes microhaplotypes a powerful new type of genetic marker for forensics. *Forensic Sci Int Genet* 2014;12:215-24. doi: 10.1016/j.fsigen.2014.06.014 [published Online First: 2014/07/20]
66. Bennett L, Oldoni F, Long K, et al. Mixture deconvolution by massively parallel sequencing of microhaplotypes. *Int J Legal Med* 2019;133(3):719-29. doi: 10.1007/s00414-019-02010-7 [published Online First: 2019/02/14]
67. Kidd KK, Speed WC. Criteria for selecting microhaplotypes: mixture detection and deconvolution. *Investig Genet* 2015;6:1. doi: 10.1186/s13323-014-0018-3 [published Online First: 2015/03/10]
68. Henden L, Lee S, Mueller I, et al. Identity-by-descent analyses for measuring population dynamics and selection in recombining pathogens. *PLoS Genet* 2018;14(5):e1007279. doi: 10.1371/journal.pgen.1007279 [published Online First: 2018/05/24]
69. Lawson DJ, Hellenthal G, Myers S, et al. Inference of population structure using dense haplotype data. *PLoS Genet* 2012;8(1):e1002453. doi: 10.1371/journal.pgen.1002453 [published Online First: 2012/02/01]
70. Libbrecht MW, Noble WS. Machine learning applications in genetics and genomics. *Nat Rev Genet* 2015;16(6):321-32. doi: 10.1038/nrg3920 [published Online First: 2015/05/08]
71. Moonasar D, Maharaj R, Kunene S, et al. Towards malaria elimination in the MOSASWA (Mozambique, South Africa and Swaziland) region. *Malar J* 2016;15(1):419. doi: 10.1186/s12936-016-1470-8 [published Online First: 2016/08/20]
72. Lover AA, Harvard KE, Lindawson AE, et al. Regional initiatives for malaria elimination: Building and maintaining partnerships. *PLoS Med* 2017;14(10):e1002401. doi: 10.1371/journal.pmed.1002401 [published Online First: 20171005]
73. de Vries J, Munung SN, Matimba A, et al. Regulation of genomic and biobanking research in Africa: a content analysis of ethics guidelines, policies and procedures from 22 African countries. *BMC Med Ethics* 2017;18(1):8. doi: 10.1186/s12910-016-0165-6 [published Online First: 20170202]

For peer review only

Figure legends

Figure 1. Malaria genomic use cases and National Malaria Control Program (NMCP) decisions.

The letter on the left (A-D) expresses the level of action described in the WHO Technical consultation on the role of parasite and anopheline genetics in malaria surveillance. A: Immediate action; B: Medium-term action; C: Long-term action. Arrows in color at the right express the research required for action in the medium and long-term (grey: not essential for action; green: immediate evidence; yellow: medium-term evidence). Abbreviations: ANC, antenatal care clinics; IPT, intermittent preventive treatment; MDA, mass drug administration; rfMDA, reactive focal MDA; SMC, seasonal malaria chemoprevention.

Figure 2. Low and medium-to-high transmission study districts targeted in the protocol.

Figure 3. Modelling approaches for malaria genomics.
Overview of the components of a joint malaria epidemiology-genetic model, that builds on the capabilities of two models previously developed at the Institute of Disease Modelling (a malaria genetic model calibrated to a longitudinal genetic study in Senegal and a disease transmission model calibrated with the Magude data).

For peer review only

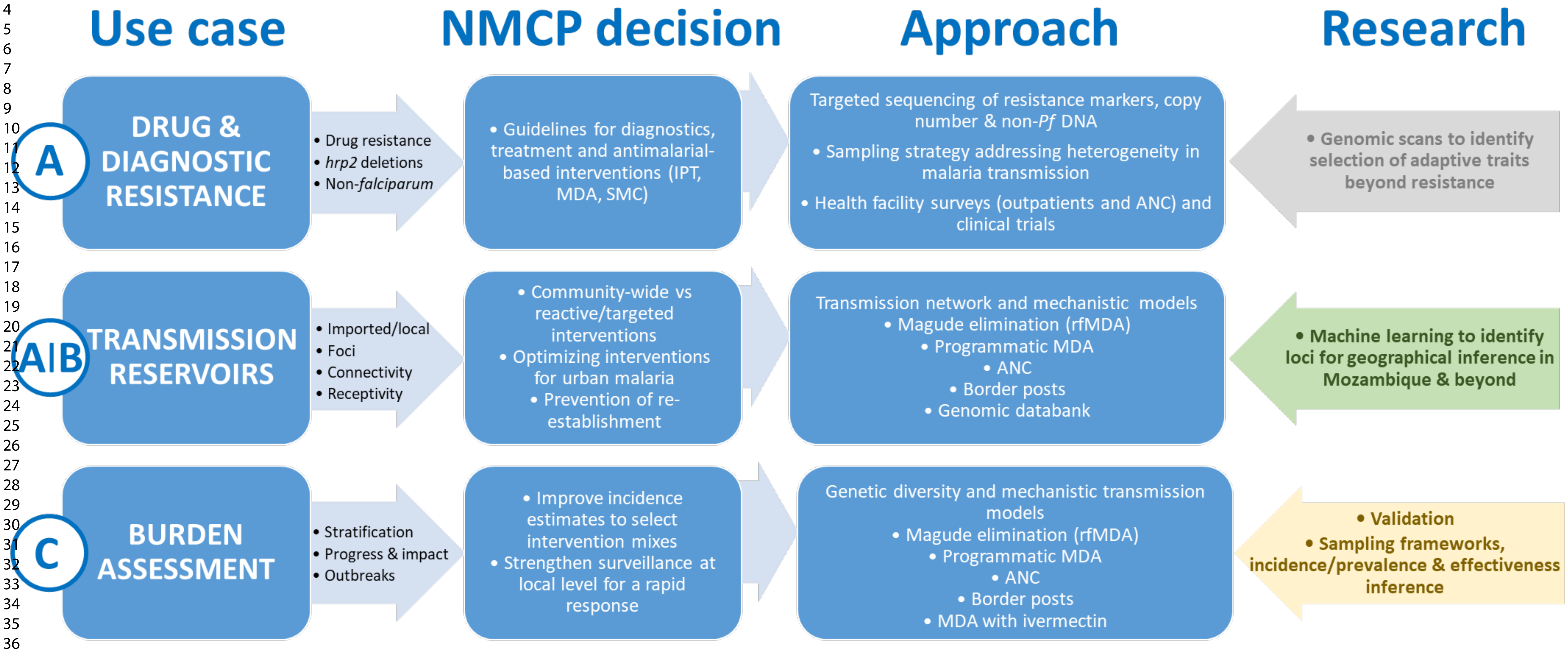
Use case
NMCP decision
Approach
Research
A
DRUG & DIAGNOSTIC RESISTANCE
• Drug resistance
• *hrp2* deletions
• Non-*falciparum*
• Guidelines for diagnostics, treatment and antimalarial-based interventions (IPT, MDA, SMC)
Targeted sequencing of resistance markers, copy number & non-*Pf* DNA
• Sampling strategy addressing heterogeneity in malaria transmission
• Health facility surveys (outpatients and ANC) and clinical trials
• Genomic scans to identify selection of adaptive traits beyond resistance
A|B
TRANSMISSION RESERVOIRS
• Imported/local
• Foci
• Connectivity
• Receptivity
• Community-wide vs reactive/targeted interventions
• Optimizing interventions for urban malaria
• Prevention of re-establishment
Transmission network and mechanistic models
• Magude elimination (rfMDA)
• Programmatic MDA
• ANC
• Border posts
• Genomic databank
• Machine learning to identify loci for geographical inference in Mozambique & beyond
C
BURDEN ASSESSMENT
• Stratification
• Progress & impact
• Outbreaks
• Improve incidence estimates to select intervention mixes
• Strengthen surveillance at local level for a rapid response
Genetic diversity and mechanistic transmission models
• Magude elimination (rfMDA)
• Programmatic MDA
• ANC
• Border posts
• MDA with ivermectin
• Validation
• Sampling frameworks, incidence/prevalence & effectiveness inference

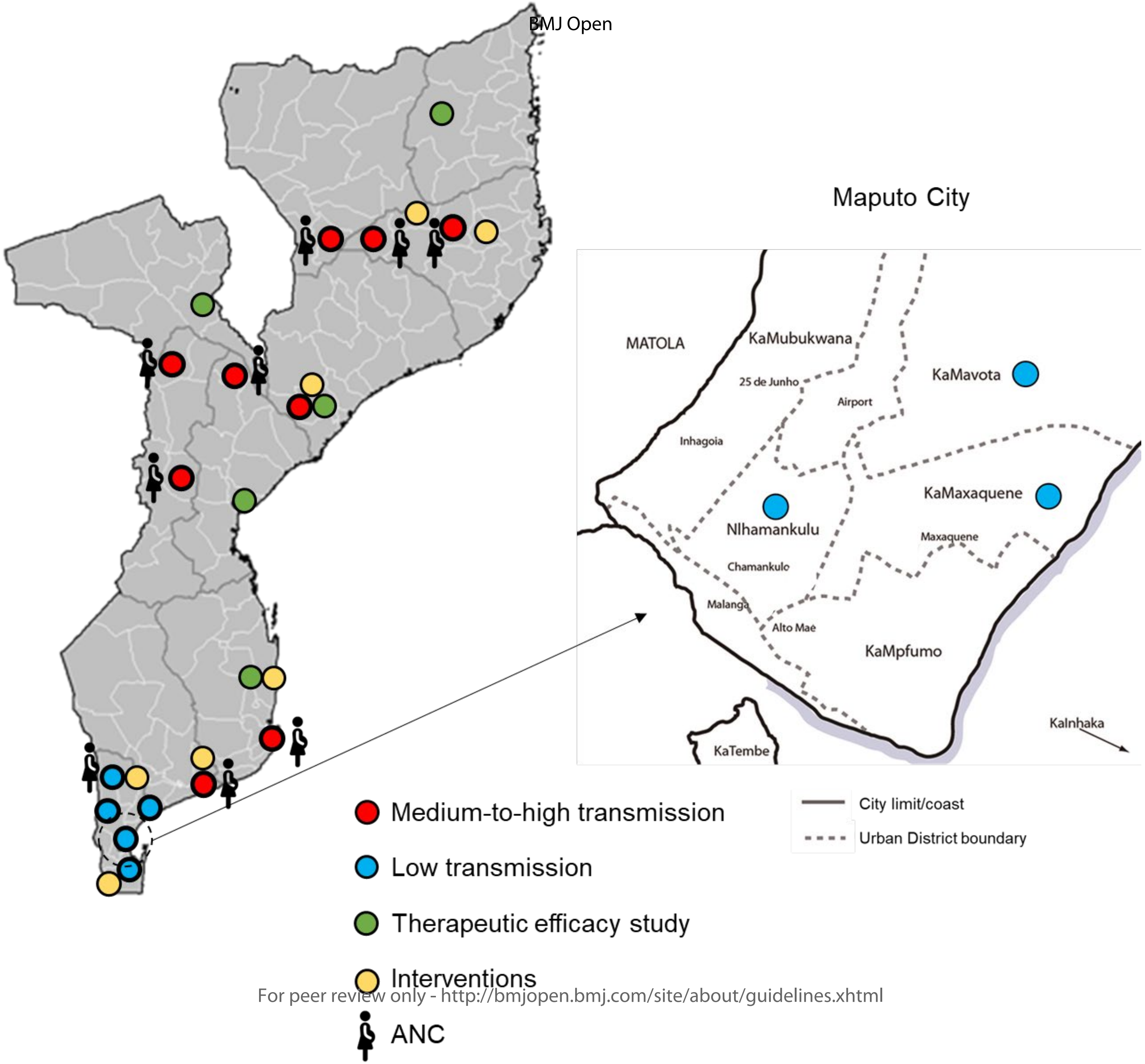
Maputo City
MATOLA
KaMubukwana
25 de Junho
Airport
KaMavota
Inhagoia
KaMaxaquene
Nlhamankulu
Maxaquene
Chamankulo
Malanga
Alto Mae
KaMpfumo
KaInhaka
KaTembe
City limit/coast
Urban District boundary
Medium-to-high transmission
Low transmission
Therapeutic efficacy study
Interventions
ANC

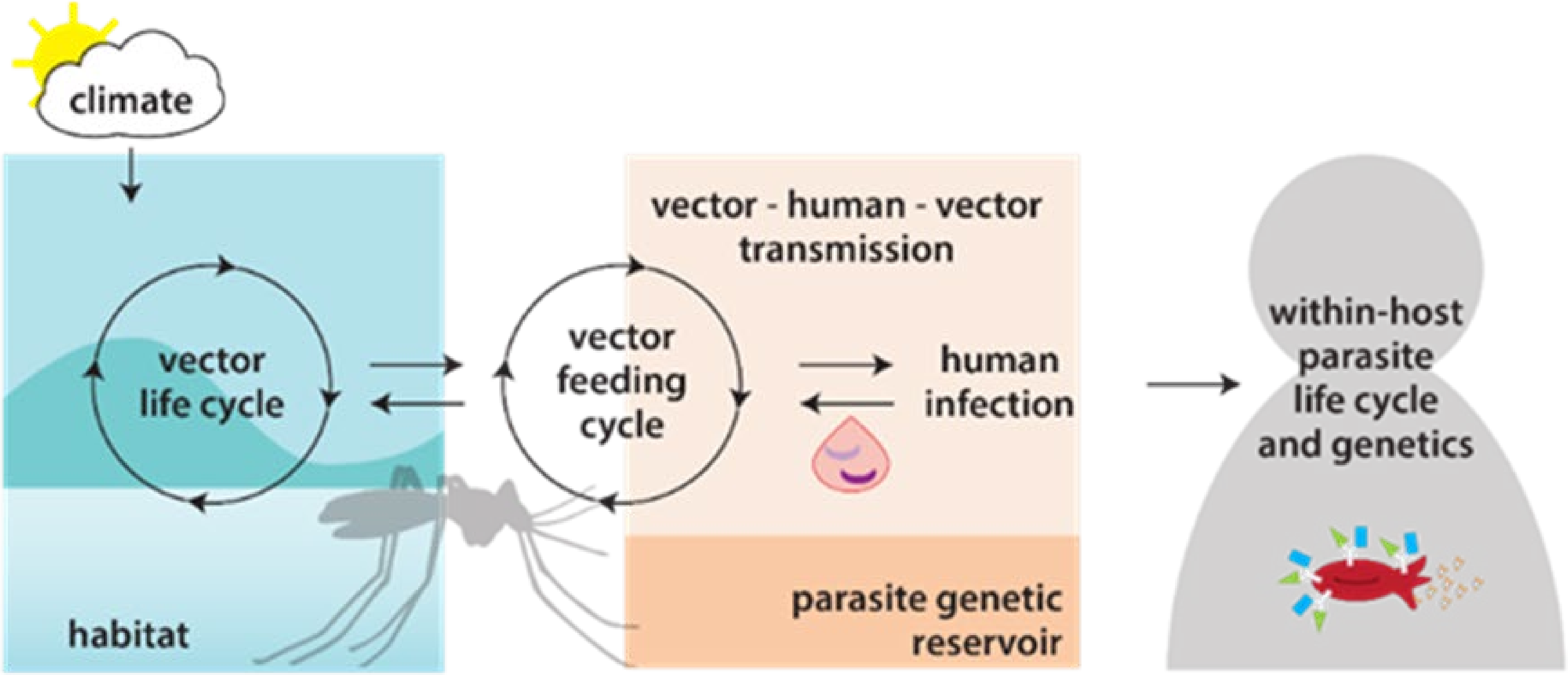
climate
vector
life cycle
habitat
vector
feeding
cycle
vector - human - vector
transmission
human
infection
parasite genetic
reservoir
within-host
parasite
life cycle
and genetics

Supplementary Table 1. Control measures for bool collection through fingerprick.

**Potential Hazards**	**Likelihood**	**Consequences**	**Control Measures**
Lancet stick injury	Rare	Possibility of infection (hep B or HIV)	Wear PPE and work slowly and carefully. The lancets for finger pricks are designed in such a way that they can only be used once, thereby minimising the possibility of cross contamination.
Incorrect blood collection procedure	Moderate	No blood drawn	Follow detailed SOPs for blood collection procedures for finger picks; dispose of all contaminated waste in the biohazard bags.
Haematoma	Rare	Bruising and painful lancet entry site	Follow correct collection procedures (SOPs), if unable to draw blood, withdraw the lancet and apply light pressure to the site. Do not attempt to withdraw blood at the same site again.
Fainting	Moderate	Subject may feel faint at the sight of blood	Lie the patient down and stay with them until they have recovered. Little sips of water and a wet towel applied to the forehead. Verbal communication throughout the procedure will reassure the subject.

For peer review only

Supplementary Table 2. Roles of each partner organization.

Organisation	Role
**National Malaria Control Programme**	Technical oversight of all research
**Malaria Consortium**	Development of the sampling protocol and ethical clearance<br>Training of data collectors<br>Field collection of blood samples and participant data<br>Transfer of samples and data to CISM<br>Creation of a surveillance dashboard<br>Transference of activities to NMCP
**Centro de Investigação em Saúde de Manhiça (CISM)**	Financial, organizational and overall coordination<br>Genetic analysis<br>Long-term storage of study samples<br>Analysis of data<br>Scientific/programmatic dissemination of results
**National Institute of Health (INS)**	Malaria Indicator and ANC Surveys in Inhambane, Gaza, Maputo and Maputo City<br>Sample analysis
**ISGlobal**	Supervision of epidemiological aspects<br>Technical support for sampling strategy, data collection and epidemiological analysis<br>Development of sequencing pipeline
**Institute for Disease Modelling**	Development and calibration of an epidemiological genomic model for Mozambique regions
**University of California San Francisco**	Development of sequencing and analytical pipelines<br>Development of transmission network model<br>Training and supervision of genetic and bioinformatics activities

For peer review only - http://bmjopen.bmj.com/site/about/guidelines.xhtml

Appendixes

Appendix 1. Information sheet and informed consent for participants over 18 years of age

Appendix 2. Information sheet and informed assent for minors aged between 12 and 17 years old

Appendix 3. Information sheet and informed consent for adult pregnant women

Appendix 4. Information sheet and informed assent for pregnant women between 12 and 18 years of age

Appendix 5. Questionnaire for Medium-high transmission area, children under 2-10 years old

Appendix 6. Questionnaire for Low transmission area, all ages

Appendix 7. Questionnaire for Pregnant women attending ANC clinic in medium-high transmission area

Appendix 8. Questionnaire for Pregnant women attending ANC clinic in low transmission area

Appendix 9. Procedures for the collection, handling and storage of dried blood samples on filter paper and rapid diagnostic tests (RDT).

Appendix 10. COVID-19 safety and research considerations

Appendix 11. Worksheet for Monitoring and Evaluation of Field Activities

For peer review only

Appendix 1. Information sheet and informed consent for participants over 18 years of age

PART I

INFORMATION SHEET AND INFORMED CONSENT FOR PARTICIPANTS OVER 18 YEARS OF AGE

Name of Affiliated Institutions

1. Manhiça Health Research Center (CISM), Manhiça, Mozambique
2. Malaria Consortium, Maputo, Mozambique
3. Barcelona Global Health Institute (ISGlobal), Barcelona, Spain
4. National Malaria Control Programme, Ministry of Health, Maputo, Mozambique
5. University of California, San Francisco, USA
6. Clinton Health Access Initiative, Boston, USA
7. Institute of Disease Modeling, Bill and Melinda Gates Foundation, Seattle, USA
8. Bill and Melinda Gates Foundation, Seattle, USA

Protocol title and version: "A prospective surveillance study to detect antimalarial drug resistance, gene deletions of diagnostic relevance and genetic diversity of *Plasmodium falciparum* in Mozambique," version number 7, 25 August 2021.

Name and affiliation of Principal Investigator(s): Baltazar Candrinho, National Malaria Control Programme, Ministry of Health, Maputo, Mozambique and Alfredo Mayor, Manhiça Health Research Center, Manhiça, Mozambique.

Study funder: Bill and Melinda Gates Foundation, USA

Introduction: The National Malaria Control Programme in partnership with Malaria Consortium and the Manhiça Health Research Center are conducting a study to analyse the genetics of malaria parasites to identify the best ways to control and/or eliminate this disease from the country.

Please read this form with care. This form provides important information about participating in this study. All the information which follows, discussed below, is to allow you to understand what the study involves and the steps that would need your collaboration, so that before becoming involved in the study, you can decide freely if you wish to participate.

You can take the time that you feel necessary to decide about your participation in this study. If you have questions about the study, or any part of this form, please ask us. If you decide to participate in this research, you will be asked to sign this form. One copy of the signed form will be provided to you for your records. If at any time you feel that you do not understand the information that is being provided, please do not hesitate to interrupt so that we can explain and clarify everything again.

After receiving your consent to participate, we will ask you some personal questions about your age, date of birth, recent illnesses, including history of fever, occupation, travel history, residence, use of insecticide treated mosquito nets or taking of antimalarial medications in the last month and then we will take a few drops of blood from your finger.

Rationale: Mozambique constitutes a main goal for the World Health Organization and partnership initiative, namely, Roll Back Malaria, to end malaria in the world. In this context, through involvement in regional malaria elimination initiatives, the use of molecular malaria surveillance data, as a complement to traditional surveillance information, can contribute to the elimination of malaria in Southern Mozambique and a reduction of the burden in the north of the country. However, there is a lack of malaria diagnostic and drug resistance data and other measures of the genetic diversity of the parasite that causes malaria in different transmission settings. Therefore, more evidence is needed to demonstrate the feasibility of using genetic data as a driver of the intensity of transmission in high transmission areas. Additionally, understanding the prevalence of diagnostic and drug resistance and genetic diversity will inform more appropriate and impactful interventions to reduce malaria morbidity and mortality in Mozambique. The integration of genetic data into routine

For peer review only - http://bmjopen.bmj.com/site/about/guidelines.xhtml

surveillance activities has the potential to increase knowledge for programmatic decision-making on the optimal combination of control and elimination measures in Mozambique.

Research objectives: Your participation in this study will help us to identify the prevalence of molecular markers of antimalarial resistance along with other genetic markers, which will inform the National Malaria Control Programme to support decision-making on the use of antimalarials and best strategies to control and eliminate malaria in the country.

Type of research/Intervention: This is prospective, operational surveillance research.

Selection of participants: You were invited to participate in this research because you are part of a group that is the focus of this study: **adults over 18 years of age** with malaria, confirmed by a rapid diagnostic test, living in this region.

Voluntary participation: It's your choice if you want to participate in this study or not. Refusing to participate or withdrawing your participation will not result in any penalty or loss of health benefits or services. You will continue to receive medical care if you choose not to participate in this study. Your decision will not change the care that you receive now or in the future. Participating in this study is your choice. If you decide to participate in this study, you can leave at any time without consequences. If you want to stop participating in the study, just let the research team know.

Procedures: We will take a few drops of blood from your finger and four drops will be placed on two small pieces of paper (filter paper), two drops on each paper. The filter papers containing four drops of blood each will be kept in the Health Unit and sent to Manhiça Health Research Center where the analysis will be done. If necessary, the filter papers may be sent to a laboratory located outside of Mozambique (specifically, the ISGlobal laboratory in Spain or the University of California, San Francisco laboratory, in the United States) for additional analysis and molecular characterisation of the malaria parasites (alleles related to antimalarial resistance as well as genetic composition and other molecular markers of relevance to malaria surveillance, both in the parasite and human host). The filter papers will be stored by the Manhiça Health Research Center for future human and parasite malaria molecular studies for a period of up to 10 years. In addition to drops of blood, all participants will also be asked about their age, date of birth, recent illnesses, including history of fever, occupation, travel history, residence, use of insecticide treated mosquito nets or antimalaria medication taking in the past 24/48 hours.

Risks, Discomfort and Inconvenience: You may feel a little pain or fear when your finger is pricked. The pain will dissipate within a few hours.

Benefits: There are no direct benefits for you to participate in this study. However, the findings generated from the study will inform the National Malaria Control Programme in decision-making about the use of antimalarials and the best strategies to control and eliminate malaria from the country.

Costs of Participation/Compensation: You will not receive any money or compensation to take part in this study.

Privacy: The data collected will be anonymous, however the data obtained in this study may be shared with collaborating partners: The National Malaria Control Programme, Malaria Consortium Mozambique, Manhiça Health Research Center, ISGlobal, Institute of Disease Modeling and the University of California, San Francisco, USA. In relation to the DNA sequences of the malaria parasite, or your personal data, these will be archived in an online database that can be shared with other scientists and researchers when the data are sent to scientific publications to report the results of this study.

Confidentiality: The information collected will be kept confidential and only the study team will have access to individuals' information. The results of the study will be published and made available so that other interested people can learn from our study, but confidential information will not be shared in any circumstance. Your data will be completely anonymised.

For peer review only - http://bmjopen.bmj.com/site/about/guidelines.xhtml

Sharing of results: Results from this research will be shared on open access platforms online, in public data repositories or directly in scientific publications, in order to facilitate further collaboration, enhance trust in the findings and goodwill among researchers. We will specifically focus on data sharing among other African countries in the region which are engaging in similar approaches to the molecular surveillance of malaria.

Whom to Contact (Investigators and Ethics Committee): in case of any of these situations:

- If your questions, concerns or complaints are not being addressed by the research team;
- If you are unable to contact the research team;
- If you would like to speak with someone who is not part of the research team;
- If you have questions about your rights as a research participant;
- If you wish to obtain information or provide information about this research; or
- If you think that the study has caused harm.

Please return to the Health Unit and speak with the workers involved in the study or contact the study focal person, assigned by Malaria Consortium Mozambique, Neide Canana on telephone number: 860450563, or you can contact her at: Malaria Consortium Mozambique, Sita Av. Lucas Elias Kumato nr. 118, Bairro da Sommerschield – Maputo City, Mozambique, or you can also contact Manhiça Health Research Center, located at: Street 12, Bairro Cambeve in Município da Manhiça Maputo Province, Mozambique, or by telephone: 21810002. In case you are not satisfied with the responses provided, you may also contact the National Bioethics for Health Committee, Ministry of Health, Mozambique on the numbers: 824066350/844693186.

Ethics Committee approval of this study: This study was approved by the Manhiça Health Research Center Institutional Bioethics Health Committee and the National Bioethics for Health Committee.

PART II
DECLARATION OF INFORMED CONSENT

Study Title: "A prospective surveillance study to detect antimalarial drug resistance, gene deletions of diagnostic relevance and genetic diversity of *Plasmodium falciparum* in Mozambique."

Declaration: I have read the information provided in this consent form, including the risks and possible benefits. All my questions about the research have been answered satisfactorily. I understand that I am free to withdraw from the study at any time without repercussions or loss of benefits to which I am entitled.

I give my consent to participate in this study.

INFORMED CONSENT
If there is any part of this consent form that you do not understand, ask the investigator before you sign.

I, ______________________ (Name of participant) give my voluntary consent to participate in the study: "A prospective surveillance study to detect antimalarial drug resistance, gene deletions of diagnostic relevance and genetic diversity of *Plasmodium falciparum* in Mozambique."

My questions have all been answered by _________________ (Name of researcher) in my own language. In case I have any other questions, I know that I can contact the study focal person assigned to Malaria Consortium and the National Bioethics for Health Committee through the contacts provided. I understand that I may withdraw my participation from the study, at any time for any reason, without any repercussions.

Do you allow your samples to be stored and used in future research? □ **Yes** □ **No**

I agree to take part in this study.

For peer review only - http://bmjopen.bmj.com/site/about/guidelines.xhtml

Signatures

Participant's fingerprint if they cannot sign	

Signature of participant — Date and time

Name of participant (in capital letters)

Signature of the person who explained consent

Name of the person who explained consent (in capital letters) — Date and time

If the participant does not know how to read, an impartial witness must also sign this form:

Signature of the impartial witness — Date and time

Name of the impartial witness (in capital letters)

For peer review only

Appendix 2. Information sheet and informed assent for minors aged between 12 and 17 years old.

PART I

INFORMATION SHEET AND INFORMED ASSENT FOR MINORS AGED BETWEEN 12 AND 17 YEARS OLD

Name of Affiliated Institutions

9. Manhiça Health Research Center (CISM), Manhiça, Mozambique
10. Malaria Consortium, Maputo, Mozambique
11. Barcelona Global Health Institute (ISGlobal), Barcelona, Spain
12. National Malaria Control Programme, Ministry of Health, Maputo, Mozambique
13. University of California, San Francisco, USA
14. Clinton Health Access Initiative, Boston, USA
15. Institute of Disease Modeling, Bill and Melinda Gates Foundation, Seattle, USA
16. Bill and Melinda Gates Foundation, Seattle, USA

Protocol title and version: "A prospective surveillance study to detect antimalarial drug resistance, gene deletions of diagnostic relevance and genetic diversity of *Plasmodium falciparum* in Mozambique," version number 7, 25 August 2021.

Name and affiliation of Principal Investigator(s): Baltazar Candrinho, National Malaria Control Programme, Ministry of Health, Maputo, Mozambique and Alfredo Mayor, Manhiça Health Research Center, Manhiça, Mozambique.

Study funder: Bill and Melinda Gates Foundation, USA

Introduction: The National Malaria Control Programme in partnership with Malaria Consortium and the Manhiça Health Research Center are conducting a study to analyse the genetics of malaria parasites to identify the best ways to control and/or eliminate this disease from the country.

Please read this form with care. This form provides important information about participating in this study. All the information which follows, discussed below, is to allow you to understand what the study involves and the steps that would need your collaboration, so that before becoming involved in the study, you can decide freely if you wish to participate.

You can take the time that you feel necessary to decide about your participation in this study. If you have questions about the study, or any part of this form, please ask us. If you decide to participate in this research, you will be asked to sign this form. One copy of the signed form will be provided to you for your records. If at any time you feel that you do not understand the information that is being provided, please do not hesitate to interrupt so that we can explain and clarify everything again.

After receiving your consent to participate, we will ask you some personal questions about your age, date of birth, recent illnesses, including history of fever, occupation, travel history, residence, use of insecticide treated mosquito nets or taking of antimalarial medications in the last month and then we will take a few drops of blood from your finger.

Rationale: Mozambique constitutes a main goal for the World Health Organization and partnership initiative, namely, Roll Back Malaria, to end malaria in the world. In this context, through involvement in regional malaria elimination initiatives, the use of molecular malaria surveillance data, as a complement to traditional surveillance information, can contribute to the elimination of malaria in Southern Mozambique and a reduction of the burden in the north of the country. However, there is a lack of malaria diagnostic and drug resistance data and other measures of the genetic diversity of the parasite that causes malaria in different transmission settings. Therefore, more evidence is needed to demonstrate the feasibility of using genetic data as a driver of the intensity of transmission in high transmission areas. Additionally, understanding the prevalence of diagnostic and drug resistance and genetic diversity will inform more appropriate and impactful interventions

For peer review only - http://bmjopen.bmj.com/site/about/guidelines.xhtml

to reduce malaria morbidity and mortality in Mozambique. The integration of genetic data into routine surveillance activities has the potential to increase knowledge for programmatic decision-making on the optimal combination of control and elimination measures in Mozambique.

Research objectives: Your participation in this study will help us to identify the prevalence of molecular markers of antimalarial resistance along with other genetic markers, which will inform the National Malaria Control Programme to support decision-making on the use of antimalarials and best strategies to control and eliminate malaria in the country.

Type of research/Intervention: This is prospective, operational surveillance research.

Selection of participants: You were invited to participate in this research because you are part of a group that is the focus of this study: **minors aged between 0 and 18 years of age** with malaria, confirmed by a rapid diagnostic test, living in this region.

Voluntary participation: It's your choice if you want to participate in this study or not. Refusing to participate or withdrawing your participation will not result in any penalty or loss of health benefits or services. You will continue to receive medical care if you choose not to participate in this study. Your decision will not change the care that you receive now or in the future. Participating in this study is your choice. If you decide to participate in this study, you can leave at any time without consequences. If you want to stop participating in the study, just let the research team know.

Procedures: We will take a few drops of blood from your finger and four drops will be placed on two small pieces of paper (filter paper), two drops on each paper. The filter papers containing four drops of blood each will be kept in the Health Unit and sent to Manhiça Health Research Center where the analysis will be done. If necessary, the filter papers may be sent to a laboratory located outside of Mozambique (specifically, the ISGlobal laboratory in Spain or the University of California, San Francisco laboratory, in the United States) for additional analysis and molecular characterisation of the malaria parasites (alleles related to antimalarial resistance as well as genetic composition and other molecular markers of relevance to malaria surveillance, both in the parasite and human host). The filter papers will be stored by the Manhiça Health Research Center for future human and parasite malaria molecular studies for a period of up to 10 years. In addition to drops of blood, all participants will also be asked about their age, date of birth, recent illnesses, including history of fever, occupation, travel history, residence, use of insecticide treated mosquito nets or antimalaria medication taking in the past 24/48 hours.

Risks, Discomfort and Inconvenience: You may feel a little pain or fear when your finger is pricked. The pain will dissipate within a few hours.

Benefits: There are no direct benefits for you to participate in this study. However, the findings generated from the study will inform the National Malaria Control Programme in decision-making about the use of antimalarials and the best strategies to control and eliminate malaria from the country.

Costs of Participation/Compensation: You will not receive any money or compensation to take part in this study.

Privacy: The data collected will be anonymous, however the data obtained in this study may be shared with collaborating partners: the National Malaria Control Programme, Malaria Consortium Mozambique, Manhiça Health Research Center, ISGlobal, Institute of Disease Modeling and the University of California, San Francisco, USA. In relation to the DNA sequences of the malaria parasite, or your personal data, these will be archived in an online database that can be shared with other scientists and researchers when the data are sent to scientific publications to report the results of this study.

Confidentiality: The information collected will be kept confidential and only the study team will have access to individuals' information. The results of the study will be published and made available so that other interested

For peer review only

people can learn from our study, but confidential information will not be shared in any circumstance. Your data will be completely anonymised.

Sharing of results: Results from this research will be shared on open access platforms online, in public data repositories or directly in scientific publications, in order to facilitate further collaboration, enhance trust in the findings and goodwill among researchers. We will specifically focus on data sharing among other African countries in the region which are engaging in similar approaches to the molecular surveillance of malaria.

Whom to Contact (Investigators and Ethics Committee): in case of any of these situations:

- If your questions, concerns or complaints are not being addressed by the research team;
- If you are unable to contact the research team;
- If you would like to speak with someone who is not part of the research team;
- If you have questions about your rights as a research participant;
- If you wish to obtain information or provide information about this research; or
- If you think that the study has caused harm.

Please return to the Health Unit and speak with the workers involved in the study or contact the study focal person, assigned by Malaria Consortium Mozambique, Neide Canana on telephone number: 860450563, or you can contact her at: Malaria Consortium Mozambique, Sita Av. Lucas Elias Kumato nr. 118, Bairro da Sommerschield – Maputo City, Mozambique, or you can also contact Manhiça Health Research Center, located at: Street 12, Bairro Cambeve in Município da Manhiça Maputo Province, Mozambique, or by telephone: 21810002. In case you are not satisfied with the responses provided, you may also contact the National Bioethics for Health Committee, Ministry of Health, Mozambique on the numbers: 824066350/844693186.

Ethics Committee approval of this study: This study was approved by the Manhiça Health Research Center Institutional Bioethics Health Committee and the National Bioethics for Health Committee.

PART II

DECLARATION OF ASSENT

Study Title: "A prospective surveillance study to detect antimalarial drug resistance, gene deletions of diagnostic relevance and genetic diversity of *Plasmodium falciparum* in Mozambique."

Declaration: I have read the information provided in this assent form, including the risks and possible benefits. All of my questions about the research have been answered satisfactorily. I understand that I am free to withdraw from the study at any time without repercussions or loss of benefits to which I am entitled.

I give my assent to participate in this study.

INFORMED ASSENT

If there is any part of this assent form that you do not understand, ask the investigator before you sign.

I, ______________________ (Name of participant) give my voluntary assent to participate in the study: "A prospective surveillance study to detect antimalarial drug resistance, gene deletions of diagnostic relevance and genetic diversity of *Plasmodium falciparum* in Mozambique."

My questions have all been answered by __________________ (Name of researcher) in my own language. In case I have any other questions, I know that I can contact the study focal person assigned to Malaria Consortium and the National Bioethics for Health Committee through the contacts provided. I understand that I may withdraw my participation from the study, at any time for any reason, without any repercussions.

For peer review only - http://bmjopen.bmj.com/site/about/guidelines.xhtml

Do you allow your samples to be stored and used in future research? □ **Yes** □ **No**

I agree to take part in this study.

Signatures

______________________ Signature of the minor	____________ Date and time	Minor's fingerprint if they cannot sign

____
Minor's name (in capital letters)

____
Signature of the person who explained assent

______________________________ ____________
Name of the person who explained assent (in capital letters) Date and time

If the minor does not know how to read, an impartial witness must also sign this form:

______________________________ ____________
Signature of the impartial witness Date and time

____
Name of the impartial witness (in capital letters)

For peer review only

PART III

INFORMATION SHEET AND INFORMED CONSENT FOR PARENTS/GUARDIANS OF MINOR PARTICIPANTS LESS THAN 18 YEARS OF AGE

Name of Affiliated Institutions

17. Manhiça Health Research Center (CISM), Manhiça, Mozambique
18. Malaria Consortium, Maputo, Mozambique
19. Barcelona Global Health Institute (ISGlobal), Barcelona, Spain
20. National Malaria Control Programme, Ministry of Health, Maputo, Mozambique
21. University of California, San Francisco, USA
22. Clinton Health Access Initiative, Boston, USA
23. Institute of Disease Modeling, Bill and Melinda Gates Foundation, Seattle, USA
24. Bill and Melinda Gates Foundation, Seattle, USA

Protocol title and version: "A prospective surveillance study to detect antimalarial drug resistance, gene deletions of diagnostic relevance and genetic diversity of *Plasmodium falciparum* in Mozambique," version number 7, 25 August 2021.

Name and affiliation of Principal Investigator(s): Baltazar Candrinho, National Malaria Control Programme, Ministry of Health, Maputo, Mozambique and Alfredo Mayor, Manhiça Health Research Center, Manhiça, Mozambique.

Study funder: Bill and Melinda Gates Foundation, USA

Introduction: The National Malaria Control Programme in partnership with Malaria Consortium and the Manhiça Health Research Center are conducting a study to analyse the genetics of malaria parasites to identify the best ways to control and/or eliminate this disease from the country.

For peer review only - http://bmjopen.bmj.com/site/about/guidelines.xhtml

Please read this form with care. This form provides important information about participating in this study. All the information which follows, discussed below, is to allow you to understand what the study involves and the steps that would need your collaboration, so that before becoming involved in the study, you can decide freely if you wish for your child to participate.

You can take the time that you feel necessary to decide about your child's participation in this study. If you have questions about the study, or any part of this form, please ask us. If you decide for your child to participate in this research, you will be asked to sign this form. One copy of the signed form will be provided to you for your records. If at any time you feel that you do not understand the information that is being provided, please do not hesitate to interrupt so that we can explain and clarify everything again.

After receiving your consent for your child to participate, we will ask them some personal questions about their age, date of birth, recent illnesses, including history of fever, occupation, travel history, residence, use of insecticide treated mosquito nets or taking of antimalarial medications in the last month and then we will take a few drops of blood from their finger.

Rationale: Mozambique constitutes a main goal for the World Health Organization and partnership initiative, namely, Roll Back Malaria, to end malaria in the world. In this context, through involvement in regional malaria elimination initiatives, the use of molecular malaria surveillance data, as a complement to traditional surveillance information, can contribute to the elimination of malaria in Southern Mozambique and a reduction of the burden in the north of the country. However, there is a lack of malaria diagnostic and drug resistance data and other measures of the genetic diversity of the parasite that causes malaria in different transmission settings. Therefore, more evidence is needed to demonstrate the feasibility of using genetic data as a driver of the intensity of transmission in high transmission areas. Additionally, understanding the prevalence of diagnostic and drug resistance and genetic diversity will inform more appropriate and impactful interventions to reduce malaria morbidity and mortality in Mozambique. The integration of genetic data into routine surveillance activities has the potential to increase knowledge for programmatic decision-making on the optimal combination of control and elimination measures in Mozambique.

Research objectives: Your child's participation in this study will help us to identify the prevalence of molecular markers of antimalarial resistance along with other genetic markers, which will inform the National Malaria Control Programme to support decision-making on the use of antimalarials and best strategies to control and eliminate malaria in the country.

Type of research/Intervention: This is prospective, operational surveillance research.

Selection of participants: Your child was invited to participate in this research because they are part of a group that is the focus of this study: **minors aged between 0 and 18 years of age** with malaria, confirmed by a rapid diagnostic test, living in this region.

Voluntary participation: It's your and your child's choice if you want them to participate in this study or not. Refusing to participate or withdrawing their participation will not result in any penalty or loss of health benefits or services. Your child will continue to receive medical care if you choose for them not to participate in this study. Your/their decision will not change the care that they receive now or in the future. Participating in this study is your/their choice. If you decide for your child to participate in this study, they can leave at any time without consequences. If they want to stop participating in the study, just let the research team know.

Procedures: We will take a few drops of blood from your child's finger and four drops will be placed on two small pieces of paper (filter paper), two drops on each paper. The filter papers containing four drops of blood each will be kept in the Health Unit and sent to Manhiça Health Research Center where the analysis will be done. If necessary, the filter papers may be sent to a laboratory located outside of Mozambique (specifically, the ISGlobal laboratory in Spain or the University of California, San Francisco laboratory, in the United States) for additional analysis and molecular characterisation of the malaria parasites (alleles related to antimalarial resistance as well as genetic composition and other molecular markers of relevance to malaria surveillance, both in the parasite and human host). The filter papers will be stored by the Manhiça Health Research Center

For peer review only - http://bmjopen.bmj.com/site/about/guidelines.xhtml

for future human and parasite malaria molecular studies for a period of up to 10 years. In addition to drops of blood, all participants will also be asked about their age, date of birth, recent illnesses, including history of fever, occupation, travel history, residence, use of insecticide treated mosquito nets or antimalaria medication taking in the past 24/48 hours.

Risks, Discomfort and Inconvenience: Your child may feel a little pain or fear when their finger is pricked. The pain will dissipate within a few hours.

Benefits: There are no direct benefits for your child to participate in this study. However, the findings generated from the study will inform the National Malaria Control Programme in decision-making about the use of antimalarials and the best strategies to control and eliminate malaria from the country.

Costs of Participation/Compensation: You will not receive any money or compensation for your child to take part in this study.

Privacy: The data collected will be anonymous, however the data obtained in this study may be shared with collaborating partners: the National Malaria Control Programme, Malaria Consortium Mozambique, Manhiça Health Research Center, ISGlobal, Institute of Disease Modeling and the University of California, San Francisco, USA. In relation to the DNA sequences of the malaria parasite, or your child's personal data, these will be archived in an online database that can be shared with other scientists and researchers when the data are sent to scientific publications to report the results of this study.

Confidentiality: The information collected will be kept confidential and only the study team will have access to individuals' information. The results of the study will be published and made available so that other interested people can learn from our study, but confidential information will not be shared in any circumstance. Your child's data will be completely anonymised.

Sharing of results: Results from this research will be shared on open access platforms online, in public data repositories or directly in scientific publications, in order to facilitate further collaboration, enhance trust in the findings and goodwill among researchers. We will specifically focus on data sharing among other African countries in the region which are engaging in similar approaches to the molecular surveillance of malaria.

Whom to Contact (Investigators and Ethics Committee): in case of any of these situations:

- If your questions, concerns or complaints are not being addressed by the research team;
- If you are unable to contact the research team;
- If you would like to speak with someone who is not part of the research team;
- If you have questions about your rights as a research participant;
- If you wish to obtain information or provide information about this research; or
- If you think that the study has caused harm.

Please return to the Health Unit and speak with the workers involved in the study or contact the study focal person, assigned by Malaria Consortium Mozambique, Neide Canana on telephone number: 860450563, or you can contact her at: Malaria Consortium Mozambique, Sita Av. Lucas Elias Kumato nr. 118, Bairro da Sommerschield – Maputo City, Mozambique, or you can also contact Manhiça Health Research Center, located at: Street 12, Bairro Cambeve in Município da Manhiça Maputo Province, Mozambique, or by telephone: 21810002. In case you are not satisfied with the responses provided, you may also contact the National Bioethics for Health Committee, Ministry of Health, Mozambique on the numbers: 824066350/844693186.

Ethics Committee approval of this study: This study was approved by the Manhiça Health Research Center Institutional Bioethics Health Committee and the National Bioethics for Health Committee.

PART IV

For peer review only - http://bmjopen.bmj.com/site/about/guidelines.xhtml

DECLARATION OF INFORMED CONSENT

Study Title: "A prospective surveillance study to detect antimalarial drug resistance, gene deletions of diagnostic relevance and genetic diversity of *Plasmodium falciparum* in Mozambique."

Declaration: I have read the information provided in this consent form, including the risks and possible benefits. All my questions about the research have been answered satisfactorily. I understand that my child is free to withdraw from the study at any time without repercussions or loss of benefits to which they are entitled.

I give my consent for my child/ward to participate in this study.

INFORMED CONSENT

If there is any part of this consent form that you do not understand, ask the investigator before you sign.

I, ____________________ (Name of father/mother/guardian) give my voluntary consent for my child or ward to participate in the study: "A prospective surveillance study to detect antimalarial drug resistance, gene deletions of diagnostic relevance and genetic diversity of *Plasmodium falciparum* in Mozambique."

My questions have all been answered by ________________ (Name of researcher) in my own language. In case I have any other questions, I know that I can contact the study focal person assigned to Malaria Consortium and the National Bioethics for Health Committee through the contacts provided. I understand that I may withdraw my child's participation from the study, at any time for any reason, without any repercussions.

Do you allow your child/ward's samples to be stored and used in future research? □ **Yes** □ **No**

I agree for my child/ward to take part in this study.

Signatures

Father/Mother/guardian fingerprint if they cannot sign

____________________________ ____________
Signature of father/mother/guardian Date and time

____
Name of father/mother/guardian (in capital letters)

____
Signature of the person who explained consent

____________________________________ ______________
Name of the person who explained consent (in capital letters) Date and time

If the father/mother/guardian does not know how to read, an impartial witness must also sign this form:

____ ___________
Signature of the impartial witness Date and time

____
Name of the impartial witness (in capital letters)

For peer review only - http://bmjopen.bmj.com/site/about/guidelines.xhtml

Appendix 3. Information sheet and informed consent for adult pregnant women.

PART I

INFORMATION SHEET AND INFORMED CONSENT FOR ADULT PREGNANT WOMEN

Name of Affiliated Institutions

25. Manhiça Health Research Center (CISM), Manhiça, Mozambique
26. Malaria Consortium, Maputo, Mozambique
27. Barcelona Global Health Institute (ISGlobal), Barcelona, Spain
28. National Malaria Control Programme, Ministry of Health, Maputo, Mozambique
29. University of California, San Francisco, USA
30. Clinton Health Access Initiative, Boston, USA
31. Institute of Disease Modeling, Bill and Melinda Gates Foundation, Seattle, USA
32. Bill and Melinda Gates Foundation, Seattle, USA

Protocol title and version: "A prospective surveillance study to detect antimalarial drug resistance, gene deletions of diagnostic relevance and genetic diversity of *Plasmodium falciparum* in Mozambique," version number 7, 25 August 2021.

Name and affiliation of Principal Investigator(s): Baltazar Candrinho, National Malaria Control Programme, Ministry of Health, Maputo, Mozambique and Alfredo Mayor, Manhiça Health Research Center, Manhiça, Mozambique.

Study funder: Bill and Melinda Gates Foundation, USA

Introduction: The National Malaria Control Programme in partnership with Malaria Consortium and the Manhiça Health Research Center are conducting a study to analyse the genetics of malaria parasites to identify the best ways to control and/or eliminate this disease from the country.

Please read this form with care. This form provides important information about participating in this study. All the information which follows, discussed below, is to allow you to understand what the study involves and the steps that would need your collaboration, so that before becoming involved in the study, you can decide freely if you wish to participate.

You can take the time that you feel necessary to decide about your participation in this study. If you have questions about the study, or any part of this form, please ask us. If you decide to participate in this research, you will be asked to sign this form. One copy of the signed form will be provided to you for your records. If at any time you feel that you do not understand the information that is being provided, please do not hesitate to interrupt so that we can explain and clarify everything again.

After receiving your consent to participate, we will ask you some personal questions about your age, date of birth, recent illnesses, including history of fever, occupation, travel history, residence, use of insecticide treated mosquito nets or taking of antimalarial medications in the last month and then we will take a few drops of blood from your finger.

Rationale: Mozambique constitutes a main goal for the World Health Organization and partnership initiative, namely, Roll Back Malaria, to end malaria in the world. In this context, through involvement in regional malaria elimination initiatives, the use of molecular malaria surveillance data, as a complement to traditional surveillance information, can contribute to the elimination of malaria in Southern Mozambique and a reduction of the burden in the north of the country. However, there is a lack of malaria diagnostic and drug resistance data and other measures of the genetic diversity of the parasite that causes malaria in different transmission settings. Therefore, more evidence is needed to demonstrate the feasibility of using genetic data as a driver of the intensity of transmission in high transmission areas. Additionally, understanding the prevalence of

For peer review only - http://bmjopen.bmj.com/site/about/guidelines.xhtml

diagnostic and drug resistance and genetic diversity will inform more appropriate and impactful interventions to reduce malaria morbidity and mortality in Mozambique. The integration of genetic data into routine surveillance activities has the potential to increase knowledge for programmatic decision-making on the optimal combination of control and elimination measures in Mozambique.

Research objectives: Your participation in this study will help us to identify the prevalence of molecular markers of antimalarial resistance along with other genetic markers, which will inform the National Malaria Control Programme to support decision-making on the use of antimalarials and best strategies to control and eliminate malaria in the country.

Type of research/Intervention: This is prospective, operational surveillance research.

Selection of participants: You were invited to participate in this research because you are part of a group that is the focus of this study: **adult pregnant women** with malaria, confirmed by a rapid diagnostic test, living in this region.

Voluntary participation: It's your choice if you want to participate in this study or not. Refusing to participate or withdrawing your participation will not result in any penalty or loss of health benefits or services. You will continue to receive medical care if you choose not to participate in this study. Your decision will not change the care that you receive now or in the future. Participating in this study is your choice. If you decide to participate in this study, you can leave at any time without consequences. If you want to stop participating in the study, just let the research team know.

Procedures: We will take a few drops of blood from your finger and four drops will be placed on two small pieces of paper (filter paper), two drops on each paper. The filter papers containing four drops of blood each will be kept in the Health Unit and sent to Manhiça Health Research Center where the analysis will be done. If necessary, the filter papers may be sent to a laboratory located outside of Mozambique (specifically, the ISGlobal laboratory in Spain or the University of California, San Francisco laboratory, in the United States) for additional analysis and molecular characterisation of the malaria parasites (alleles related to antimalarial resistance as well as genetic composition and other molecular markers of relevance to malaria surveillance, both in the parasite and human host). The filter papers will be stored by the Manhiça Health Research Center for future human and parasite malaria molecular studies for a period of up to 10 years. In addition to drops of blood, all participants will also be asked about their age, date of birth, recent illnesses, including history of fever, occupation, travel history, residence, use of insecticide treated mosquito nets or antimalaria medication taking in the past 24/48 hours.

Risks, Discomfort and Inconvenience: You may feel a little pain or fear when your finger is pricked. The pain will dissipate within a few hours.

Benefits: There are no direct benefits for you to participate in this study. However, the findings generated from the study will inform the National Malaria Control Programme in decision-making about the use of antimalarials and the best strategies to control and eliminate malaria from the country.

Costs of Participation/Compensation: You will not receive any money or compensation to take part in this study.

Privacy: The data collected will be anonymous, however the data obtained in this study may be shared with collaborating partners: the National Malaria Control Programme, Malaria Consortium Mozambique, Manhiça Health Research Center, ISGlobal, Institute of Disease Modeling and the University of California, San Francisco, USA. In relation to the DNA sequences of the malaria parasite, or your personal data, these will be archived in an online database that can be shared with other scientists and researchers when the data are sent to scientific publications to report the results of this study.

Confidentiality: The information collected will be kept confidential and only the study team will have access to individuals' information. The results of the study will be published and made available so that other interested

For peer review only - http://bmjopen.bmj.com/site/about/guidelines.xhtml

people can learn from our study, but confidential information will not be shared in any circumstance. Your data will be completely anonymised.

Sharing of results: Results from this research will be shared on open access platforms online, in public data repositories or directly in scientific publications, in order to facilitate further collaboration, enhance trust in the findings and goodwill among researchers. We will specifically focus on data sharing among other African countries in the region which are engaging in similar approaches to the molecular surveillance of malaria.

Whom to Contact (Investigators and Ethics Committee): in case of any of these situations:

- If your questions, concerns or complaints are not being addressed by the research team;
- If you are unable to contact the research team;
- If you would like to speak with someone who is not part of the research team;
- If you have questions about your rights as a research participant;
- If you wish to obtain information or provide information about this research; or
- If you think that the study has caused harm.

Please return to the Health Unit and speak with the workers involved in the study or contact the study focal person, assigned by Malaria Consortium Mozambique, Neide Canana on telephone number: 860450563, or you can contact her at: Malaria Consortium Mozambique, Sita Av. Lucas Elias Kumato nr. 118, Bairro da Sommerschield – Maputo City, Mozambique, or you can also contact Manhiça Health Research Center, located at: Street 12, Bairro Cambeve in Município da Manhiça Maputo Province, Mozambique, or by telephone: 21810002. In case you are not satisfied with the responses provided, you may also contact the National Bioethics for Health Committee, Ministry of Health, Mozambique on the numbers: 824066350/844693186.

Ethics Committee approval of this study: This study was approved by the Manhiça Health Research Center Institutional Bioethics Health Committee and the National Bioethics for Health Committee.

PART II

DECLARATION OF INFORMED CONSENT

Study Title: “A prospective surveillance study to detect antimalarial drug resistance, gene deletions of diagnostic relevance and genetic diversity of *Plasmodium falciparum* in Mozambique.”

Declaration: I have read the information provided in this consent form, including the risks and possible benefits. All my questions about the research have been answered satisfactorily. I understand that I am free to withdraw from the study at any time without repercussions or loss of benefits to which I am entitled.

I give my consent to participate in this study.

INFORMED CONSENT

If there is any part of this consent form that you do not understand, ask the investigator before you sign.

I, ______________________ (Name of participant) give my voluntary consent to participate in the study: “A prospective surveillance study to detect antimalarial drug resistance, gene deletions of diagnostic relevance and genetic diversity of *Plasmodium falciparum* in Mozambique.”

My questions have all been answered by __________________ (Name of researcher) in my own language. In case I have any other questions, I know that I can contact the study focal person assigned to Malaria Consortium and the National Bioethics for Health Committee through the contacts provided. I understand that I may withdraw my participation from the study, at any time for any reason, without any repercussions.

For peer review only

For peer review only - http://bmjopen.bmj.com/site/about/guidelines.xhtml

Do you allow your samples to be stored and used in future research? □ **Yes** □ **No**

I agree to take part in this study.

Signatures

Participant's fingerprint if they cannot sign

_______________________ _______________
Signature of participant Date and time

____
Name of participant (in capital letters)

____
Signature of the person who explained consent

_______________________ _______________
Name of the person who explained consent (in capital letters) Date and time

If the participant does not know how to read, an impartial witness must also sign this form:

_______________________ _______________
Signature of the impartial witness Date and time

____
Name of the impartial witness (in capital letters)

For peer review only

Appendix 4. Information sheet and informed assent for pregnant women between 12 and 18 years of age.

PART I

INFORMATION SHEET AND INFORMED ASSENT FOR PREGNANT WOMEN BETWEEN 12 AND 18 YEARS OF AGE

Name of Affiliated Institutions

33. Manhiça Health Research Center (CISM), Manhiça, Mozambique
34. Malaria Consortium, Maputo, Mozambique
35. Barcelona Global Health Institute (ISGlobal), Barcelona, Spain
36. National Malaria Control Programme, Ministry of Health, Maputo, Mozambique
37. University of California, San Francisco, USA
38. Clinton Health Access Initiative, Boston, USA
39. Institute of Disease Modeling, Bill and Melinda Gates Foundation, Seattle, USA
40. Bill and Melinda Gates Foundation, Seattle, USA

Protocol title and version: "A prospective surveillance study to detect antimalarial drug resistance, gene deletions of diagnostic relevance and genetic diversity of *Plasmodium falciparum* in Mozambique," version number 7, 25 August 2021.

Name and affiliation of Principal Investigator(s): Baltazar Candrinho, National Malaria Control Programme, Ministry of Health, Maputo, Mozambique and Alfredo Mayor, Manhiça Health Research Center, Manhiça, Mozambique.

Study funder: Bill and Melinda Gates Foundation, USA

Introduction: The National Malaria Control Programme in partnership with Malaria Consortium and the Manhiça Health Research Center are conducting a study to analyse the genetics of malaria parasites to identify the best ways to control and/or eliminate this disease from the country.

Please read this form with care. This form provides important information about participating in this study. All the information which follows, discussed below, is to allow you to understand what the study involves and the steps that would need your collaboration, so that before becoming involved in the study, you can decide freely if you wish to participate.

You can take the time that you feel necessary to decide about your participation in this study. If you have questions about the study, or any part of this form, please ask us. If you decide to participate in this research, you will be asked to sign this form. One copy of the signed form will be provided to you for your records. If at any time you feel that you do not understand the information that is being provided, please do not hesitate to interrupt so that we can explain and clarify everything again.

After receiving your consent to participate, we will ask you some personal questions about your age, date of birth, recent illnesses, including history of fever, occupation, travel history, residence, use of insecticide treated mosquito nets or taking of antimalarial medications in the last month and then we will take a few drops of blood from your finger.

Rationale: Mozambique constitutes a main goal for the World Health Organization and partnership initiative, namely, Roll Back Malaria, to end malaria in the world. In this context, through involvement in regional malaria elimination initiatives, the use of molecular malaria surveillance data, as a complement to traditional surveillance information, can contribute to the elimination of malaria in Southern Mozambique and a reduction of the burden in the north of the country. However, there is a lack of malaria diagnostic and drug resistance data and other measures of the genetic diversity of the parasite that causes malaria in different transmission settings. Therefore, more evidence is needed to demonstrate the feasibility of using genetic data as a driver of the intensity of transmission in high transmission areas. Additionally, understanding the prevalence of

For peer review only - http://bmjopen.bmj.com/site/about/guidelines.xhtml

diagnostic and drug resistance and genetic diversity will inform more appropriate and impactful interventions to reduce malaria morbidity and mortality in Mozambique. The integration of genetic data into routine surveillance activities has the potential to increase knowledge for programmatic decision-making on the optimal combination of control and elimination measures in Mozambique.

Research objectives: Your participation in this study will help us to identify the prevalence of molecular markers of antimalarial resistance along with other genetic markers, which will inform the National Malaria Control Programme to support decision-making on the use of antimalarials and best strategies to control and eliminate malaria in the country.

Type of research/Intervention: This is prospective, operational surveillance research.

Selection of participants: You were invited to participate in this research because you are part of a group that is the focus of this study: **pregnant women between 12 to 18 years of age** with malaria, confirmed by a rapid diagnostic test, living in this region.

Voluntary participation: It's your choice if you want to participate in this study or not. Refusing to participate or withdrawing your participation will not result in any penalty or loss of health benefits or services. You will continue to receive medical care if you choose not to participate in this study. Your decision will not change the care that you receive now or in the future. Participating in this study is your choice. If you decide to participate in this study, you can leave at any time without consequences. If you want to stop participating in the study, just let the research team know.

Procedures: We will take a few drops of blood from your finger and four drops will be placed on two small pieces of paper (filter paper), two drops on each paper. The filter papers containing four drops of blood each will be kept in the Health Unit and sent to Manhiça Health Research Center where the analysis will be done. If necessary, the filter papers may be sent to a laboratory located outside of Mozambique (specifically, the ISGlobal laboratory in Spain or the University of California, San Francisco laboratory, in the United States) for additional analysis and molecular characterisation of the malaria parasites (alleles related to antimalarial resistance as well as genetic composition and other molecular markers of relevance to malaria surveillance, both in the parasite and human host). The filter papers will be stored by the Manhiça Health Research Center for future human and parasite malaria molecular studies for a period of up to 10 years. In addition to drops of blood, all participants will also be asked about their age, date of birth, recent illnesses, including history of fever, occupation, travel history, residence, use of insecticide treated mosquito nets or antimalaria medication taking in the past 24/48 hours.

Risks, Discomfort and Inconvenience: You may feel a little pain or fear when your finger is pricked. The pain will dissipate within a few hours.

Benefits: There are no direct benefits for you to participate in this study. However, the findings generated from the study will inform the National Malaria Control Programme in decision-making about the use of antimalarials and the best strategies to control and eliminate malaria from the country.

Costs of Participation/Compensation: You will not receive any money or compensation to take part in this study.

Privacy: The data collected will be anonymous, however the data obtained in this study may be shared with collaborating partners: the National Malaria Control Programme, Malaria Consortium Mozambique, Manhiça Health Research Center, ISGlobal, Institute of Disease Modeling and the University of California, San Francisco, USA. In relation to the DNA sequences of the malaria parasite, or your personal data, these will be archived in an online database that can be shared with other scientists and researchers when the data are sent to scientific publications to report the results of this study.

Confidentiality: The information collected will be kept confidential and only the study team will have access to individuals' information. The results of the study will be published and made available so that other interested

For peer review only - http://bmjopen.bmj.com/site/about/guidelines.xhtml

people can learn from our study, but confidential information will not be shared in any circumstance. Your data will be completely anonymised.

Sharing of results: Results from this research will be shared on open access platforms online, in public data repositories or directly in scientific publications, in order to facilitate further collaboration, enhance trust in the findings and goodwill among researchers. We will specifically focus on data sharing among other African countries in the region which are engaging in similar approaches to the molecular surveillance of malaria.

Whom to Contact (Investigators and Ethics Committee): in case of any of these situations:

- If your questions, concerns or complaints are not being addressed by the research team;
- If you are unable to contact the research team;
- If you would like to speak with someone who is not part of the research team;
- If you have questions about your rights as a research participant;
- If you wish to obtain information or provide information about this research; or
- If you think that the study has caused harm.

Please return to the Health Unit and speak with the workers involved in the study or contact the study focal person, assigned by Malaria Consortium Mozambique, Neide Canana on telephone number: 860450563, or you can contact her at: Malaria Consortium Mozambique, Sita Av. Lucas Elias Kumato nr. 118, Bairro da Sommerschield – Maputo City, Mozambique, or you can also contact Manhiça Health Research Center, located at: Street 12, Bairro Cambeve in Município da Manhiça Maputo Province, Mozambique, or by telephone: 21810002. In case you are not satisfied with the responses provided, you may also contact the National Bioethics for Health Committee, Ministry of Health, Mozambique on the numbers: 824066350/844693186.

Ethics Committee approval of this study: This study was approved by the Manhiça Health Research Center Institutional Bioethics Health Committee and the National Bioethics for Health Committee.

PART II

DECLARATION OF ASSENT

Study Title: “A prospective surveillance study to detect antimalarial drug resistance, gene deletions of diagnostic relevance and genetic diversity of *Plasmodium falciparum* in Mozambique.”

Declaration: I have read the information provided in this assent form, including the risks and possible benefits. All my questions about the research have been answered satisfactorily. I understand that I am free to withdraw from the study at any time without repercussions or loss of benefits to which I am entitled.

I give my assent to participate in this study.

INFORMED ASSENT

If there is any part of this assent form that you do not understand, ask the investigator before you sign.

I, ______________________ (Name of participant) give my voluntary assent to participate in the study: “A prospective surveillance study to detect antimalarial drug resistance, gene deletions of diagnostic relevance and genetic diversity of *Plasmodium falciparum* in Mozambique.”

My questions have all been answered by __________________ (Name of researcher) in my own language. In case I have any other questions, I know that I can contact the study focal person assigned to Malaria Consortium and the National Bioethics for Health Committee through the contacts provided. I understand that I may withdraw my participation from the study, at any time for any reason, without any repercussions.

For peer review only - http://bmjopen.bmj.com/site/about/guidelines.xhtml

Do you allow your samples to be stored and used in future research? □ **Yes** □ **No**

I agree to take part in this study.

Signatures

Minor's fingerprint if they cannot sign

Signature of the minor ______________ Date and time ______________

Minor's name (in capital letters) ______________

Signature of the person who explained assent ______________

Name of the person who explained assent (in capital letters) ______________ Date and time ______________

If the minor does not know how to read, an impartial witness must also sign this form:

Signature of the impartial witness ______________ Date and time ______________

Name of the impartial witness (in capital letters) ______________

PART III

INFORMATION SHEET AND INFORMED CONSENT FOR PARENTS/GUARDIANS OF PREGNANT WOMEN LESS THAN 18 YEARS OF AGE

Name of Affiliated Institutions

41. Manhiça Health Research Center (CISM), Manhiça, Mozambique
42. Malaria Consortium, Maputo, Mozambique
43. Barcelona Global Health Institute (ISGlobal), Barcelona, Spain
44. National Malaria Control Programme, Ministry of Health, Maputo, Mozambique
45. University of California, San Francisco, USA
46. Clinton Health Access Initiative, Boston, USA
47. Institute of Disease Modeling, Bill and Melinda Gates Foundation, Seattle, USA
48. Bill and Melinda Gates Foundation, Seattle, USA

Protocol title and version: "A prospective surveillance study to detect antimalarial drug resistance, gene deletions of diagnostic relevance and genetic diversity of *Plasmodium falciparum* in Mozambique," version number 7, 25 August 2021.

Name and affiliation of Principal Investigator(s): Baltazar Candrinho, National Malaria Control Programme, Ministry of Health, Maputo, Mozambique and Alfredo Mayor, Manhiça Health Research Center, Manhiça, Mozambique.

Study funder: Bill and Melinda Gates Foundation, USA

Introduction: The National Malaria Control Programme in partnership with Malaria Consortium and the Manhiça Health Research Center are conducting a study to analyse the genetics of malaria parasites to identify the best ways to control and/or eliminate this disease from the country.

For peer review only - http://bmjopen.bmj.com/site/about/guidelines.xhtml

Please read this form with care. This form provides important information about participating in this study. All the information which follows, discussed below, is to allow you to understand what the study involves and the steps that would need your collaboration, so that before becoming involved in the study, you can decide freely if you wish to participate.

You can take the time that you feel necessary to decide about your participation in this study. If you have questions about the study, or any part of this form, please ask us. If you decide for your child/ward to participate in this research, you will be asked to sign this form. One copy of the signed form will be provided to you for your records. If at any time you feel that you do not understand the information that is being provided, please do not hesitate to interrupt so that we can explain and clarify everything again.

After receiving your consent for your child/ward to participate, we will ask them some personal questions about their age, date of birth, recent illnesses, including history of fever, occupation, travel history, residence, use of insecticide treated mosquito nets or taking of antimalarial medications in the last month and then we will take a few drops of blood from their finger.

Rationale: Mozambique constitutes a main goal for the World Health Organization and partnership initiative, namely, Roll Back Malaria, to end malaria in the world. In this context, through involvement in regional malaria elimination initiatives, the use of molecular malaria surveillance data, as a complement to traditional surveillance information, can contribute to the elimination of malaria in Southern Mozambique and a reduction of the burden in the north of the country. However, there is a lack of malaria diagnostic and drug resistance data and other measures of the genetic diversity of the parasite that causes malaria in different transmission settings. Therefore, more evidence is needed to demonstrate the feasibility of using genetic data as a driver of the intensity of transmission in high transmission areas. Additionally, understanding the prevalence of diagnostic and drug resistance and genetic diversity will inform more appropriate and impactful interventions to reduce malaria morbidity and mortality in Mozambique. The integration of genetic data into routine surveillance activities has the potential to increase knowledge for programmatic decision-making on the optimal combination of control and elimination measures in Mozambique.

Research objectives: Your child/ward's participation in this study will help us to identify the prevalence of molecular markers of antimalarial resistance along with other genetic markers, which will inform the National Malaria Control Programme to support decision-making on the use of antimalarials and best strategies to control and eliminate malaria in the country.

Type of research/Intervention: This is prospective, operational surveillance research.

Selection of participants: Your child/ward was invited to participate in this research because they are part of a group that is the focus of this study: **pregnant women between 12 to 18 years of age** with malaria, confirmed by a rapid diagnostic test, living in this region.

Voluntary participation: It's your choice if you want your child/ward to participate in this study or not. Refusing to participate or withdrawing their participation will not result in any penalty or loss of health benefits or services. Your child/ward will continue to receive medical care if you/they choose not to participate in this study. Your decision will not change the care that they receive now or in the future. Participating in this study is your/their choice. If you decide for them to participate in this study, they can leave at any time without consequences. If they want to stop participating in the study, just let the research team know.

Procedures: We will take a few drops of blood from your child/ward's finger and four drops will be placed on two small pieces of paper (filter paper), two drops on each paper. The filter papers containing four drops of blood each will be kept in the Health Unit and sent to Manhiça Health Research Center where the analysis will be done. If necessary, the filter papers may be sent to a laboratory located outside of Mozambique (specifically, the ISGlobal laboratory in Spain or the University of California, San Francisco laboratory, in the United States) for additional analysis and molecular characterisation of the malaria parasites (alleles related to antimalarial resistance as well as genetic composition and other molecular markers of relevance to malaria surveillance,

For peer review only - http://bmjopen.bmj.com/site/about/guidelines.xhtml

both in the parasite and human host). The filter papers will be stored by the Manhiça Health Research Center for future human and parasite malaria molecular studies for a period of up to 10 years. In addition to drops of blood, all participants will also be asked about their age, date of birth, recent illnesses, including history of fever, occupation, travel history, residence, use of insecticide treated mosquito nets or antimalaria medication taking in the past 24/48 hours.

Risks, Discomfort and Inconvenience: Your child/ward may feel a little pain or fear when their finger is pricked. The pain will dissipate within a few hours.

Benefits: There are no direct benefits for you to participate in this study. However, the findings generated from the study will inform the National Malaria Control Programme in decision-making about the use of antimalarials and the best strategies to control and eliminate malaria from the country.

Costs of Participation/Compensation: You will not receive any money or compensation for your child/ward to take part in this study.

Privacy: The data collected will be anonymous, however the data obtained in this study may be shared with collaborating partners: the National Malaria Control Programme, Malaria Consortium Mozambique, Manhiça Health Research Center, ISGlobal, Institute of Disease Modeling and the University of California, San Francisco, USA. In relation to the DNA sequences of the malaria parasite, or your child/ward's personal data, these will be archived in an online database that can be shared with other scientists and researchers when the data are sent to scientific publications to report the results of this study.

Confidentiality: The information collected will be kept confidential and only the study team will have access to individuals' information. The results of the study will be published and made available so that other interested people can learn from our study, but confidential information will not be shared in any circumstance. Your child/ward's data will be completely anonymised.

Sharing of results: Results from this research will be shared on open access platforms online, in public data repositories or directly in scientific publications, in order to facilitate further collaboration, enhance trust in the findings and goodwill among researchers. We will specifically focus on data sharing among other African countries in the region which are engaging in similar approaches to the molecular surveillance of malaria.

Whom to Contact (Investigators and Ethics Committee): in case of any of these situations:

- If your questions, concerns or complaints are not being addressed by the research team;
- If you are unable to contact the research team;
- If you would like to speak with someone who is not part of the research team;
- If you have questions about your rights as a research participant;
- If you wish to obtain information or provide information about this research; or
- If you think that the study has caused harm.

Please return to the Health Unit and speak with the workers involved in the study or contact the study focal person, assigned by Malaria Consortium Mozambique, Neide Canana on telephone number: 860450563, or you can contact her at: Malaria Consortium Mozambique, Sita Av. Lucas Elias Kumato nr. 118, Bairro da Sommerschield – Maputo City, Mozambique, or you can also contact Manhiça Health Research Center, located at: Street 12, Bairro Cambeve in Município da Manhiça Maputo Province, Mozambique, or by telephone: 21810002. In case you are not satisfied with the responses provided, you may also contact the National Bioethics for Health Committee, Ministry of Health, Mozambique on the numbers: 824066350/844693186.

Ethics Committee approval of this study: This study was approved by the Manhiça Health Research Center Institutional Bioethics Health Committee and the National Bioethics for Health Committee.

PART IV

For peer review only - http://bmjopen.bmj.com/site/about/guidelines.xhtml

DECLARATION OF INFORMED CONSENT

Study Title: "A prospective surveillance study to detect antimalarial drug resistance, gene deletions of diagnostic relevance and genetic diversity of *Plasmodium falciparum* in Mozambique."

Declaration: I have read the information provided in this consent form, including the risks and possible benefits. All my questions about the research have been answered satisfactorily. I understand that my child/ward is free to withdraw from the study at any time without repercussions or loss of benefits to which I am entitled.

I give my consent for my child/ward to participate in this study.

INFORMED CONSENT

If there is any part of this consent form that you do not understand, ask the investigator before you sign.

I, ______________________ (Name of father/mother/guardian) give my voluntary consent for my child or ward to participate in the study: "A prospective surveillance study to detect antimalarial drug resistance, gene deletions of diagnostic relevance and genetic diversity of *Plasmodium falciparum* in Mozambique."

My questions have all been answered by __________________ (Name of researcher) in my own language. In case I have any other questions, I know that I can contact the study focal person assigned to Malaria Consortium and the National Bioethics for Health Committee through the contacts provided. I understand that my child/ward may withdraw their participation from the study, at any time for any reason, without any repercussions.

Do you allow your child/ward's samples to be stored and used in future research? □ **Yes** □ **No**

I agree for my child/ward to take part in this study.

Signatures

________________________________ ______________

Signature of father/mother/guardian Date and time

Father/mother/guardian fingerprint if they cannot sign

____

Name of father/mother/guardian (in capital letters)

____

Signature of the person who explained consent

____ ________________

Name of the person who explained consent (in capital letters) Date and time

If the participant/legal representative does not know how to read, an impartial witness must also sign this form:

____ _____________

Signature of the impartial witness Date and time

____

Name of the impartial witness (in capital letters)

For peer review only - http://bmjopen.bmj.com/site/about/guidelines.xhtml

Appendix 5. Questionnaire for medium-high transmission area, children under 2-10 years old.

**Study site information**	
1. Date of sample collection (dd/mm/yy)	|__|__||__|__||__|__|
2. Province of residence	[______________]
3. District of residence	[______________]
4. Administrative post	[______________]
5. Place of residence	[______________]
6. Health Unit (name or code)	[______________]
7. Referred by APE in the community	Yes ☐ No ☐
**Inclusion criteria**	
8. Was the informed consent form signed? ***If no, end the survey.***	Yes ☐ No ☐
9. History of fever/hot body in the last 24 hours?	Yes ☐ No ☐
10. Axillary temperature at the time of the survey ***If temperature is <37.5ºC, end the survey.***	|__|__|.|__| ºC
11. Date of birth (dd/mm/yyyy)	|__|__|/|__|__|/|__|__|__|__|
11.1. Age (years)	|__|__|
12. Does the participant have severe malaria? **If yes, end the survey.**	Yes ☐ No ☐
13. Does the participant reside in the study area (district)? ***If no, end the survey.***	Yes ☐ No ☐
14. Has the child taken antimalarials in the past 14 days? (check yellow health card) ***If yes, end the survey.***	Yes ☐ No ☐
15. Was a routine RDT performed? ***If no, end the survey.***	Yes ☐ No ☐
15.1 *If yes, the result was:* ***If negative, end the survey.***	Positive ☐ Negative ☐ Inconclusive ☐
16. Was an additional RDT performed?	Yes ☐ No ☐ Not applicable ☐ **If 'not applicable', skip to question 17.**
16.1. Result of line T1 (HRP2)	Positive ☐ Negative ☐ Inconclusive ☐
16.2. Result of line T2 (LDH)	Positive ☐ Negative ☐ Inconclusive ☐
**Participant information**	
17. Sex	Male ☐ Female ☐
Study ID number *18.* *US*|__|__|-|__|__|-|__|__|__|	19. Sample ID number *Insert bar code* **Now put the sample ID number on the informed consent form.**
**Travel information**	
20. Have you travelled in the past 28 days?	Yes ☐ No ☐

For peer review only - http://bmjopen.bmj.com/site/about/guidelines.xhtml

If not, go to question 21.

20.1 When did you start your trip? (date: dd/mm) |__|__||__|__|

20.2 If yes, for how many nights? |__|__|

20.3 Where did you travel?:

Country [________________]

Province [________________]

District [________________]

20.4 During the trip, did you sleep under a mosquito net? Yes ☐ No ☐

**Information related to malaria**	
21. How many times has the child had episodes of fever in the past month?	|__|__|
22. Did the child sleep under a mosquito net last night??	Yes ☐ No ☐
22.1 *If yes, was it an insecticide treated net?*	Yes ☐ No ☐
23. Has there been indoor residual spraying in the past 6 months?	Yes ☐ No ☐
24. Has the child taken antimalarial medications in the past month?	Yes ☐ No ☐
25. Is the child taking cotrimoxazole?	Yes ☐ No ☐ Don`t know ☐
Now label the two filter papers (sample ID number)	
26. Was a blood sample collected on the filter paper?	Yes ☐ No ☐
27. If yes, state the number of papers	2 ☐ Other |__|

**Interviewer information**	
28. Interviewer number	|__|__|__|__|__|
29. Interviewer initials	|__|__|__|__|__|
30. Date of interview	(dd/mm/yyyy) |__|__|/|__|__|/|__|__|__|__|

For peer review only

Appendix 6. Questionnaire for low transmission area, all ages.

	**Study site information**	
1.	Date of sample collection (dd/mm/yy)	|__|__||__|__||__|__|
2.	Province of residence	[ ]
3.	District of residence	[ ]
4.	Administrative post	[ ]
5.	Place of residence	[ ]
6.	Health Unit (name or code)	[ ]
7.	Referred by APE in the community	Yes ☐ No ☐
	**Inclusion criteria**	
8.	Was the informed consent form signed?<br>***If no, end the survey.***	Yes ☐ No ☐
9.	History of fever/hot body in the last 24 hours?	Yes ☐ No ☐
10.	Axillary temperature at the time of the survey<br>***If temperature is <37.5ºC, end the survey.***	|__|__|.|__| ºC
11.	Date of birth (dd/mm/yyyy)<br><br>11.1. Age (years)	|__|__|/|__|__|/|__|__|__|__| <br>|__|__|
12.	Does the participant have severe malaria?<br>***If yes, end the survey.***	Yes ☐ No ☐
13.	Does the participant reside in the study area (district)?<br>***If no, end the survey.***	Yes ☐ No ☐
14.	Has the child/adult taken antimalarials in the past 14 days? (check yellow health card)<br>***If yes, end the survey.***	Yes ☐ No ☐
15.	Was a routine RDT performed?<br>***If no, end the survey.***<br>15.1 *If yes, the result was:*<br><br>***If negative, end the survey.***	Yes ☐ No ☐<br><br>Positive ☐ Negative ☐ Inconclusive ☐
16.	Was an additional RDT performed?	Yes ☐ No ☐ Not applicable ☐<br>**If 'not applicable', skip to question 17.**
	16.1. Result of line T1 (HRP2)	Positive ☐ Negative ☐ Inconclusive ☐
	16.2. Result of line T2 (LDH)	Positive ☐ Negative ☐ Inconclusive ☐
	**Participant information**	
17.	Sex	Male ☐ Female ☐
18.	Occupation	[ ]
19.	Study ID number<br><br>US|__|__|-|__|__|-|__|__|__|	**Sample ID number** *Insert bar code*<br><br>**Now put the sample ID number on the informed consent form.**

26

For peer review only - http://bmjopen.bmj.com/site/about/guidelines.xhtml

Travel information

20.	Have you travelled in the past 28 days? ***If not, go to question 21.***	Yes ☐ No ☐
20.1	When did you start your trip? (date: dd/mm)	|__|__||__|__|
20.2	If yes, for how many nights?	|__|__|
20.3	Where did you travel?: Country / Province / District	[__________] [__________] [__________]
20.4	During the trip, did you sleep under a mosquito net?	Yes ☐ No ☐

Information related to malaria

21.	How many times has the child/adult had episodes of fever in the past month?	|__|__|
22.	Did the child/adult sleep under a mosquito net last night?	Yes ☐ No ☐
22.1	*If yes, was it an insecticide treated net?*	Yes ☐ No ☐
23.	Has there been indoor residual spraying in the past 6 months?	Yes ☐ No ☐
24.	Has the child/adult taken antimalarial medications in the past month?	Yes ☐ No ☐
25.	Is the child/adult taking cotrimoxazole?	Yes ☐ No ☐ Don`t know ☐
	Now label the two filter papers (sample ID number)	
26.	Was a blood sample collected on the filter paper?	Yes ☐ No ☐
27.	If yes, state the number of papers	2 ☐ Other |__|

Interviewer information

28.	Interviewer number	|__|__|__|__|
29.	Interviewer initials	|__|__|__|__|
30.	Date of interview	(dd/mm/yyyy) |__|__|/|__|__|/|__|__|__|__|

For peer review only - http://bmjopen.bmj.com/site/about/guidelines.xhtml

Appendix 7. Questionnaire for Pregnant women attending ANC clinic in medium-high transmission area.

	**Study site information**	
1.	Date of sample collection (dd/mm/yy)	|__|__||__|__||__|__|
2.	Province of residence	[________________]
3.	District of residence	[________________]
4.	Administrative post	[________________]
5.	Place of residence	[________________]
6.	Health Unit (name or code)	[________________]
7.	Referred by APE in the community	Yes ☐ No ☐
	**Inclusion criteria**	
8.	Was the informed consent form signed?<br>***If no, end the survey.***	Yes ☐ No ☐
9.	Is the participant pregnant?<br>***If no, end the survey.***	Yes ☐ No ☐
10.	Is this your first prenatal consult?<br>***If no, end the survey.***	Yes ☐ No ☐
11.	Date of birth (dd/mm/yyyy)	|__|__|/|__|__|/|__|__|__|__|
	11.1. Age (years)<br>***If aged <12 years, end the survey.***	|__|__|
12.	Does the participant reside in the study area (district)?<br>***If no, end the survey.***	Yes ☐ No ☐
13.	Does the participant have severe malaria?<br>***If yes, end the survey.***	Yes ☐ No ☐
	**Participant information**	
14.	Occupation	[________________]
15.	Study ID number<br>**PN**|__|__|-|__|__|__|-|__|__|__|__|	Sample ID number: *Insert bar code*

Now put the sample ID number on the informed consent form.

	**Participant characteristics**	
16.	History of fever/hot body in the last 24 hours?	Yes ☐ No ☐
17.	Axillary temperature at the time of the survey	|__|__|.|__| ºC
18.	Was an HIV test performed during this visit? (*check HIV card or proof of testing)*	Yes ☐ No ☐
	18.1. *If yes, HIV test result at this visit*	Positive ☐ Negative ☐ Inconclusive ☐
	18.2. *If no, state previous HIV test result (check HIV card or proof of testing)*	Negative ☐ Positive ☐
19.	Are you receiving ART? *(check in the woman's personal health record)*	Yes ☐ No ☐
20.	Are you taking cotrimoxazole? *(check in the woman's book)*	Yes ☐ No ☐
21.	Current haemoglobin result *(Hemocue test result from today)*	|__|__|, |__| g/dL
22.	Was a malaria RDT performed?	Yes ☐ No ☐

For peer review only - http://bmjopen.bmj.com/site/about/guidelines.xhtml

23.	*If yes, the result:*	Positive ☐ Negative ☐ Inconclusive ☐
24.	How many weeks pregnant are you currently?	|__|__|
25.	Method used to determine gestational age:<br><br>a) Last menstrual period ☐<br>b) Fundal height ☐<br>c) Other (specify) [________________]	
26.	How many previous pregnancies has the participant had before this one?	|__|__|
27.	Has the participant moved from the area during the last pregnancy?	Yes ☐ No ☐
	**Travel information**	
28.	Have you travelled during this pregnancy and spent the night away from home?	Yes ☐ No ☐
	28.1 When did you start your trip? (date: dd/mm)	|__|__||__|__|
	28.2 If yes, for how many nights?	
	28.3 Where did you travel?:<br>Country<br>Province<br>District	|__|__| [________________]<br>[________________]<br>[________________]
	28.4 During the trip, did you sleep under a mosquito net?	Yes ☐ No ☐
	**Information related to malaria**	
29.	How many times have you had episodes of fever in the past month?	|__|__|
30.	Did you sleep under a mosquito net last night?	Yes ☐ No ☐
30.1	*If yes, was it an insecticide treated net?*	Yes ☐ No ☐
31.	Has there been indoor residual spraying in the past 6 months?	Yes ☐ No ☐
32.	Has the participant received intermittent preventive treatment (IPT) before this visit for this pregnancy?	Yes ☐ No ☐
33.	Has the participant taken antimalarial medications in the past month?	Yes ☐ No ☐ Don`t know ☐
	**Now label the filter paper with the sample ID number**	
34.	Was a blood sample collected on the filter paper?	Yes ☐ No ☐
35.	If yes, state the number of papers	2 ☐ Other |__|

	**Interviewer information**	
36.	Interviewer number	|__|__|__|__|__|
37.	Interviewer initials	|__|__|__|__|__|
38.	Date of interview	(dd/mm/yyyy) |__|__|/|__|__|/|__|__|__|__|

For peer review only - http://bmjopen.bmj.com/site/about/guidelines.xhtml

Appendix 8. Questionnaire for Pregnant women attending ANC clinic in low transmission area.

	**Study site information**	
1	Date of sample collection (dd/mm/yy)	I__I__II__I__II__I__I
2.	Province of residence	[ ]
3.	District of residence	[ ]
4.	Administrative post	[ ]
5.	Place of residence	[ ]
6.	Neighbourhood of residence	[ ]
7.	Mobile phone number	[ ]
8.	Health Unit (name or code)	[ ]
9.	Referred by APE in the community	Yes ☐ No ☐
	**Inclusion criteria**	
10.	Was the informed consent form signed?<br>***If no, end the survey.***	Yes ☐ No ☐
11.	Is the participant pregnant?<br>***If no, end the survey.***	Yes ☐ No ☐
12.	Is this your first prenatal consult?<br>***If no, end the survey.***	Yes ☐ No ☐
13.	Date of birth (dd/mm/yyyy)<br>I__I__I/I__I__I/I__I__I__I__I<br>11.1. Age (years)<br>I__I__I<br>***If aged <12 years, end the survey.***	
14.	Does the participant reside in the study area (district)?<br>***If no, end the survey.***	Yes ☐ No ☐
15.	Does the participant have severe malaria?<br>***If yes, end the survey.***	Yes ☐ No ☐
	**Participant information**	
16.	Participant name	[ ]
17.	Occupation	[ ]
18.	Study number<br>**PN**I__I__I-I__I__I-I__I__I__I	Sample ID number — *Insert bar code*

Now put the sample ID number on the informed consent form.

	**Participant characteristics**	
19.	History of fever/hot body in the last 24 hours?	Yes ☐ No ☐
20.	Axillary temperature at the time of the survey	I__I__I.I__I ºC
21.	Was an HIV test performed during this visit? (*check HIV card or proof of testing*)	Yes ☐ No ☐
	18.1. *If yes, HIV test result at this visit*	Positive ☐ Negative ☐ Inconclusive ☐
	18.2. *If no, state previous HIV test result (check HIV card or proof of testing)*	Negative ☐ Positive ☐

22.	Are you receiving ART? *(check in the woman's personal health record)*	Yes ☐ No ☐
23.	Are you taking cotrimoxazole? *(check in the woman's book)*	Yes ☐ No ☐
24.	Current haemoglobin result *(Hemocue test result from today)*	|__|__|, |__| g/dL
25.	Was a malaria RDT performed?	Yes ☐ No ☐
26.	*If yes, the result:*	Positive ☐ Negative ☐ Inconclusive ☐
27.	How many weeks pregnant are you currently?	|__|__|
28.	Method used to determine gestational age:<br><br>a) Last menstrual period ☐<br>b) Fundal height ☐<br>c) Other (specify) [__________________]	
29.	How many previous pregnancies has the participant had before this one?	|__|__|
30.	Has the participant moved from the area during the last pregnancy?	Yes ☐ No ☐
	**Travel information**	
31.	Have you travelled during this pregnancy and spent the night away from home?	Yes ☐ No ☐
	31.1 When did you start your trip? (date: dd/mm)	|__|__||__|__|
	31.2 If yes, for how many nights?	
	31.3 Where did you travel?:	|__|__|
	Country	[__________________]
	Province	[__________________]
	District	[__________________]
	28.4 During the trip, did you sleep under a mosquito net?	Yes ☐ No ☐
	**Information related to malaria**	
32.	How many times have you had episodes of fever in the past month?	|__|__|
33.	Did you sleep under a mosquito net last night?	Yes ☐ No ☐
33.1	*If yes, was it an insecticide treated net?*	Yes ☐ No ☐
34.	Has there been indoor residual spraying in the past 6 months?	Yes ☐ No ☐
35.	Has the participant received intermittent preventive treatment (IPT) before this visit for this pregnancy?	Yes ☐ No ☐
36.	Has the participant taken antimalarial medications in the past month?	Yes ☐ No ☐ Don`t know ☐
	**Now label the filter paper with the sample ID number**	
37.	Was a blood sample collected on the filter paper?	Yes ☐ No ☐
38.	If yes, state the number of papers	2 ☐ Other |__|
	**Interviewer information**	
39.	Interviewer number	|__|__|__|__|
40.	Interviewer initials	|__|__|__|__|
41.	Date of interview	(dd/mm/yyyy) |__|__|/|__|__|/|__|__|__|__|

For peer review only

Appendix 9. Procedures for the collection, handling and storage of dried blood samples on filter paper and rapid diagnostic tests.

1 OBJECTIVES

To describe the correct collection, handling and storage procedures for dried blood samples on filter paper and rapid diagnostic tests (RDTs).

2 DEFINITIONS

- Filter paper: semipermeable paper used as a laboratory tool to collect and store blood samples for further molecular analysis. The filter paper code that will be used is Whatman Grade CF12 cut to 76x30mm (equal to the size of a microscope slide).
- **Rapid diagnostic test (RDT):** Lateral flow immunochromatographic tests. The RDTs for human malaria detect parasite specific antigens which are present in the blood of infected people. The most commonly used antigens are *Plasmodium falciparum Histidine-rich Protein 2* (PfHRP-2) and *Plasmodium Lactate Dehydrogenase* (pLDH).

3 APPLICABLE FOR

- All personnel responsible for the collection, handling and storage of dried blood samples on filter paper rand RDTs within the scope of malaria studies at CISM.

4 RESPONSIBILITIES

- **Investigators:** to guarantee that the SOPs are up to date and that technical personnel are properly trained and strictly follow the procedures described therein.
- **All technical personnel:** whether researcher, laboratory technicians, phlebotomists, physicians, or others who are engaged in field, clinical or laboratory activities involving filter papers or RDTs; all must know and strictly follow the content of these SOPs.

5 RELATED SOPS

- **POP_LB_012_PT**: Procedures for performing the malaria rapid diagnostic test (RDT)

6 SUPPLIES AND EQUIPMENT

Table 1. Requisite supplies

Type	Item
**Documents**	• Laboratory requisition form<br>• Health Unit sample record form<br>• Laboratory RDT sample placement form<br>• Laboratory filter paper placement form<br>• (Electronic document) Sample control in the laboratory
**Items for collection, transport and storage**	• Whatman Grade CF12 (ref. WHA10538018, slides 580x580mm) cut to slides sized 76x30mm<br>• Ziplock bags for individual samples (minimum length 80mm to the zip)<br>• Large Ziplock bags<br>• Lancets<br>• Silica gel<br>• Cotton balls<br>• Band aids<br>• Incinerator box<br>• Disposable gowns<br>• Freezer<br>• Refrigerator

For peer review only - http://bmjopen.bmj.com/site/about/guidelines.xhtml

	• Gloves (HI-CARE 2023-05)<br>• 8 sample identification numbers<br>• Alcohol (70%)<br>• Masks<br>• Sample transport cases<br>• Box for ground transport of samples
**Office supplies**	• Staples and staplers<br>• Markers (sharpie)<br>• Pens / Pencils<br>• 1 Printer<br>• Computer<br>• Toner<br>• Notebook<br>• Clipboard

7 PROCEDURE

7.1 Sample collection

7.1.1 Collection of blood sample on filter paper

- For each participant, four (4) circular capillary blood spots of approximately 50µl (equivalent to a diameter of 1.5-2cm) will be put on two Whatman CF12 filter papers, with two spots on each paper (**Figure 1**) in accordance with the following steps:
  1) Prepare 2 papers (76x30mm), alcohol (70%), cotton balls, sterile lancet.
  2) Clean the finger with a cotton ball soaked in 70% alcohol and wait for it to dry; it is recommended to use the middle or ring finger.
  3) Remove the protector part to release the sterile lancet.
  4) Firmly prick the side of the fingertip.
  5) Carefully squeeze and wipe the first drop of blood with a dry cotton ball.
  6) Let one or two drops of blood drip onto the filter paper for each blood spot in a diameter of approximately 1.5-2cm (**Figure 1**). It is important not to let the finger touch the filter paper, to avoid contamination; only a drop of blood may touch the paper.
  7) Wipe the fingertip with another cotton ball soaked in alcohol.

> NOTE: In case you collect a sample from a baby, and it is not possible to obtain two drops of blood from one of their fingers, alternatively, you can prick their heel. In this case, only one drop of blood on the filter paper is needed.

Figure 1. Placement of blood spots on the filter paper.

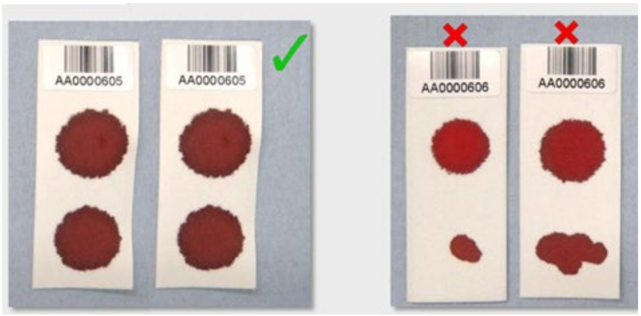

- Identify each sample with a sample identification number (the same sample identification number for each of the two filter papers) and include the collection date of the sample and the study acronym, using a pen.

For peer review only - http://bmjopen.bmj.com/site/about/guidelines.xhtml

- Put the same sample identification number on the **Laboratory analysis requisition form** (current version of POP_MAL_001_A01_PT) and fill out the form with the patient's details.
- Keep the remaining sample identification numbers stapled to each sample order for use at CISM.
- Record the sample collection data in the **Health Unit sample record form** (current version of POP_MAL_001_A02_PT).
- After blood collection, the filter paper must be dried at room temperature for 24 hours, in a safe, dry, cool and ventilated place (air conditioning can be used or the windows of the room can be opened, depending on the conditions of the site).
    - The drying surface, which can be a bench, cabinet or shelf, must be easy to clean and disinfect;
    - Avoid direct exposure to the sun or heat;
    - Do not allow samples from different patients to overlap, to avoid contamination;
    - When the process of drying is complete, the dried blood spots will be darker than the fresh blood spots.
- Once drying is complete, place the two filter papers from the same patient in a small Ziplock bag.
- Samples will be placed in a large Ziplock bag containing silica gel and stored in a refrigerator with a temperature between 2 to 8°C until the date of shipment to CISM, Manhiça district.
- Record the date that the samples are stored in the Health Unit refrigerator in the **Health Unit sample record form** (current version of POP_MAL_001_A02_PT).
- The respective requisitions must be kept in plastic files to be sent simultaneously with the samples.

7.1.2. RDT blood sample collection

- Blood collection for the RDT will be carried out following the **Procedures for performing the malaria rapid diagnostic test** (current version of POP_LB_PT_012_PT), also considering the manufacturer's specific instructions; do not discard the silica gel bag in the RDT package.
- Stick the sample identification number on the RDT and the same sample identification number on the **Laboratory analysis requisition form** (current version of POP_MAL_001_A01_PT). Write the collection date and study acronym on the RDT using a pen.
- Keep the RDT in an individual Ziplock and add the silica gel bag.
- The RDTs will be placed in a large Ziplock back and stored in a refrigerator between 2 to 8°C until the shipment date to CISM, Manhiça district.
- Record the storage of the samples on the **Health Unit sample record form** (current version of POP_MAL_001_A02_PT).
- Keep the remaining sample identification numbers stapled to each sample requisition for use at CISM.

7.2 Transport of filter papers to the CISM Laboratory

- Study personnel will contact the CISM study leader to prepare the shipment.
- Shipment logistics will be organised as follows:
  For land transport:
    - ambient temperature
    - the samples and documents will be placed in cases that must be exclusively used for this purpose (**Figure 2**)

  For air transport:
    - preferably using Portador Diário (https://www.portadordiario.co.mz/).
    - ambient temperature
    - the person responsible for the study will record the shipping code that will be assigned to the samples for later use at the time of collection at the final destination, as well as to monitor the location of the samples along the way
- At least one day before transport, verify the agreement between the actual number of samples and the records in the **Health Unit sample record form** (current version of POP_MAL_001_A02_PT).
- Whenever possible, the plastic boxes for transporting samples should be sanitised before and after use with soap and water, then disinfected with 70% alcohol.
- On the arranged day of transport, place samples (filter papers or RDTs), the requisition forms, the control forms and other study-specific documents in the shipping boxes.

For peer review only - http://bmjopen.bmj.com/site/about/guidelines.xhtml

Figure 2. Case for transporting samples (filter papers and RDTs) by land

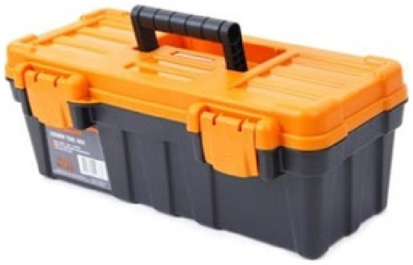

7.3 Receipt of samples at CISM

- Dried blood samples on filter paper for RDTs will be received at the CISM Laboratory, along with a laboratory requisition form.
- The Laboratory reception will verify the agreement between the sample identification numbers of the samples and the respective laboratory requisitions, and whether the number of samples received corresponds to the number of requisition forms.
- After verifying that everything is in order, the request will be entered into the SERVOLAB system, if not, the coordinator responsible must be informed so that they may follow up until the situation is resolved.

> NOTE: Samples without a laboratory requisition from partners will go through the laboratory reception for verification, however, these will not be entered into SERVOLAB due to insufficient data. The verification of these samples will be carried out together with the person responsible for the study, who must fill out the Excel Database **Control of samples in the laboratory** (current version of POP_MAL_001_A05_PT). This Excel document will be archived in electronic format and shared with the study team.

7.4 Storage of samples in the molecular biology laboratory

7.4.1. Storage of filter papers

- For storage, the two filter papers in each bag will be wrapped with aluminium foil and will be properly identified with the study name, bag number and group (A or B) using a permanent marker; the sample identification number will be stuck onto the aluminium foil;
- Place samples A in a large Ziplock bag (up to 100 filter papers) and samples B in a B Bag (up to 100 filter papers); then add 100g of silica gel to each bag (**Figure 3**).
- During the wrapping process, the **Filter paper placement form** (current version of POP_MAL_001_A03_PT) will be filled out simultaneously, which will then be verified by the technician responsible.
- The bags will be identified externally with the study name, bag group number (A or B) using a permanent marker and a paper containing the same information will be placed inside the bag.

Figure 3. Identification and storage of filter papers in the laboratory

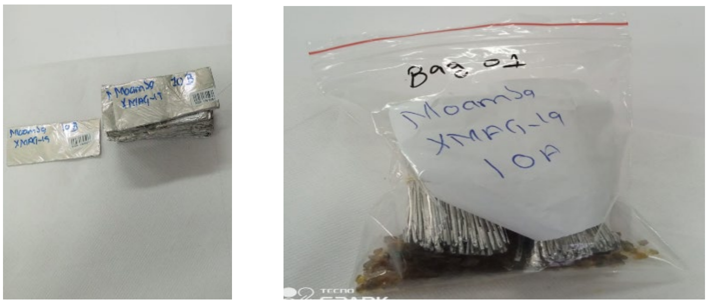

- Store A and B bags in a -20 degree freezer.

For peer review only - http://bmjopen.bmj.com/site/about/guidelines.xhtml

- The placement of the filter papers must be indicated in SERVOLAB (Seroteca Servolab>Type of Box 10x10->Box Name->filter paper bag X->placement).
- Lastly, fill out the Excel Database **Control of samples in the laboratory** (current version of POP_MAL_001_A05_PT).

7.4.2. Storage of RDTs

- For storage, the RDTs will be wrapped with aluminium foil and a sample identification number will be stuck onto the aluminium foil.
- During the wrapping process, the **RDT placement form** (current version of POP_MAL_001_A04_PT) will simultaneously be filled out, and then checked by the technician responsible.
- Place the samples from the same placement sheet in a bag (20 RDTs), then add 20g of silica gel.
- The bag will be identified externally with the study name, bag group number using a permanent marker and a paper containing the same information will be placed inside the bag.
- Place the bag of samples in a -20 degree freezer.
- The placement of the RDTs must be indicated on SERVOLAB (Seroteca Servolab>Type of Box 10x10->Box Name->RDT bag X->placement).
- Lastly, fill out the Excel Database **Control of samples in the laboratory** (current version of POP_MAL_001_A05_PT).

peer review only

For peer review only - http://bmjopen.bmj.com/site/about/guidelines.xhtml

Appendix 10. COVID-19 safety and research considerations.

1. COVID19-related biosafety capacities: This project will not involve the use of SARS-CoV-2 for any purpose, as we will focus on the detection of malaria molecular markers for surveillance and research purposes. The only samples that will be collected and managed in this project will be dried blood spots, obtained from individuals in the community and pregnant women at antenatal clinics, which will minimize the risk of COVID-19 infection among health workers and laboratory staff during sample collection and processing, respectively. All personnel involved in the study will be trained in the most up to date Malaria Consortium procedures for infection prevention and control. A daily monitoring of the health personnel involved will be conducted through the measurement of axillary temperature and identification of respiratory symptoms. In case of clinical signs, domiciliary isolation and COVID-19 testing will be recommended. CISM has developed a biosafety plan considering the following considerations:

Before starting any project-related activity, a new risk assessment will be completed using the template provided by WHO at their last version of the "Laboratory biosafety guidance related to coronavirus disease (COVID-19): interim guidance" (https://apps.who.int/iris/handle/10665/331500).

2. Collection of specimens: Finger or heel prick bloods will be collected from pregnant women at antenatal clinics and individuals in the community by community health workers. No nasopharyngeal nor oropharyngeal swabs will be collected. Samples will be collected following biosafety WHO guidelines (use of personal protective equipment [PPE]: N95 or KF94 mask, disposable gloves, protective clothing, eye protection and frequent hand washing) as described in WHO guidance on specimen collection, processing and laboratory testing: https://www.who.int/publicationsdetail/laboratory-testing-for-2019-novel-coronavirus-in-suspected-human-cases-20200117, and biosafety procedures https://apps.who.int/iris/bitstream/handle/10665/331138/WHO-WPE-GIH-2020.1-eng.pdf.

3. Laboratory biosafety: All biological samples for molecular assays will be managed at CISM. Given the nature of the samples (dried blood spot), there is a minimal risk of producing aerosols. In general, de-capping is considered a low-risk procedure. However, it depends on the design of the lid and container. Whether to proceed with the testing will be determined following a risk assessment, which considers the need for centrifugation, mixing, and aliquoting. In addition, the use of a BSC will be considered at any time when there is a high risk. All risky procedures will be carried out in a validated class II Biosafety cabinet.

4. Emergency/incident response plan: Contingency plans will be developed to reduce the likelihood of exposure to/release of a biological agent, or to reduce the consequences of such incidents by providing specific standard operating procedures (SOPs) to be followed in possible emergency scenarios that apply to the work and local environment. Personnel will be trained on these procedures and have periodic refresher training to maintain competency. First-aid kits, including medical supplies such as bottled eye washes and bandages, will be available and easily accessible to personnel. All incidents will be reported to the appropriate personnel in a timely manner. A written record of accidents and incidents will be maintained. Any incident will be reported and investigated in a timely manner and used for updating laboratory procedures and emergency response plans. Spill kits, including disinfectant, will be easily accessible to personnel. Written procedures for cleaning and decontaminating spills will be developed for the laboratory and followed by suitably trained personnel.

5. COVID-19 prevention: To avoid contamination and or and the spread of the infection, all field personnel will be provided with personal protective material for COVID 19, including face masks and/or visors and alcohol gel. Soap will also be distributed to the health facilities for use by patients.

For peer review only - http://bmjopen.bmj.com/site/about/guidelines.xhtml

Appendix 11. Worksheet for monitoring and evaluation of field activities.

**Date**
**Site**
Field
Health Facility
**Samples**
Filter paper
Rapid diagnostic test
**Quality**
Quality of the filter paper
Witness the collection process if applicable
Quantity of blood collected
Quality of the blood collected
Identification of the samples
**Cross-check sample data vs questionnaire and control sheet**
Compatible identification
**Information in source document**
Legibility of Information
Data filled in the right place
Confirm data with original document if applicable
**Total number of documents**
Questionnaire
Informed consent
Requisition form
**Total number of samples**
Filter paper
RDT
Number of discrepant RDT results
**Number of non-compliant documents**
Questionnaire
Informed consent
Requisition form
**Quantity of samples with non-conformity**
Filter paper
RDT
**Evaluated by**
Name
Date
**Revised by**
Name
Date of the next monitoring
